# Ratio estimators of intervention effects on event rates in cluster randomized trials

**DOI:** 10.1002/sim.9226

**Published:** 2021-10-15

**Authors:** Xiangmei Ma, Paul Milligan, Kwok Fai Lam, Yin Bun Cheung

**Affiliations:** ^1^ Centre for Quantitative Medicine Duke‐NUS Medical School Singapore Singapore; ^2^ Faculty of Epidemiology and Population Health London School of Hygiene & Tropical Medicine London UK; ^3^ Department of Statistics and Actuarial Science University of Hong Kong Hong Kong China; ^4^ Programme in Health Services & Systems Research Duke‐NUS Medical School Singapore Singapore; ^5^ Tampere Center for Child, Adolescent and Maternal Health Research Tampere University Tampere Finland

**Keywords:** cluster randomized trial, event rate, incidence rate ratio, ratio estimator, relative incidence

## Abstract

We consider five asymptotically unbiased estimators of intervention effects on event rates in non‐matched and matched‐pair cluster randomized trials, including ratio of mean counts $\left(r_{1}\right)$, ratio of mean cluster‐level event rates $\left(r_{2}\right)$, ratio of event rates $\left(r_{3}\right)$, double ratio of counts $\left(r_{4}\right)$, and double ratio of event rates $\left(r_{5}\right)$. In the absence of an indirect effect, they all estimate the direct effect of the intervention. Otherwise, $r_{1}$, $r_{2},$ and $r_{3}$ estimate the total effect, which comprises the direct and indirect effects, whereas $r_{4}$ and $r_{5}$ estimate the direct effect only. We derive the conditions under which each estimator is more precise or powerful than its alternatives. To control bias in studies with a small number of clusters, we propose a set of approximately unbiased estimators. We evaluate their properties by simulation and apply the methods to a trial of seasonal malaria chemoprevention. The approximately unbiased estimators are practically unbiased and their confidence intervals usually have coverage probability close to the nominal level; the asymptotically unbiased estimators perform well when the number of clusters is approximately 32 or more per trial arm. Despite its simplicity, $r_{1}$ performs comparably with $r_{2}$ and $r_{3}$ in trials with a large but realistic number of clusters. When the variability of baseline event rate is large and there is no indirect effect, $r_{4}$ and $r_{5}$ tend to offer higher power than $r_{1}$, $r_{2},$ and $r_{3}$. We discuss the implications of these findings to the planning and analysis of cluster randomized trials.

## 1 INTRODUCTION

The cluster randomized trial (CRT) is an important study design in medical and health research.^1^, ^2^, ^3^ Data on outcome events may be collected by passive surveillance or active surveillance.^4^ Passive surveillance methods may or may not provide data at the individual level. That is, they may determine only the number of events in a cluster, without identifying which individual members of the cluster experienced the events. Furthermore, the denominators for standard practice of calculating event rates may not be available.^4^ The advantage of passive surveillance is that the monetary and opportunity cost of data collection can be much reduced.

Broadly speaking, there are two approaches to the analysis of CRTs: individual‐level analysis and cluster‐level analysis. Methods for individual‐level analysis of CRTs include random‐effects models and generalized estimating equations. As compared to cluster‐level analysis, individual‐level analysis has the relative advantage of efficiency and ease in covariate adjustment. However, it has the relative disadvantage of being less robust, especially when the number of clusters is small.^1^ Furthermore, data collection by passive surveillance may not be compatible with individual‐level analysis. In this manuscript we consider only cluster‐level analysis.

An estimator of the intervention effect in terms of incidence rate ratio, also called relative incidence, that only uses event data is a ratio of the arithmetic mean of the number of outcome events per cluster in the intervention arm to that in the control arm. We call this the “ratio of mean counts”, denoted by $r_{1}$.

The denominator of event rates, that is, units of person‐time, in CRTs is usually variable across clusters. The person‐time for estimation of an event rate is sometimes approximated by the population size at some point of the study duration. In this article we use the phrases person‐time and population size interchangeably. Typical statistical practice makes comparison of event rates instead of mean number of events between trial arms. It requires extra resources in the collection of person‐time data. A demographic surveillance system, a population census, or rounds of community surveys may be required for this purpose. With both the number of events and person‐time collected for each cluster, one may calculate a cluster‐level event rate for each cluster, denoted by $c_{\mathrm{ij}}=y_{\mathrm{ij}}/p_{\mathrm{ij}}$, where $y_{\mathrm{ij}}$ and $p_{\mathrm{ij}}$ are the number of events and person‐time in the *j*th cluster in the *i*th trial arm, respectively. Then, the arithmetic means of the cluster‐level event rates in the intervention and control arms are calculated, denoted by ${\bar{c}}_{1}$ and ${\bar{c}}_{0}$, respectively. The ratio of the two means, $r_{2}={\bar{c}}_{1}/{\bar{c}}_{0}$ is a popular estimator of the incidence rate ratio.^1^, ^5^, ^6^ This estimator has been evaluated by simulations but not analytically. One simulation study considered scenarios of, approximately, $\mathrm{CV}\left(c_{\mathrm{ij}}\right)$ = 0, 0.125, and 0.25, where $\mathrm{CV}\left(c_{\mathrm{ij}}\right)$ is the coefficient of variation of the cluster‐level event rate in the *i*th trial arm and $\mathrm{CV}\left(c_{0j}\right)$ is known as “*k*” in the literature.^5^ It found little bias in $r_{2}$. However, another simulation study considered a broader range of $\mathrm{CV}\left(c_{\mathrm{ij}}\right)$.^6^ It showed that $r_{2}$ was practically unbiased when $\mathrm{CV}\left(c_{\mathrm{ij}}\right)$ = 0.05 and 0.15, but it was biased when $\mathrm{CV}\left(c_{\mathrm{ij}}\right)$ = 0.4. Analytical investigation and simulation evaluation in a broad range of parameter values are warranted.

An alternative estimator of incidence rate ratio can be obtained by first calculating the event rate in each trial arm as the sum of the number of events divided by the sum of person‐time over the clusters,^7^, ^8^ and then calculate the ratio of these event rate estimates between the trial arms. We call this the “ratio of event rates”, denoted by $r_{3}$. While ${\bar{c}}_{i}$ (*i* = 0,1) is an unweighted average of cluster‐level event rates in the *i*th trial arm, the alternative estimator of event rate here can be seen as a weighted average of cluster‐level event rates, with the clusters' population sizes as weights.

In CRTs, interventions are often provided only to a specific group of the cluster members instead of all cluster members. For example, in studies of vaccines for pediatric infectious diseases, usually only young children in a specific age range are offered the interventions or its control comparators. Older children and adults are not. We refer to the two groups of cluster members as the target and non‐target groups. The outcome events may occur in both groups. In studies based on passive surveillance, the event data may be collected for the non‐target group in addition to the target group without much additional resources required, because the capital cost and infrastructure are already invested for the target group anyway. We consider an estimator that we call “double ratio of counts”, denoted by $r_{4}$, by replacing the sums of person‐time in $r_{3}$ by the sums of number of events in the non‐target groups. Note that this estimator is defined even if the number of events in the non‐target group is zero in some clusters, which is a realistic situation because usually the reason of it being a non‐target group is that the disease incidence is relatively low. The motivation for considering this estimator arises from not only concerns of feasibility and cost of data collection but also concerns of precision and power. From an epidemiological point of view, sometimes we anticipate that the event counts in the target and non‐target groups are highly correlated, because they are both the manifestation of the disease burden in the clusters. In particular, some events are highly localized, for example, infectious diseases occurring in small outbreaks. For such events, the correlation between number of events in the target and non‐target groups, say children inside and outside a vaccination age range, is likely to be much stronger than the correlation between the number of events and amount of person‐time in the target group. This advantage in correlation offers a potential for improved precision. Note that $r_{4}$ and the three estimators aforementioned have different targets of estimation (estimands): $r_{1}$, $r_{2},$ and $r_{3}$ estimate the total effect of the intervention whereas $r_{4}$ estimates the direct effect. Details will be discussed in the next section.

We also propose a new estimator that we call “double ratio of event rates”, denoted by $r_{5}$. It has the ratio of event rates between the target and non‐target groups in the intervention arm as the numerator and its counterpart in the control arm as the denominator. Details in statistical notations will be provided in the next section. We hypothesize that this estimator will out‐perform $r_{4}$ in precision and power.

Donner and Klar pointed out that CRTs of binary outcomes may regard a proportion as a ratio and then an appropriate variance estimate can be obtained from sample survey theory.^9^ They used the ratio of this estimated variance to the estimated binomial variance to adjust the Chi‐square statistics for hypothesis testing. In the context of toxicological experiments in which litters of animals were the experimental units and a binary outcome was observed for each animal, Rao and Scott proposed using the aforementioned approach to adjust the Chi‐square and Cochran‐Armitage statistics.^10^ There has been some subsequent research on using ratio estimators for CRTs with event rate outcomes, including the two simulation studies of $r_{2}$ aforementioned.^5^, ^6^ Furthermore, Dufault and Jewell proposed permutation tests of counts of events only, with or without adjustment for differential ascertainment.^4^ All of them concerned only CRTs that randomize clusters individually and (implicitly) aim to estimate the total effect.

This study aims to (a) evaluate and compare the performance of the five estimators aforementioned and (b) develop, evaluate, and compare bias‐corrected version of them. In Section 2 we will analytically assess and develop the methods. In Section 3 we will evaluate the methods by simulation in a broad range of realistic scenarios. In Section 4 we will apply the methods to a study of seasonal malaria chemoprevention. Section 5 gives some concluding remarks.

For brevity, we will focus on CRTs that randomize clusters individually, that is, non‐matched CRTs. Where necessary we also provide the details for matched‐pair CRTs in which one cluster per matched pair is randomized to receive the intervention and the other serves as the control. Introduction to the two types of CRTs can be found in, for example, Hayes and Moulton^1^ and Donner and Klar.^2^

## 2 STATISTICAL METHODS

### 2.1 Intervention effects and event rates

An intervention may have a direct effect and an indirect effect, for example, via reducing disease transmission in the community.^3^, ^11^ Only the intervention's target group can benefit from the direct effect; both the target and non‐target groups may benefit from the indirect effect, if any. Assume that:

$$\beta_{T}=\beta_{D}+\beta_{I}$$

where $\beta_{T}$, $\beta_{D},$ and $\beta_{I}$ are the total, direct, and indirect effects in terms of log incidence rate ratio. If there is no indirect effect, $\beta_{I}=0$ and $\beta_{T}=\beta_{D}$. The presence of an indirect effect depends on various factors including the nature of the interventions and outcome events. For example, even though vaccines are often anticipated to generate some degree of indirect effect on efficacy endpoints, they are usually anticipated to have no indirect effect on safety endpoints.

Let $Y_{\mathrm{ijk}}$ and $P_{\mathrm{ijk}}$ be the number of events and total person‐time in the *k*th group of the *j*th cluster in the *i*th trial arm in the population, respectively, where *k* = 1 and 0 represent the target and non‐target groups, respectively, *i* = 1 and 0 represent intervention and control trial arms, respectively. We consider a data generating process that is often used in epidemiologic modeling, that the expected value of $Y_{\mathrm{ijk}}$ given $P_{\mathrm{ijk}}$ is:

(1) $$E\left(Y_{\mathrm{ijk}}|P_{\mathrm{ijk}}\right)=\exp\left(\beta_{D,\mathrm{ik}}+\beta_{I,i}+\alpha_{\mathrm{ij}}+\gamma_{k}\right)P_{\mathrm{ijk}},$$

where $\beta_{D,\mathrm{ik}}$ and $\beta_{I,i}$ represent direct and indirect effects ($\beta_{D,11}=\beta_{D}$; $\beta_{I,1}=\beta_{I}$; $\beta_{D,10}=\beta_{D,01}=\beta_{D,00}=\beta_{I,0}=0$), $\alpha_{\mathrm{ij}}$ is a random cluster effect with standard deviation $\mathrm{SD}\left(\alpha_{\mathrm{ij}}\right)$ ≥ 0 that represents variation in event rates between clusters within each trial arm, and $\gamma_{k}$ represents the difference in event rates between the target and non‐target group ($\gamma_{1}=\gamma$; $\;\gamma_{0}=0$). Note that $\mathrm{SD}\left(\alpha_{\mathrm{ij}}\right)≅\mathrm{CV}\left(c_{\mathrm{ij}}\right)$, the coefficient of variation in cluster‐level event rate.^5^, ^6^ By randomization, the distributions of $\alpha_{\mathrm{ij}}$ and $P_{\mathrm{ijk}}$ are identical in expectation between the intervention and control arms. From Equation (1), $\mathrm{CV}\left(Y_{\mathrm{ijk}}\right)=\mathrm{CV}\left(P_{\mathrm{ijk}}\right)$ and $\text{corr}\left(Y_{\mathrm{ijk}},P_{\mathrm{ijk}}\right)=1$ if $\mathrm{SD}\left(\alpha_{\mathrm{ij}}\right)=0$. The difference $\mathrm{CV}\left(Y_{\mathrm{ijk}}\right)-\mathrm{CV}\left(P_{\mathrm{ijk}}\right)$ increases and the correlation $\text{corr}\left(Y_{\mathrm{ijk}},P_{\mathrm{ijk}}\right)$ decreases as $\mathrm{SD}\left(\alpha_{\mathrm{ij}}\right)$ increases.

### 2.2 Asymptotically unbiased estimators

Given a sample dataset of $\left\{y_{\mathrm{ijk}},p_{\mathrm{ijk}}:j=1,2,\ldots ,n_{i};i=0,1;k=0,1\right\}$, with randomization and a large number of clusters per trial arm, the ratio of mean counts, $r_{1}$, provides an asymptotically unbiased estimator of the total effect that compares the event rates in the intervention and control arms:

$$r_{1}=\frac{{\bar{y}}_{1}}{{\bar{y}}_{0}}=\frac{\sum_{j=1}^{n_{1}}y_{1j1}/n_{1}}{\sum_{j=1}^{n_{0}}y_{0j1}/n_{0}}\approx \frac{\sum_{j=1}^{n_{1}}\exp\left(\beta_{D}+\beta_{I}+\alpha_{1j}+\gamma \right)p_{1j1}/n_{1}}{\sum_{j=1}^{n_{0}}\exp\left(\alpha_{0j}+\gamma \right)p_{0j1}/n_{0}}=\exp\left(\beta_{D}+\beta_{I}\right)\frac{\sum_{j=1}^{n_{1}}\exp\left(\alpha_{1j}+\gamma \right)p_{1j1}/n_{1}}{\sum_{j=1}^{n_{0}}\exp\left(\alpha_{0j}+\gamma \right)p_{0j1}/n_{0}}\approx \exp\left(\beta_{T}\right).$$

When $n_{1}$ and $n_{0}$ are small, $\left[\sum_{j=1}^{n_{1}}\exp\left(\alpha_{1j}+\gamma \right)p_{1j1}/n_{1}\right]/\left[\sum_{j=1}^{n_{0}}\exp\left(\alpha_{0j}+\gamma \right)p_{0j1}/n_{0}\right]≉1,$

causing a small sample bias in the ratio estimator.^7^ This and the bias in the other estimators will be discussed in Section 2.3.

Similarly, the ratio of mean cluster‐level event rates,

$$r_{2}=\frac{{\bar{c}}_{1}}{{\bar{c}}_{0}}=\frac{\sum_{j=1}^{n_{1}}c_{1j}/n_{1}}{\sum_{j=1}^{n_{0}}c_{0j}/n_{0}},$$

where $c_{\mathrm{ij}}=y_{\mathrm{ij}1}/p_{\mathrm{ij}1}$, and ratio of event rates,

$$r_{3}=\frac{R_{1}}{R_{0}}=\frac{\sum_{j=1}^{n_{1}}y_{1j1}/\sum_{j=1}^{n_{1}}p_{1j1}}{\sum_{j=1}^{n_{0}}y_{0j1}/\sum_{j=1}^{n_{0}}p_{0j1}},$$

also provide asymptotically unbiased estimators of the total effect.

The variance of $r_{1}$, $r_{2},$ and $r_{3}$ can be written as:^5^, ^7^, ^8^

(2) $$\mathrm{Var}\left(r_{1}\right)=r_{1}^{2}\left[\frac{\mathrm{CV}{\left(y_{1j1}\right)}^{2}}{n_{1}}+\frac{\mathrm{CV}{\left(y_{0j1}\right)}^{2}}{n_{0}}\right],$$

(3) $$\mathrm{Var}\left(r_{2}\right)=r_{2}^{2}\left[\frac{\mathrm{Var}\left({\bar{c}}_{1}\right)}{{\bar{c}}_{1}^{2}}+\frac{\mathrm{Var}\left({\bar{c}}_{0}\right)}{{\bar{c}}_{0}^{2}}\right],$$

and

(4) $$\mathrm{Var}\left(r_{3}\right)=r_{3}^{2}\left[\frac{\mathrm{Var}\left(R_{1}\right)}{R_{1}^{2}}+\frac{\mathrm{Var}\left(R_{0}\right)}{R_{0}^{2}}\right].$$

Furthermore, in first‐order Taylor series expansion, $\mathrm{Var}\left({\bar{c}}_{i}\right)=\mathrm{Var}\left(R_{i}\right)$,^8^ and

$$\mathrm{Var}\left(R_{i}\right)=\frac{R_{i}^{2}}{n_{i}}\left[\mathrm{CV}{\left(y_{\mathrm{ij}1}\right)}^{2}+\mathrm{CV}{\left(p_{\mathrm{ij}1}\right)}^{2}-2\mathrm{CV}\left(y_{\mathrm{ij}1}\right)\mathrm{CV}\left(p_{\mathrm{ij}1}\right)\text{corr}\left(y_{\mathrm{ij}1},p_{\mathrm{ij}1}\right)\right].$$

From Equation (1), $\mathrm{CV}\left(y_{\mathrm{ijk}}\right)\geq \mathrm{CV}\left(p_{\mathrm{ijk}}\right)$. Let $\mathrm{CV}\left(p_{\mathrm{ijk}}\right)=\theta_{i}\times \mathrm{CV}\left(y_{\mathrm{ijk}}\right)$, where $0<\theta_{i}\leq 1$.

If $r_{1}$ and $r_{2}$ give the same sample estimate, a comparison of $\mathrm{Var}\left(r_{1}\right)$ and $\mathrm{Var}\left(r_{2}\right)$ boils down to an evaluation of whether

$$\begin{array}{ll} n_{i}\left[\frac{\mathrm{Var}\left({\bar{c}}_{i}\right)\;}{{\bar{c}}_{i}^{2}}-\frac{\mathrm{CV}{\left(y_{\mathrm{ij}1}\right)}^{2}}{n_{i}}\right] & \approx n_{i}\left[\frac{\mathrm{Var}\left(R_{i}\right)\;}{{\bar{c}}_{i}^{2}}-\frac{\mathrm{CV}{\left(y_{\mathrm{ij}1}\right)}^{2}}{n_{i}}\right]\\ & ={\left(\frac{R_{i}}{{\bar{c}}_{i}}\right)}^{2}\left[\mathrm{CV}{\left(y_{\mathrm{ij}1}\right)}^{2}+\mathrm{CV}{\left(p_{\mathrm{ij}1}\right)}^{2}-2\mathrm{CV}\left(y_{\mathrm{ij}1}\right)\mathrm{CV}\left(p_{\mathrm{ij}1}\right)\text{corr}\left(y_{\mathrm{ij}1},p_{\mathrm{ij}1}\right)\right]-\mathrm{CV}{\left(y_{\mathrm{ij}1}\right)}^{2}\\ & ={\left(\frac{R_{i}}{{\bar{c}}_{i}}\right)}^{2}\left[\left(1+\theta_{i}^{2}-2\theta_{i}\text{corr}\left(y_{\mathrm{ij}1},p_{\mathrm{ij}1}\right)\right)\mathrm{CV}{\left(y_{\mathrm{ij}1}\right)}^{2}\right]-\mathrm{CV}{\left(y_{\mathrm{ij}1}\right)}^{2}<0. \end{array}$$

Therefore, $\mathrm{Var}\left(r_{2}\right)<\mathrm{Var}\left(r_{1}\right)$ under the condition that in both trial arms:

(5) $$\text{corr}\left(y_{\mathrm{ij}1},p_{\mathrm{ij}1}\right)>\frac{1+\theta_{i}^{2}-{\left(\frac{{\bar{c}}_{i}}{R_{i}}\right)}^{2}}{2\theta_{i}}.$$

Similarly, if $r_{1}$ and $r_{3}$ give the same estimate, $\mathrm{Var}\left(r_{3}\right)<\mathrm{Var}\left(r_{1}\right)$ under the condition that in both trial arms:

(6) $$\text{corr}\left(y_{\mathrm{ij}1},p_{\mathrm{ij}1}\right)>\theta_{i}/2.$$

If $r_{2}$ and $r_{3}$ give the same estimate, $\mathrm{Var}\left(r_{2}\right)<\mathrm{Var}\left(r_{3}\right)$ under the condition that in both trial arms $\mathrm{Var}\left(R_{i}\right)/{\bar{c}}_{i}^{2}<\mathrm{Var}\left(R_{i}\right)/R_{i}^{2}$, that is,

(7) $${\bar{c}}_{i}/R_{i}>1.$$

If $\text{corr}\left(y_{\mathrm{ij}1},p_{\mathrm{ij}1}\right)=0$, ${\bar{c}}_{i}/R_{i}>1$ by Jensen's inequality. As we will see in the case study in Section 4, it is possible that $\text{corr}\left(y_{\mathrm{ij}1},p_{\mathrm{ij}1}\right)$ approximately equals zero in real‐world situations.

In contrast, with large $n_{1},n_{0}$ and randomization, the double ratio of counts ($r_{4}$) provides an asymptotically unbiased estimator of the direct effect:

$$r_{4}=\frac{R_{1}^{*}}{R_{0}^{*}}=\frac{\sum_{j=1}^{n_{1}}y_{1j1}/\sum_{j=1}^{n_{1}}y_{1j0}}{\sum_{j=1}^{n_{0}}y_{0j1}/\sum_{j=1}^{n_{0}}y_{0j0}}$$

$$\approx \frac{\sum_{j=1}^{n_{1}}\exp\left(\beta_{D}+\beta_{I}+\alpha_{1j}+\gamma \right)p_{1j1}/\sum_{j=1}^{n_{1}}\exp\left(\beta_{I}+\alpha_{1j}\right)p_{1j0}}{\sum_{j=1}^{n_{0}}\exp\left(\alpha_{0j}+\gamma \right)p_{0j1}/\sum_{j=1}^{n_{0}}\exp\left(\alpha_{0j}\right)p_{0j0}}=\exp\left(\beta_{D}\right).$$

The variance of $r_{4}$ is:

(8) $$\mathrm{Var}\left(r_{4}\right)=r_{4}^{2}\left[\frac{\mathrm{Var}\left(R_{1}^{*}\right)}{{\left(R_{1}^{*}\right)}^{2}}+\frac{\mathrm{Var}\left(R_{0}^{*}\right)}{{\left(R_{0}^{*}\right)}^{2}}\right],$$

where

$$\frac{\mathrm{Var}\left(R_{i}^{*}\right)}{{\left(R_{i}^{*}\right)}^{2}}=\frac{1}{n_{i}}\left[\mathrm{CV}{\left(y_{\mathrm{ij}1}\right)}^{2}+\mathrm{CV}{\left(y_{\mathrm{ij}0}\right)}^{2}-2\mathrm{CV}\left(y_{\mathrm{ij}1}\right)\mathrm{CV}\left(y_{\mathrm{ij}0}\right)\text{corr}\left(y_{\mathrm{ij}1},y_{\mathrm{ij}0}\right)\right].$$

Comparisons of the variances of the estimators $r_{2}$ vs $r_{4}$ and $r_{3}$ vs $r_{4}$ are meaningful only if the indirect effect is absent or trivial and the estimates $r_{2}≅r_{4}$ and $r_{3}≅r_{4}$. In Section A of Online Supplementary Material 1 we show that $\;\mathrm{Var}\left(r_{4}\right)<\mathrm{Var}\left(r_{2}\right)$ if in both trial arms:

(9) $$\text{corr}\left(y_{\mathrm{ij}1},y_{\mathrm{ij}0}\right)-{\left(\frac{R_{i}}{{\bar{c}}_{i}}\right)}^{2}\theta_{i}\text{corr}\left(y_{\mathrm{ij}1},p_{\mathrm{ij}1}\right)>1-{\left(\frac{R_{i}}{{\bar{c}}_{i}}\right)}^{2}\times \frac{1+\theta_{i}^{2}}{2}.$$

Similarly, $\mathrm{Var}\left(r_{4}\right)<\mathrm{Var}\left(r_{3}\right)$ if in both trial arms:

(10) $$\text{corr}\left(y_{\mathrm{ij}1},y_{\mathrm{ij}0}\right)-\theta_{i}\text{corr}\left(y_{\mathrm{ij}1},p_{\mathrm{ij}1}\right)>\frac{1-\theta_{i}^{2}}{2}.$$

A strong correlation between number of events in the target and non‐target groups as compared to the correlation between the number of events and person‐time in the target group would favor $r_{4}$ over $r_{2}$ and $r_{3}$ in terms of precision.

If a non‐trivial indirect effect is present, the absolute values of the test statistics

$$|t\left(r_{2}\right)|=\frac{|r_{2}-1|}{\sqrt{\mathrm{Var}\left(r_{2}\right)}},$$

$$|t\left(r_{3}\right)|=\frac{|r_{3}-1|}{\sqrt{\mathrm{Var}\left(r_{3}\right)}}$$

and

$$|t\left(r_{4}\right)|=\frac{|r_{4}-1|}{\sqrt{\mathrm{Var}\left(r_{4}\right)}}$$

are comparable in the sense that they all indicate the probability of rejecting the null hypothesis of the target ratio being one. Let $r_{2}=\zeta r_{4}$, then $\left|t\left(r_{2}\right)\right|<\left|t\left(r_{4}\right)\right|$ if in both trial arms (details in Online Supplementary Material 1):

(11) $$\text{corr}\left(y_{\mathrm{ij}1},y_{\mathrm{ij}0}\right)>1-\zeta^{2}{\left(\frac{r_{4}-1}{\zeta r_{4}-1}\right)}^{2}{\left(\frac{R_{i}}{{\bar{c}}_{i}}\right)}^{2}\left[\frac{1+\theta_{i}^{2}}{2}-\theta_{i}\text{corr}\left(y_{\mathrm{ij}1},p_{\mathrm{ij}1}\right)\right].$$

Similarly, let $r_{3}=\xi r_{4}$, then $\left|t\left(r_{3}\right)\right|<\left|t\left(r_{4}\right)\right|$ if in both trial arms:

(12) $$\text{corr}\left(y_{\mathrm{ij}1},y_{\mathrm{ij}0}\right)>1-\xi^{2}{\left(\frac{r_{4}-1}{\xi r_{4}-1}\right)}^{2}\left[\frac{1+\theta_{i}^{2}}{2}-\theta_{i}\text{corr}\left(y_{\mathrm{ij}1},p_{\mathrm{ij}1}\right)\right].$$

In the special case that ζ=1 or ξ=1, Equations (11) or (12) reduce to Equations (9) and (10), respectively. Otherwise, assume that the estimates of direct and indirect effects are in the same direction, the closer ζ or ξ is to 1, the more favourable $r_{4}$ is in terms of power.

Similar to $r_{4}$, the ratio of event rates estimator, $r_{5}=R_{1}^{†}/R_{0}^{†}=\frac{R_{11}^{\prime }/R_{10}^{\prime }}{R_{01}^{\prime }/R_{00}^{\prime }}$, where $R_{\mathrm{ik}}^{\prime }=\frac{\sum_{j}y_{\mathrm{ijk}}}{\sum_{j}p_{\mathrm{ijk}}}$, also provides an asymptotically unbiased estimator of the direct effect. The variance of $r_{5}$ is:

(13) $$\mathrm{Var}\left(r_{5}\right)=r_{5}^{2}\left[\frac{\mathrm{Var}\left(R_{1}^{†}\right)}{R_{1}^{†2}}+\frac{\mathrm{Var}\left(R_{0}^{†}\right)}{R_{0}^{†2}}\right]$$

where

$$\mathrm{Var}\left(R_{i}^{†}\right)=\mathrm{Var}\left(\frac{R_{i1}^{\prime }}{R_{i0}^{\prime }}\right)=R_{i}^{†2}\left(\frac{\mathrm{Var}\left(R_{i1}^{\prime }\right)}{R_{i1}^{\prime 2}}+\frac{\mathrm{Var}\left(R_{i0}^{\prime }\right)}{R_{i0}^{\prime 2}}-\frac{2\mathrm{cov}\left(R_{i1}^{\prime },\;R_{i0}^{\prime }\right)}{R_{i1}^{\prime }R_{i0}^{\prime }}\right),$$

$$\frac{\mathrm{Var}\left(R_{\mathrm{ik}}^{\prime }\right)}{R_{\mathrm{ik}}^{\prime 2}}=\frac{1}{n_{i}}\left[\mathrm{CV}{\left(y_{\mathrm{ijk}}\right)}^{2}+\mathrm{CV}{\left(p_{\mathrm{ijk}}\right)}^{2}-2\mathrm{CV}\left(y_{\mathrm{ijk}}\right)\mathrm{CV}\left(p_{\mathrm{ijk}}\right)\text{corr}\left(y_{\mathrm{ijk}},p_{\mathrm{ijk}}\right)\right],$$

$$\mathrm{cov}\left(R_{i1}^{\prime },R_{i0}^{\prime }\right)=\frac{1}{n_{i}{\bar{p}}_{i\cdot 1}{\bar{p}}_{i\cdot 0}}\left\{\mathrm{cov}\left(y_{\mathrm{ij}1},y_{\mathrm{ij}0}\right)+R_{i1}^{\prime }R_{i0}^{\prime }\mathrm{cov}\left(p_{\mathrm{ij}1},p_{\mathrm{ij}0}\right)-R_{i0}^{\prime }\mathrm{cov}\left(y_{\mathrm{ij}1},p_{\mathrm{ij}0}\right)-R_{i1}^{\prime }\mathrm{cov}\left(y_{\mathrm{ij}0},p_{\mathrm{ij}1}\right)\right\},$$

and ${\bar{p}}_{i\cdot k}=\sum_{j=1}^{n_{i}}p_{\mathrm{ijk}}/n_{i}$.

Furthermore,

$$\;\frac{\mathrm{Var}\left(R_{i}^{†}\right)}{R_{i}^{†2}}=\frac{\mathrm{Var}\left(R_{i1}^{\prime }\right)}{R_{i1}^{\prime 2}}+\frac{\mathrm{Var}\left(R_{i0}^{\prime }\right)}{R_{i0}^{\prime 2}}-\frac{2\mathrm{cov}\left(R_{i1}^{\prime },\;R_{i0}^{\prime }\right)}{R_{i1}^{\prime }R_{i0}^{\prime }}$$

$$\begin{array}{ll} & =\frac{1}{n_{i}}\{\mathrm{CV}{\left(y_{\mathrm{ij}1}\right)}^{2}+\mathrm{CV}{\left(p_{\mathrm{ij}1}\right)}^{2}-2\mathrm{CV}\left(y_{\mathrm{ij}1}\right)\mathrm{CV}\left(p_{\mathrm{ij}1}\right)\text{corr}\left(y_{\mathrm{ij}1},p_{\mathrm{ij}1}\right)\\ & +\;\mathrm{CV}{\left(y_{\mathrm{ij}0}\right)}^{2}+\mathrm{CV}{\left(p_{\mathrm{ij}0}\right)}^{2}-2\mathrm{CV}\left(y_{\mathrm{ij}0}\right)\mathrm{CV}\left(p_{\mathrm{ij}0}\right)\text{corr}\left(y_{\mathrm{ij}0},p_{\mathrm{ij}0}\right)\\ & \;-\;2[\mathrm{CV}\left(y_{\mathrm{ij}1}\right)\mathrm{CV}\left(y_{\mathrm{ij}0}\right)\text{corr}\left(y_{\mathrm{ij}1},y_{\mathrm{ij}0}\right)+\mathrm{CV}\left(p_{\mathrm{ij}1}\right)\mathrm{CV}\left(p_{\mathrm{ij}0}\right)\text{corr}\left(p_{\mathrm{ij}1},p_{\mathrm{ij}0}\right)\\ & \;-\;\mathrm{CV}\left(y_{\mathrm{ij}1}\right)\mathrm{CV}\left(p_{\mathrm{ij}0}\right)\text{corr}\left(y_{\mathrm{ij}1},p_{\mathrm{ij}0}\right)-\mathrm{CV}\left(y_{\mathrm{ij}0}\right)\mathrm{CV}\left(p_{\mathrm{ij}1}\right)\text{corr}\left(y_{\mathrm{ij}0},p_{\mathrm{ij}1}\right)]\}. \end{array}$$

Then, $\mathrm{Var}\left(r_{5}\right)<\mathrm{Var}\left(r_{4}\right)$ if in both trial arms (details in Online Supplementary Material 1):

(14) $$\text{corr}\left(y_{\mathrm{ij}1},p_{\mathrm{ij}0}\right)+\text{corr}\left(y_{\mathrm{ij}0},p_{\mathrm{ij}1}\right)<\text{corr}\left(y_{\mathrm{ij}0},p_{\mathrm{ij}0}\right)+\text{corr}\left(y_{\mathrm{ij}1},p_{\mathrm{ij}1}\right).$$

It is natural to expect that the number of events is more strongly correlated with the person‐time in the same group than the other group. Therefore, we anticipate a high chance of $\mathrm{Var}\left(r_{5}\right)<\mathrm{Var}\left(r_{4}\right)$ in many studies.

For matched‐pair CRTs, $n_{1}=n_{0}=n$ is the number of pairs of clusters. Within the *j*th pair of clusters, one cluster is randomized to receive intervention (*i* = 1) and the other is the control cluster (*i* = 0). The paired design version of the five estimators, $r_{l}^{\text{paired}}\;\left(\;l=1,2,3,4,5\right)$, and their variances are shown in Appendix Table A1.

### 2.3 Approximately unbiased estimators

The literature about bias in ratio estimators and the mitigation methods has very much focused on paired observations, mostly concerning an estimator in the form of $r_{1}^{\text{paired}}$.^7^, ^12^ Rao and Pereira considered a ratio‐of‐ratio estimator in the form of $r_{3}^{\text{paired}}$ or $r_{4}^{\text{paired}}$.^13^ These previous works showed that the estimators have a bias of order $n^{-1}$; bias‐reduction methods were proposed. Useful though they are, they do not deal with non‐matched CRTs and $r_{2}^{\text{paired}}$ and $r_{5}^{\text{paired}}$.

One solution is to determine the expectation and therefore bias of a ratio estimator, and then subtract the bias from the estimator. See, for example, van Kempen and van Vliet^8^ and Rao and Pereira.^13^ Although it has only been considered in studies of paired observations, the concept is applicable to both non‐matched and matched‐pair CRTs. Following this approach, we propose a set of approximately unbiased estimators. The key results for non‐matched CRTs are shown below. Their matched‐pair counterparts and details of the derivations are available in Section B of Online Supplementary Material 1.

#### 2.3.1 Ratio of mean counts in non‐matched CRTs

The expectation of the asymptotically unbiased estimator $r_{1}$ and approximately unbiased estimator $r_{1}^{*}$ of the ratio of means estimator are, respectively:

$$E\left(r_{1}\right)\approx \frac{{\bar{Y}}_{1\cdot 1}}{{\bar{Y}}_{0\cdot 1}}\left[1+\frac{\mathrm{Var}\left(y_{0j1}\right)}{n_{0}{\bar{Y}}_{0\cdot 1}^{2}}\right],$$

$$r_{1}^{*}=r_{1}-\left[E\left(r_{1}\right)-\frac{{\bar{Y}}_{1\cdot 1}}{{\bar{Y}}_{0\cdot 1}}\right]=r_{1}-\frac{{\bar{Y}}_{1\cdot 1}}{{\bar{Y}}_{0\cdot 1}}\times \frac{\mathrm{Var}\left(y_{0j1}\right)}{n_{0}{\bar{Y}}_{0\cdot 1}^{2}}\approx r_{1}\left[1-\frac{1}{n_{0}}{\mathrm{CV}}^{2}\left(y_{0j1}\right)\right],$$

with the unknown population mean ${\bar{Y}}_{0\cdot 1}$ approximated by the sample mean ${\bar{y}}_{0\cdot 1}$ to form the sample CV.

#### 2.3.2 Ratio of mean cluster‐level event rates in non‐matched CRTs

The expectation of the asymptotically unbiased estimator $r_{2}$ and approximately unbiased estimator $r_{2}^{*}$ are, respectively:

(15) $$E\left(r_{2}\right)\approx \frac{{\bar{C}}_{1}}{{\bar{C}}_{0}}\left[1+\frac{\mathrm{Var}\left(c_{0j}\right)}{n_{0}{\bar{C}}_{0}^{2}}\right],r_{2}^{*}=r_{2}-\left[E\left(r_{2}\right)-\frac{{\bar{C}}_{1}}{{\bar{C}}_{0}}\right]\approx r_{2}\left[1-\frac{1}{n_{0}}{\mathrm{CV}}^{2}\left(c_{0j}\right)\right],$$

with the unknown population mean of cluster event rates ${\bar{C}}_{0}$ replaced by the sample estimate to form the sample CV.

#### 2.3.3 Ratio of event rates in non‐matched CRTs

$$\begin{array}{ll} & E\left(r_{3}\right)\approx \frac{{\bar{Y}}_{1\cdot 1}/{\bar{P}}_{1\cdot 1}}{{\bar{Y}}_{0\cdot 1}/{\bar{P}}_{0\cdot 1}}\left[1-\frac{\mathrm{cov}\left(y_{1j1},p_{1j1}\right)}{n_{1}{\bar{Y}}_{1\cdot 1}{\bar{P}}_{1\cdot 1}}-\frac{\mathrm{cov}\left(y_{0j1},p_{0j1}\right)}{n_{0}{\bar{Y}}_{0\cdot 1}{\bar{P}}_{0\cdot 1}}+\frac{\mathrm{Var}\left(y_{0j1}\right)}{n_{0}{\bar{Y}}_{0\cdot 1}^{2}}+\frac{\mathrm{Var}\left(p_{1j1}\right)}{n_{1}{\bar{P}}_{1\cdot 1}^{2}}\right],\\ & \;r_{3}^{*}=r_{3}-\left[E\left(r_{3}\right)-\frac{{\bar{Y}}_{1\cdot 1}/{\bar{P}}_{1\cdot 1}}{{\bar{Y}}_{0\cdot 1}/{\bar{P}}_{0\cdot 1}}\right]\\ & \;\approx r_{3}\left[1+\frac{1}{n_{1}}\mathrm{CV}\left(y_{1j1}\right)\mathrm{CV}\left(p_{1j1}\right)\text{corr}\left(y_{1j1},p_{1j1}\right)+\frac{1}{n_{0}}\mathrm{CV}\left(y_{0j1\;}\right)\mathrm{CV}\left(p_{0j1\;}\right)\text{corr}\left(y_{0j1},p_{0j1\;}\right)-\frac{1}{n_{0}}{\mathrm{CV}}^{2}\left(y_{0j1}\right)-\frac{1}{n_{1}}{\mathrm{CV}}^{2}\left(p_{1j1}\right)\right]\;, \end{array}$$

with the unknown population mean ${\bar{Y}}_{i\cdot 1}$ and ${\bar{P}}_{i\cdot 1}\;\left(i=0,1\right)$ replaced by their sample estimates to form the sample CVs.

#### 2.3.4 Double ratio of counts in non‐matched CRTs

$E\left(r_{4}\right)$ and $r_{4}^{*}$ can be obtained by replacing $r_{3}$, $p_{\mathrm{ij}1}$ and ${\bar{P}}_{i\cdot 1}$ by $r_{4}$, $y_{\mathrm{ij}0}$, and ${\bar{Y}}_{i\cdot 0}$ in the formula in the previous sub‐section on ratio of event rates.

#### 2.3.5 Double ratio of event rates in non‐matched CRTs

$$\begin{array}{ll} E\left(r_{5}\right) & \approx \frac{\frac{{\bar{Y}}_{1\cdot 1}}{{\bar{P}}_{1\cdot 1}}/\frac{{\bar{Y}}_{1\cdot 0}}{{\bar{P}}_{1\cdot 0}}\;}{\frac{{\bar{Y}}_{0\cdot 1}}{{\bar{P}}_{0\cdot 1}}/\frac{{\bar{Y}}_{0\cdot 0}}{{\bar{P}}_{0\cdot 0}}}\;\{1-\sum_{i=0}^{1}\left[\frac{\mathrm{cov}\left(y_{\mathrm{ij}1},y_{\mathrm{ij}0}\right)}{n_{i}{\bar{Y}}_{i\cdot 1}{\bar{Y}}_{i\cdot 0}}+\frac{\mathrm{cov}\left(p_{\mathrm{ij}1},p_{\mathrm{ij}0}\right)}{n_{i}{\bar{P}}_{i\cdot 1}{\bar{P}}_{i\cdot 0}}+\sum_{k=0}^{1}\sum_{k^{\prime }=0}^{1}\frac{{\left(-1\right)}^{k+k^{\prime }}}{n_{i}}\frac{\mathrm{cov}\left(y_{\mathrm{ijk}},p_{\mathrm{ij}k^{\prime }}\right)}{{\bar{Y}}_{i\cdot k}{\bar{P}}_{i\cdot k^{\prime }}}\right]\\ & \;+\frac{\mathrm{Var}\left(y_{1j0}\right)}{n_{1}{\bar{Y}}_{1\cdot 0}^{2}}+\frac{\mathrm{Var}\left(y_{0j1}\right)}{n_{0}{\bar{Y}}_{0\cdot 1}^{2}}+\frac{\mathrm{Var}\left(p_{1j1}\right)}{n_{1}{\bar{P}}_{1\cdot 1}^{2}}+\frac{\mathrm{Var}\left(p_{0j0}\right)}{n_{0}{\bar{P}}_{0\cdot 0}^{2}}\}, \end{array}$$

$$\begin{array}{ll} r_{5}^{*} & \approx r_{5}\{1+\sum_{i=0}^{1}[\frac{1}{n_{i}}\left[\mathrm{CV}\left(y_{\mathrm{ij}1}\right)\mathrm{CV}\left(y_{\mathrm{ij}0}\right)\text{corr}\left(y_{\mathrm{ij}1},y_{\mathrm{ij}0}\right)+\mathrm{CV}\left(p_{\mathrm{ij}1}\right)\mathrm{CV}\left(p_{\mathrm{ij}0}\right)\text{corr}\left(p_{\mathrm{ij}1},p_{\mathrm{ij}0}\right)\right]\\ & \;+\sum_{k=0}^{1}\sum_{k^{\prime }=0}^{1}\frac{{\left(-1\right)}^{k+k^{\prime }}}{n_{i}}\mathrm{CV}\left(y_{\mathrm{ijk}}\right)\mathrm{CV}\left(p_{\mathrm{ij}k^{\prime }}\right)\text{corr}\left(y_{\mathrm{ijk}},p_{\mathrm{ij}k^{\prime }}\right)]\;-\frac{1}{n_{0}}{\mathrm{CV}}^{2}\left(y_{0j1}\right)\;-\frac{1}{n_{0}}{\mathrm{CV}}^{2}\left(p_{0j0}\right)\;-\frac{1}{n_{1}}{\mathrm{CV}}^{2}\left(y_{1j0}\right)\;-\frac{1}{n_{1}}{\mathrm{CV}}^{2}\left(p_{1j1\;}\right)\;\}. \end{array}$$

#### 2.3.6 Variances and confidence intervals

The variance of $r_{1}^{*}$ is:

$$\mathrm{Var}\left(r_{1}^{*}\right)=E{\left[r_{1}^{*}-E\left(r_{1}^{*}\right)\right]}^{2}=E{\left\{\left[r_{1}-\frac{{\bar{Y}}_{1\cdot 1}}{{\bar{Y}}_{0\cdot 1}}\times \frac{\mathrm{Var}\left(y_{0j1}\right)}{n_{0}{\bar{Y}}_{0\cdot 1}^{2}}\right]-\frac{{\bar{Y}}_{1\cdot 1}}{{\bar{Y}}_{0\cdot 1}}\right\}}^{2}=E{\left[r_{1}-E\left(r_{1}\right)\right]}^{2}=\mathrm{Var}\left(r_{1}\right).$$

Following the same steps, it can be shown that $\mathrm{Var}\left(r_{l}^{*}\right)=\mathrm{Var}\left(r_{l}\right)$ for l= 2, 3, 4, and 5 as well.

The distribution of ratios is not normal. For calculation of confidence intervals, we calculate $\ln\left(r_{l}^{*}\right)\forall l$. Using the delta method and the result above, $\mathrm{Var}\left[\ln\left(r_{l}^{*}\right)\right]=\mathrm{Var}\left(r_{l}\right)/{r_{l}^{*}}^{2}\forall l$, where $\mathrm{Var}\left(r_{l}\right)$ have been given in Equations (2), (3), (4), (8), and (13). Confidence intervals (CI) are calculated using the *t*‐distribution with $\;n_{1}+n_{0}-2$ degrees of freedom for non‐matched CRTs and n−1 degrees of freedom for matched‐paired CRTs.^14^ The CIs calculated are then exponentiated back to the original scale.

In Equation (4), the calculation of $\mathrm{Var}\left(r_{3}\right)$ involves an asymptotic variance estimator of $\mathrm{Var}\left(R_{i}\right)=\mathrm{Var}\left(\sum_{j=1}^{n_{i}}y_{\mathrm{ij}1}/\sum_{j=1}^{n_{i}}p_{\mathrm{ij}1}\right)$. Similarly, in Equations (8) and (13), this variance estimator is involved in the calculation of $\mathrm{Var}\left(R_{i}^{*}\right)$ and $\mathrm{Var}\left(R_{i}^{†}\right)$, and then the solutions are plugged into the estimators of $\mathrm{Var}\left(r_{4}\right)$ and $\mathrm{Var}\left(r_{5}\right)$, respectively. Cochran showed that this variance estimator gave a considerable under‐estimation.^7^ In contrast, he showed that the Jackknife method only mildly over‐estimated the variance and the over‐estimation vanished quickly as the number of observations increased. As such, an alternative method to statistical inference is to use the Jackknife method to estimate $\mathrm{Var}\left(R_{i}\right)$, $\mathrm{Var}\left(R_{i}^{*}\right),$ and $\mathrm{Var}\left(R_{i}^{†}\right)$ and then plug these values into the calculation of $\mathrm{Var}\left(\ln\left(r_{l}^{*}\right)\;\right)$, l= 3, 4, and 5 and the respective CIs. We will use $r_{l\left(J\right)}^{*}$, l= 3, 4, and 5, to denote the estimators when used together with this Jackknife‐based variance estimation method.

## 3 SIMULATION

### 3.1 Simulation setting

For non‐matched CRTs, we generated the number of events in the *k*th group in the *j*th cluster in the *i*th trial arm, conditional on the person‐time $p_{\mathrm{ijk}}$, by using a Poisson distribution with expected value given by Equation (1). We considered three sets of intervention effects, representing (a) direct protection (with no indirect effect), (b) direct and indirect protection, and (c) no effect, respectively: (a) $\exp\left(\beta_{D}\right)=0.5$ and $\exp\left(\beta_{I}\right)=1$; (b) $\exp\left(\beta_{D}\right)=0.5$ and $\exp\left(\beta_{I}\right)=0.75$; (c) $\exp\left(\beta_{D}\right)=1$ and $\exp\left(\beta_{I}\right)=1$. We set γ=1 for the difference in event rate between the target and non‐target groups. We set person‐times $\left(p_{\mathrm{ij}1},p_{\mathrm{ij}0}\right)$ as following a bivariate distribution with means 100, coefficient of variation $\mathrm{CV}\left(p_{\mathrm{ijk}}\right)=$ 0.2, 0.4, or 0.6, skewness 1.5, kurtosis 4, and correlation 0.8. Our choice of $\mathrm{CV}\left(p_{\mathrm{ijk}}\right)$ takes into account the findings from a recent systematic review of CRTs that the first quartile, median, and third quartile of $\mathrm{CV}\left(p_{\mathrm{ijk}}\right)$ were 0.22, 0.41, and 0.52, respectively.^15^ We used positively skewed distributions because that is implied by the sizeable CV(0.6) and positive values of person‐time. We used the *rmvnonnormal* macro in Stata for the non‐normal data generation.^16^ Additionally, we simulated person‐time using a bivariate normal distribution with means 100 and $\mathrm{CV}\left(p_{\mathrm{ijk}}\right)=$ 0.2 or 0.4, as symmetric distribution is possible under modest CV. A small number of observations (<1% in total) with person‐time either below 5 or above 350 were replaced by 5 or 350, respectively. This is because CRTs often exclude clusters that are very small in size and exclude or sub‐divide very large clusters due to operational and efficiency considerations.^5^, ^6^ We set the cluster effect, $\alpha_{\mathrm{ij}}$, as following a normal distribution with mean − 2 and $\mathrm{SD}\left(\alpha_{\mathrm{ij}}\right)=$ 0.05, 0.2, and 0.5. The three levels of $\mathrm{SD}\left(\alpha_{\mathrm{ij}}\right)$ correspond to approximately $\mathrm{CV}\left(c_{\mathrm{ij}}\right)$ = 0.05, 0.2, and 0.5.^5^ Hayes and Bennett noted that $\mathrm{CV}\left(c_{0j}\right)$ is “often ≤ 0.25 and seldom exceeds 0.5 for most health outcomes”.^14^

For matched‐pair CRTs, we generated the number of events in the *i*th trial arm in the *j*th paired cluster (j=1,2,…,n) by using a Poisson distribution with expected value $\exp\left(\beta_{D,\mathrm{ik}}+\beta_{I,i}+\alpha_{\mathrm{ij}}+\gamma \right)p_{\mathrm{ij}1}$ in the target group and $\exp\left(\beta_{I,i}+\alpha_{\mathrm{ij}}\right)p_{\mathrm{ij}0}$ in the non‐target group. We set the paired cluster effects $\left(\alpha_{1j},\alpha_{0j}\right)$ as following a bivariate normal distribution with means −2 and SDs 0.05, 0.2, or 0.5 and correlation 0.8. The person‐time parameter in the *k*th group in the *j*th pair of clusters $\left(p_{\mathrm{ijk}}\right)$ followed a multivariate non‐normal distribution with means 100, $\mathrm{CV}\left(p_{\mathrm{ijk}}\right)=$ 0.2, 0.4, or 0.6, skewness 1.5, kurtosis 4, and correlation 0.8. Additionally, multivariate normal distribution was used in the case of $\mathrm{CV}\left(p_{\mathrm{ijk}}\right)=$ 0.2 or 0.4. The other parameters in the matched‐pair CRTs were the same as those in the non‐matched CRTs.

In the literature, it has been suggested that non‐matched CRTs should include at least four clusters per trial arm and matched‐pair CRTs should include at least six pairs of clusters.^1^ For non‐matched CRTs, we evaluated the properties of the estimators when the number of clusters per trial arm is 4, 6, 8, 12, 16, 32, and 64. For matched‐pair CRTs, we considered 6, 8, 12, 16, 32, and 64 pairs of clusters.

In each scenario, we conducted 10 000 replicates of data generation and in each of them calculated the five asymptotically unbiased estimators and the five approximately unbiased estimators and their variances. We report the relative bias of the mean estimates of the incidence rate ratio, root mean square error (RMSE), coverage probability (CP) of the 95% confidence intervals (CI) and power to reject the null hyperthesis of the respective ratio equals one (or type 1 error when the null hyperthesis is true). Calculation of CIs was based on log‐transformation and then exponentiate back to the original scale, which were used for statistical inference. Calculations of relative bias and RMSE were based on the ratios themselves without transformation.

### 3.2 Simulation results

**FIGURE 1:**
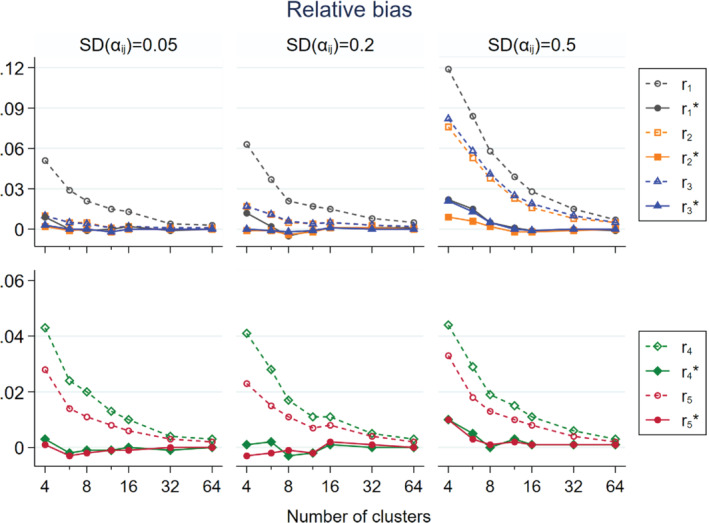
Relative bias of intervention effect estimators in relation to the number of clusters per trial arm for non‐matched CRTs, by three levels of $\mathrm{SD}\left(\alpha_{\mathrm{ij}}\right)$; population size per cluster follows a skewed distribution with mean = 100 and CV = 0.4; intervention has a direct effect only $\left(\exp\left(\beta_{D}\right)=0.5;\exp\left(\beta_{I}\right)=1\right)$

**FIGURE 2:**
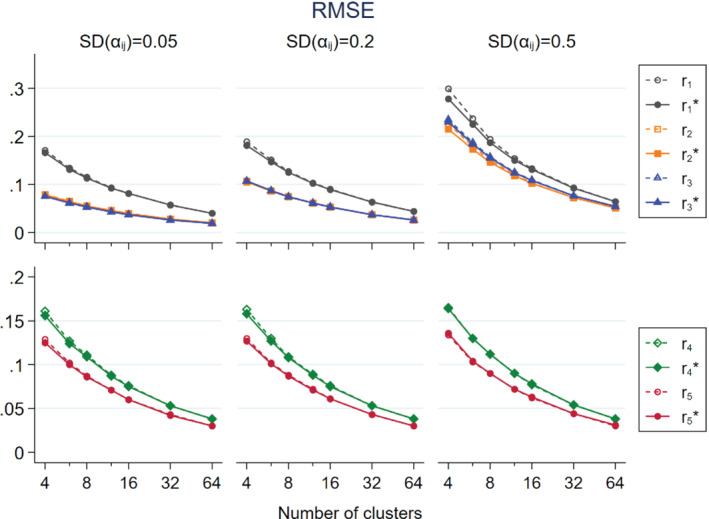
Root mean squared error (RMSE) of intervention effect estimators in relation to the number of clusters per trial arm for non‐matched CRTs, by three levels of $\mathrm{SD}\left(\alpha_{\mathrm{ij}}\right)$; population size per cluster follows a skewed distribution with mean = 100 and CV = 0.4; intervention has a direct effect only $\left(\exp\left(\beta_{D}\right)=0.5;\exp\left(\beta_{I}\right)=1\right)$

**FIGURE 3:**
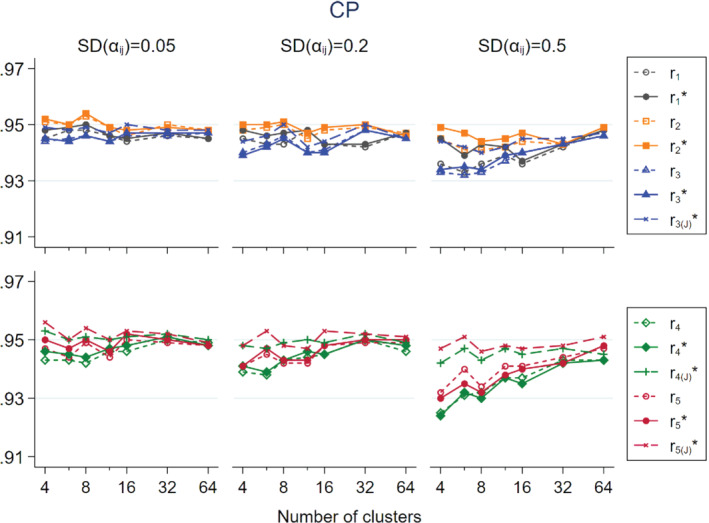
Coverage probability (CP) of 95% confidence interval (calculated on log‐scale and exponentiated back to the original scale) in relation to the number of clusters per trial arm for non‐matched CRTs, by three levels of $\mathrm{SD}\left(\alpha_{\mathrm{ij}}\right)$; population size per cluster follows a skewed distribution with mean = 100 and CV = 0.4; intervention has a direct effect only $\left(\exp\left(\beta_{D}\right)=0.5;\exp\left(\beta_{I}\right)=1\right)$

In Figures 1, 2, 3 we show the simulation results of non‐matched CRTs that the intervention only had a direct effect, that is, $\exp\left(\beta_{D}\right)=0.5$ and $\exp\left(\beta_{I}\right)=1$, and $\mathrm{CV}\left(p_{\mathrm{ij}}\right)=0.4$, which was approximately the median level of variability in cluster size found by a systematic review.^15^ To maintain visual clarity, we separately present the results on $r_{1}$, $r_{2}$, $r_{3}$, $r_{1}^{*}$, $r_{2}^{*}$, and $r_{3}^{*}$ (upper panel) and $r_{4}$, $r_{5}$, $r_{4}^{*}$, and $r_{5}^{*}$ (lower panel).

Figure 1 shows the patterns of relative bias. All the asymptotically unbiased estimators showed positive relative bias that decreased as the number of clusters per arm ($n_{i}$) increased. Furthermore, the bias of $r_{1}$, $r_{2}$, and $r_{3}$ increased as $\mathrm{SD}\left(\alpha_{\mathrm{ij}}\right)$ increased. In scenarios with $\mathrm{SD}\left(\alpha_{\mathrm{ij}}\right)\leq 0.2$, $r_{2}$, and $r_{3}$ only showed very mild bias and the two curves mostly overlapped. In contrast, the approximately unbiased estimators, $r_{1}^{*}$, $r_{2}^{*}$, and $r_{3}^{*}$, were practically unbiased under all situations considered. The relative bias of $r_{4}$ and $r_{5}$ was stable in relation to $\mathrm{SD}\left(\alpha_{\mathrm{ij}}\right)$. The approximately unbiased estimators $r_{4}^{*}$ and $r_{5}^{*}$ were practically unbiased under all situations considered.

In Figure 2, $r_{2}^{*}$ and $r_{3}^{*}$ had smaller RMSE than $r_{1}^{*}$, but they converged as $n_{i}$ increased. $r_{2}^{*}$ had slightly smaller RMSE than $r_{3}^{*}$ when $\mathrm{SD}\left(\alpha_{\mathrm{ij}}\right)=0.5$ and $n_{i}$ was small. Otherwise they were almost indistinguishable from each other. $r_{5}^{*}$ had smaller RMSE than $r_{4}^{*}$. Their difference was stable across level of $\mathrm{SD}\left(\alpha_{\mathrm{ij}}\right)$ but they converged as $n_{i}$ increased. The asymptotically unbiased estimators had RMSE similar to or slightly larger than their respective approximately unbiased counterparts', but the difference vanished as $n_{i}$ increased.

The estimators tended to have coverage probability (CP) smaller than the nominal 95% level, especially when $\mathrm{SD}\left(\alpha_{\mathrm{ij}}\right)$ was large (Figure 3). The CP improved as $n_{i}$ increased. The asymptotically unbiased estimators tended to have similar or slightly lower CP than their respective approximately unbiased counterparts. In the upper panel, $r_{2}^{*}$ had CP close to the nominal 95% across all levels of $n_{i}$ and $\mathrm{SD}\left(\alpha_{\mathrm{ij}}\right)$. $r_{1}^{*}$ and $r_{3}^{*}$ had CP below 94% in some scenarios of small $n_{i}$ and large $\mathrm{SD}\left(\alpha_{\mathrm{ij}}\right)$. Using the Jackknife‐based variance estimator described in Section 2 for $r_{3}^{*}$, denoted by $r_{3\left(J\right)}^{*}$, gave improved CP that was closer to the nominal 95% level than $r_{3}^{*}$. In the lower panel, $r_{4}$, $r_{5}$, $r_{4}^{*}$, and $r_{5}^{*}$ had varying degree of under‐coverage in different scenarios. However, using the Jackknife‐based variance estimators for $r_{4}^{*}$ and $r_{5}^{*}$, denoted by $r_{4\left(J\right)}^{*}$ and $r_{5\left(J\right)}^{*}$, respectively, the CP was close to the nominal level in all situations.

**FIGURE 4:**
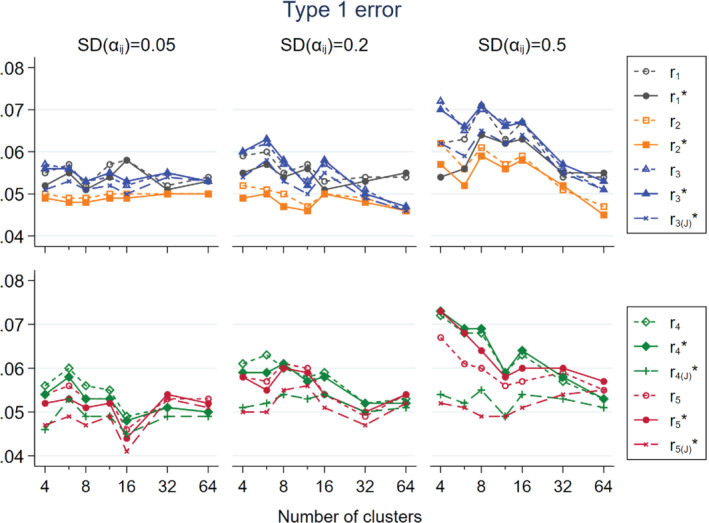
Type 1 error rate in relation to the number of clusters per trial arm for non‐matched CRTs, by three levels of $\mathrm{SD}\left(\alpha_{\mathrm{ij}}\right)$; population size per cluster follows a skewed distribution with mean = 100 and CV = 0.4; intervention has no effect $\left(\exp\left(\beta_{D}\right)=1;\exp\left(\beta_{I}\right)=1\right)$

Figure 4 shows type 1 error rates, that is, rejection of null hypothesis in scenarios with $\exp\left(\beta_{D}\right)=1$ and $\exp\left(\beta_{I}\right)=1$. Otherwise, the parameters are the same as those in Figures 1, 2, 3. There was a tendency for all estimators to have type 1 error rate that exceeded the 5% target level, especially with $\mathrm{SD}\left(\alpha_{\mathrm{ij}}\right)\geq$ 0.2 and $n_{i}<$ 32. The inflation reduced as $n_{i}$ increased. In the upper panel, $r_{2}$ and $r_{2}^{*}$, followed by $r_{3\left(J\right)}^{*}$ and $r_{1}^{*}$, performed better than the others. In the lower panel, $r_{4}$, $r_{5}$, $r_{4}^{*}$, and $r_{5}^{*}$ had varying level of inflation of type 1 error under different parameter settings. In contrast, $r_{4\left(J\right)}^{*}$ and $r_{5\left(J\right)}^{*}$ performed well. In no circumstances did they show more than 1% deviation from the 5% target.

**FIGURE 5:**
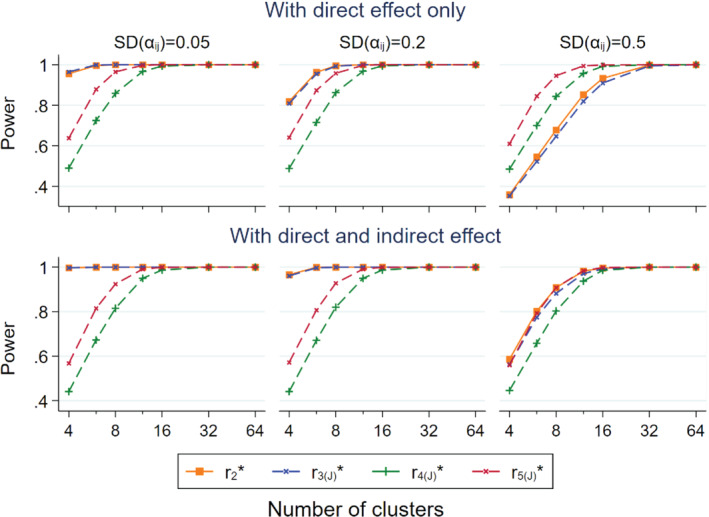
Power of $r_{2}^{*},r_{3\left(J\right)}^{*},r_{4\left(J\right)}^{*},\text{and}\;r_{5\left(J\right)}^{*}$ in relation to the number of clusters per trial arm for non‐matched CRTs, by three levels of $\mathrm{SD}\left(\alpha_{\mathrm{ij}}\right)$; population size per cluster follows a skewed distribution with mean = 100 and CV = 0.4. Upper panel: with direct effect only $\left(\exp\left(\beta_{D}\right)=0.5;\exp\left(\beta_{I}\right)=1\right)$; lower panel: with direct and indirect effects $\left(\exp\left(\beta_{D}\right)=0.5;\exp\left(\beta_{I}\right)=0.75\right)$

Figure 5 compares the power of selected estimators that use person‐time as denominators, $r_{2}^{*}$ and $r_{3\left(J\right)}^{*}$, vs selected estimators that use event counts in the non‐target group as denominators, $r_{4\left(J\right)}^{*}$ and $r_{5\left(J\right)}^{*}$. We focused on them because they performed well in terms of CP and type 1 error rate. The lower panel introduced an indirect effect, $\exp\left(\beta_{I}\right)=0.75$, in addition to a direct effect, $\exp\left(\beta_{D}\right)=0.5$. Otherwise, the parameters here are the same as those in Figures 1, 2, 3, 4. When there was direct effect only (upper panel), $r_{2}^{*}$ and $r_{3\left(J\right)}^{*}$ were more powerful than $r_{4\left(J\right)}^{*}$ and $r_{5\left(J\right)}^{*}$ for $\mathrm{SD}\left(\alpha_{\mathrm{ij}}\right)\leq 0.2$. In contrast, $r_{4\left(J\right)}^{*}$ and $r_{5\left(J\right)}^{*}$ were more powerful when $\mathrm{SD}\left(\alpha_{\mathrm{ij}}\right)=0.5$. Furthermore, $r_{5\left(J\right)}^{*}$ was more powerful than $r_{4\left(J\right)}^{*}$ in all the scenarios considered. With the addition of the indirect effect, $r_{2}^{*}$ and $r_{3\left(J\right)}^{*}$ were more powerful than $r_{4\left(J\right)}^{*}$ at all levels of $\mathrm{SD}\left(\alpha_{\mathrm{ij}}\right)$, but similar to $r_{5\left(J\right)}^{*}$ at $\mathrm{SD}\left(\alpha_{\mathrm{ij}}\right)=0.5$.

Further simulation results on non‐matched CRTs under other parameter settings and simulation results on matched‐pair CRTs are available in Online Supplementary Material 2. The findings are qualitatively similar to those reported above. Some relatively important additional information is as follows: First, in non‐matched CRTs, the relative bias of $r_{1}$ and $r_{4}$ increased substantially as $\mathrm{CV}\left(p_{\mathrm{ij}1}\right)$ increased except when the number of cluster was about 32 or above. In contrast, $r_{2}$, $r_{3}$, $r_{5}$ and the approximately unbiased estimators were much less sensitive to the magnitude of $\mathrm{CV}\left(p_{\mathrm{ij}1}\right)$ (eg, Figures S1 and S2). Second, in non‐matched CRTs, the CP of $r_{3\left(J\right)}^{*}$ reduced to about 93% and its type 1 error rate increased to about 7% when $\mathrm{CV}\left(p_{\mathrm{ij}1}\right)$ = 0.6, $\mathrm{SD}\left(\alpha_{\mathrm{ij}}\right)$ = 0.5 and the number of clusters was 6 or below (eg, Figures S6 and S26). However, in matched‐paired CRTs, $r_{3\left(J\right)}^{*,\text{paired}}$ performed well in these aspects while $r_{2}^{*,\text{paired}}$ had somewhat inflated type 1 error rate and below target CP (eg, Figures S41, S42, S62, and S63).

## 4 SEASONAL MALARIA CHEMOPREVENTION TRIAL

**FIGURE 6:**
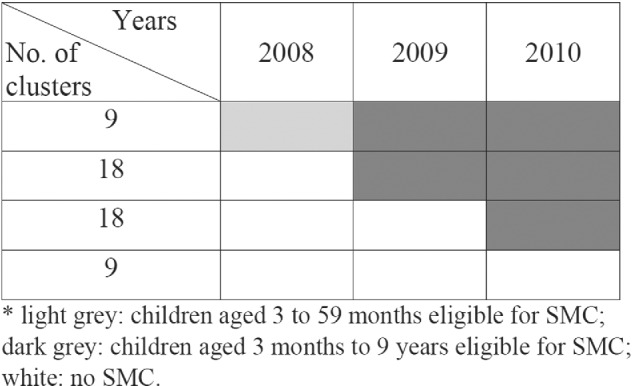
Design of seasonal malaria chemoprevention (SMC) trial

We use a subset of data from a published study of seasonal malaria chemoprevention (SMC) in Senegalese children to illustrate.^17^ The trial set‐up is shown in Figure 6. The trial had a total of 54 clusters. It had the appearance of an “optimal design”,^18^, ^19^ with nine clusters on intervention (leftmost column) and nine clusters on control condition (rightmost column) for all three time periods (2008 to 2010) that resembled a non‐matched CRT, flanking a standard stepped‐wedge trial (middle columns). The middle columns represent 18 clusters that were randomized to receive SMC from 2009 and another 18 clusters randomized to receive SMC from 2010. However, the trial was not planned according to the optimal design. The original plan was that the trial would continue up to 4 years (2008 to 2011), and the nine clusters on the rightmost column were randomized to receive SMC in 2011. But the trial was terminated after the malaria transmission season in 2010 according to data monitoring and interim analysis results. Furthermore, in the first period (2008), children aged 3 to 59 months in the nine clusters on the leftmost column were given SMC as part of the preparation of the study logistics. In 2009 and 2010, children aged between 3 months and 9 years (inclusive) were given SMC.

A passive surveillance system was implemented in health facilities to determine the number of clinical malaria episodes confirmed by rapid malaria test (primary endpoint) in each cluster, by four age groups (59 months or below; 5‐9; 10‐19; over 19 years). The data was at the cluster, not individual, level. Mortality data was collected for all age groups but only data for children aged 9 years or below was used in the previously published analysis^17^ and available to the present analysis. Number of deaths (secondary endpoint) and population size of the clusters at mid‐September each year (approximately the beginning of the annual malaria transmission season), by age groups, was collected by a demographic surveillance system.

For the purpose of illustration, we used the 2008 data from the 18 clusters that resembled a non‐matched CRT. Children aged between 3 to 59 months were the target group. We considered children aged 5 to 9 years the non‐target group.

Table 1 shows the descriptive statistics by endpoints and trial arms. It also included a simple average of each statistics in the two trial arms as a summary. There was very large between‐cluster variability in malaria incidence rate in the target group, with $\mathrm{CV}\left(c_{\mathrm{ij}1}\right)$>1 in both arms. In the target group, the CVs of malaria episodes approximately doubled the CVs of the population size, with $\theta =\mathrm{CV}\left(p_{\mathrm{ij}1}\right)/\mathrm{CV}\left(y_{\mathrm{ij}1}\right)\approx 0.60/1.37=0.44$. The correlation coefficient between malaria episodes and population size of the target group, $\text{corr}\left(y_{\mathrm{ij}1},p_{\mathrm{ij}1}\right)$, was weak, with average across trial arms being only 0.03. The estimates of malaria incidence rate in the SMC and control arms were ${\bar{c}}_{1}=0.0029>R_{1}=0.0028$ and ${\bar{c}}_{0}=0.0031>R_{0}=0.0020$, respectively. In contrast, the correlation between malaria episodes in the target and non‐target group, $\text{corr}\left(y_{\mathrm{ij}1},y_{\mathrm{ij}0}\right)$, was strong, averaged at 0.76.

**TABLE 1:** Descriptive summary of number of malaria episodes and deaths, population size and their correlations and coefficient of variation of cluster‐level event rates in 9 intervention clusters and 9 control clusters in a seasonal malaria chemoprevention (SMC) trial in 2008

	Malaria			Mortality		
Statistics	SMC	Control	Average	SMC	Control	Average
${\bar{y}}_{\mathrm{ij}1}$	8.00	3.56	5.78	7.11	2.89	5.00
${\bar{y}}_{\mathrm{ij}0}$	15.44	5.89	10.67	1.00	0.56	0.78
${\bar{p}}_{\mathrm{ij}1}$	2834	1741	2288	2834	1741	2288
${\bar{p}}_{\mathrm{ij}0}$	1977	1187	1582	1977	1187	1582
$\mathrm{CV}\left(c_{\mathrm{ij}1}\right)$	1.21	1.75	1.48	0.89	0.68	0.79
$\mathrm{CV}\left(y_{\mathrm{ij}1}\right)$	1.24	1.49	1.37	1.05	0.95	1.00
$\mathrm{CV}\left(y_{\mathrm{ij}0}\right)$	1.15	1.08	1.12	1.58	1.31	1.45
$\mathrm{CV}\left(p_{\mathrm{ij}1}\right)$	0.46	0.73	0.60	0.46	0.73	0.60
$\mathrm{CV}\left(p_{\mathrm{ij}0}\right)$	0.49	0.65	0.57	0.49	0.65	0.57
$\text{corr}\left(y_{\mathrm{ij}1},p_{\mathrm{ij}1}\right)$	0.10	−0.05	0.03	0.48	0.69	0.59
$\text{corr}\left(y_{\mathrm{ij}1},p_{\mathrm{ij}0}\right)$	0.13	−0.05	0.04	0.57	0.68	0.63
$\text{corr}\left(y_{\mathrm{ij}0},p_{\mathrm{ij}1}\right)$	0.26	0.05	0.15	0.18	0.58	0.38
$\text{corr}\left(y_{\mathrm{ij}0},p_{\mathrm{ij}0}\right)$	0.34	0.01	0.17	0.26	0.57	0.41
$\text{corr}\left(y_{\mathrm{ij}1},y_{\mathrm{ij}0}\right)$	0.88	0.64	0.76	0.67	0.35	0.51
$\text{corr}\left(p_{\mathrm{ij}1},p_{\mathrm{ij}0}\right)$	0.988	0.998	0.993	0.988	0.998	0.993

Table 2 shows the estimation results. The estimates based on the asymptotically unbiased estimators were all larger than those based on the approximately unbiased estimators. Given this number of clusters, we considered the latter more accurate. For *l* = 3, 4, and 5, the *SE*'s based on $r_{l\left(J\right)}^{*}$ were only slightly larger than $r_{l}^{*}.$ All confidence intervals were quite wide. Although $\text{corr}\left(y_{\mathrm{ij}1},p_{\mathrm{ij}1}\right)$ was weak, the estimated $r_{1}$ was much larger than $r_{2}$ and $r_{3}$, leading to standard error $\mathrm{SE}\left(r_{1}\right)$ larger than $\mathrm{SE}\left(r_{2}\right)$ and $\mathrm{SE}\left(r_{3}\right)$. Given ${\bar{c}}_{i}>R_{i}$ in both trial arms, $\mathrm{SE}\left(r_{2}\right)<\mathrm{SE}\left(r_{3}\right)$. As expected from Equations (9) and (10), with strong $\text{corr}\left(y_{\mathrm{ij}1},y_{\mathrm{ij}0}\right)$ and weak $\text{corr}\left(y_{\mathrm{ij}1},p_{\mathrm{ij}1}\right)$, $\mathrm{SE}\left(r_{4}\right)$ was smaller than $\mathrm{SE}\left(r_{2}\right)$ and $\mathrm{SE}\left(r_{3}\right)$. The estimators for direct effects, $r_{4}^{*}$ and $r_{5}^{*}$, gave very similar result. Since there was no practical difference between $\text{corr}\left(y_{\mathrm{ij}1},p_{\mathrm{ij}1}\right)+\text{corr}\left(y_{\mathrm{ij}0},p_{\mathrm{ij}0}\right)$ and $\text{corr}\left(y_{\mathrm{ij}1},p_{\mathrm{ij}0}\right)+\text{corr}\left(y_{\mathrm{ij}0},p_{\mathrm{ij}1}\right)$ in either trial arm, as indicated by Equation (14), $\mathrm{Var}\left(r_{4}\right)≅\mathrm{Var}\left(r_{5}\right)$.

**TABLE 2:** Estimates, SE and 95% confidence intervals (CI; exponentiation of log‐transformed values) for malaria and mortality in seasonal malaria chemoprevention trial data in 2008

Endpoint	Estimator	Estimate	*SE*	95% CI
Malaria	$r_{1}^{*}$	1.70	1.45	(0.28, 10.4)
	$r_{2}^{*}$	0.62	0.67	(0.06, 6.07)
	$r_{3}^{*}$	1.01	0.97	(0.13, 7.80)
	$r_{3\left(J\right)}^{*}$	1.01	1.00	(0.12, 8.26)
	$r_{4}^{*}$	0.74	0.37	(0.25, 2.14)
	$r_{4\left(J\right)}^{*}$	0.74	0.40	(0.23, 2.32)
	$r_{5}^{*}$	0.76	0.38	(0.26, 2.19)
	$r_{5\left(J\right)}^{*}$	0.76	0.41	(0.24, 2.38)
	$r_{1}$	2.25	1.45	(0.57, 8.85)
	$r_{2}$	0.94	0.67	(0.21, 4.24)
	$r_{3}$	1.38	0.97	(0.31, 6.16)
	$r_{4}$	0.86	0.37	(0.34, 2.14)
	$r_{5}$	0.88	0.38	(0.35, 2.20)
Mortality	$r_{1}^{*}$	2.21	1.16	(0.73, 6.74)
	$r_{2}^{*}$	1.16	0.46	(0.50, 2.67)
	$r_{3}^{*}$	1.44	0.58	(0.61, 3.40)
	$r_{3\left(J\right)}^{*}$	1.44	0.61	(0.59, 3.54)
	$r_{4}^{*}$	1.08	0.81	(0.22, 5.26)
	$r_{4\left(J\right)}^{*}$	1.08	0.88	(0.19, 6.02)
	$r_{5}^{*}$	1.12	0.83	(0.23, 5.41)
	$r_{5\left(J\right)}^{*}$	1.12	0.91	(0.20, 6.33)
	$r_{1}$	2.46	1.16	(0.90, 6.70)
	$r_{2}$	1.22	0.46	(0.55, 2.70)
	$r_{3}$	1.51	0.58	(0.67, 3.42)
	$r_{4}$	1.37	0.81	(0.39, 4.78)
	$r_{5}$	1.40	0.83	(0.40, 4.92)

Table 1 also shows that the descriptive statistics on mortality. There was less between‐cluster variability in mortality rate than malaria incidence, with $\mathrm{CV}\left(c_{\mathrm{ij}1}\right)$ in the two arms averaged at 0.79. Furthermore, in the target group, the CVs of death approximately doubled the CVs of the population size, with $\theta =\mathrm{CV}\left(p_{\mathrm{ij}1}\right)/\mathrm{CV}\left(y_{\mathrm{ij}1}\right)\approx 0.60/1.00=0.60$. Unlike malaria episodes, the correlation coefficient between deaths and population size of the target group was more substantial, with average across trial arms being 0.59. The estimates of mortality rate in the SMC and control arms were ${\bar{c}}_{1}=0.0022<R_{1}=0.0025$ and ${\bar{c}}_{0}=0.0018>R_{0}=0.0017$, respectively. The correlation between deaths in the target and non‐target group was moderate, with average $\text{corr}\left(y_{\mathrm{ij}1},y_{\mathrm{ij}0}\right)$ at 0.51; this was weaker than that for malaria episodes.

Estimation results on mortality are available in Table 2. Again, the asymptotically unbiased estimators tended to give larger estimates than their respective approximately unbiased estimators. However, the differences between the two set of estimates were smaller than those on malaria episodes, as expected from the smaller between‐cluster variability in mortality rate than malaria incidence rate. The *SE*'s based on $r_{l\left(J\right)}^{*}$ were only slightly larger than those based on $r_{l}^{*}.$ All confidence intervals were quite wide. Since $\text{corr}\left(y_{\mathrm{ij}1},p_{\mathrm{ij}1}\right)$ was substantial and the estimated $r_{1}$ was much larger than $r_{2}$ and $r_{3}$, $\mathrm{SE}\left(r_{2}\right)$ and $\mathrm{SE}\left(r_{3}\right)$ were smaller than $\mathrm{SE}\left(r_{1}\right)$. With fairly similar values of $\text{corr}\left(y_{\mathrm{ij}1},y_{\mathrm{ij}0}\right)$ and $\text{corr}\left(y_{\mathrm{ij}1},p_{\mathrm{ij}1}\right)$ and relatively large θ, $\mathrm{SE}\left(r_{2}\right)$ and $\mathrm{SE}\left(r_{3}\right)$ were smaller than $\mathrm{SE}\left(r_{4}\right)$. There was no clear difference between $\text{corr}\left(y_{\mathrm{ij}1},p_{\mathrm{ij}1}\right)+\text{corr}\left(y_{\mathrm{ij}0},p_{\mathrm{ij}0}\right)$ and $\text{corr}\left(y_{\mathrm{ij}1},p_{\mathrm{ij}0}\right)+\text{corr}\left(y_{\mathrm{ij}0},p_{\mathrm{ij}1}\right)$ in either trial arm, so $\mathrm{Var}\left(r_{4}\right)≅\mathrm{Var}\left(r_{5}\right)$.

## 5 DISCUSSION

The proposed approximately unbiased estimators successfully reduce the bias when the number of clusters is small. They also have advantages in terms of smaller RMSE and more accurate coverage probability than the asymptotically unbiased estimators. For studies with fewer than 60 clusters per arm, we recommend the use of the approximately unbiased estimators. Some CRTs do have a large number of clusters per trial arm, for example, a trial of influenza vaccination had over 400 nursing homes per arm^20^ and a trial of mass drug administration had over 700 communities per arm.^21^ For such studies, the choice between the asymptotically and approximately unbiased estimators is unimportant. Furthermore, with a large number of clusters, the simple estimator $r_{1}$ has performance very similar to $r_{2}$ and $r_{3}$. At the study planning stage, investigators may take into account this finding when they consider the cost and benefit of collecting person‐time data.

Previous simulation studies evaluated the performance of the estimator $r_{2}$.^5^, ^6^ We caution that the range of parameter values they considered were somewhat narrow. From our analytic solution, the estimator is only asymptotically unbiased. As seen in Equation (15), the bias in the estimator $r_{2}$ is a non‐linear function of the between‐cluster variability of event rate. From simulation, the bias of $r_{2}$ became obvious as the variability increased, especially when the number of clusters per trial arm was below 16 or so. In the SMC study of malaria episodes, where the variability was large, the $r_{2}$ estimate was much larger than $r_{2}^{*}$. In those situations, the use of our proposed bias‐corrected estimator $r_{2}^{*}$ is preferable over $r_{2}$.

While $r_{3}^{*}$ performs well in terms of bias and RMSE, its variance estimator under‐estimates the true variability as the CV of cluster size or event rate increases; it approaches the target CP as the number of clusters increases. The under‐coverage can be corrected by plugging the Jackknife estimates of $\mathrm{Var}\left(R_{i}\right)$ into the estimator for $\mathrm{Var}\left(r_{3}\right)$. We found that $r_{2}^{*}$ and $r_{3\left(J\right)}^{*}$ have similar performance except when $\mathrm{CV}\left(p_{\mathrm{ijk}}\right)$ = 0.6 or $\mathrm{SD}\left(\alpha_{\mathrm{ij}}\right)$ = 0.5. In those settings, $r_{3\left(J\right)}^{*}$ had type 1 error rate up to about 2% higher than the nominal 5% level when the number of clusters was six or below, and $r_{2}^{*}$ may be preferred. However, in matched‐pair CRTs, $r_{2}^{*,\text{paired}}$ did not out‐perform $r_{3\left(J\right)}^{*,\text{paired}}$. Furthermore, previous studies had shown that the estimator of cluster‐level event rate in a trial arm, ${\bar{c}}_{i}$, is a biased estimator, and the level of bias does not reduce in relation to increase in number of clusters.^8^ In contrast, $R_{i}$ can be used to obtain an asymptotically unbiased estimate of disease incidence.^7^, ^8^ Even if $r_{2}^{*}$ is used to estimate incidence rate ratio, $R_{i}$ is preferable over ${\bar{c}}_{i}$ as an estimator of the incidence rate in each trial arm.

Our consideration of $r_{4}$ and $r_{5}$ was in part motivated by an evaluation of a malaria vaccine where indirect effects were unlikely and the incidence of some outcomes such as meningitis (a safety outcome) could be highly variable between clusters, and where balanced randomization with respect to access to health care facilities (where passive surveillance took place) was difficult to ensure.^22^ When $\mathrm{SD}\left(\alpha_{\mathrm{ij}}\right)$ is large, the number of events in the target group is likely to be more strongly correlated with the number of events in the non‐target group than with the person‐time in the target group. Furthermore, it is typical that vaccine studies anticipate no indirect effect on safety endpoints. So, these estimators are expected to estimate the same target as far as safety is concerned. In simulation studies of scenarios that the intervention only had a direct effect, we have seen that when $\mathrm{SD}\left(\alpha_{\mathrm{ij}}\right)$ was ≤0.2, $r_{2}^{*}$ and $r_{3\left(J\right)}^{*}$ tended to be more powerful than $r_{4\left(J\right)}^{*}$ and $r_{5\left(J\right)}^{*}$. When $\mathrm{SD}\left(\alpha_{\mathrm{ij}}\right)$ increased to 0.5 and in the malaria data in the SMC trial, $r_{4\left(J\right)}^{*}$ and $r_{5\left(J\right)}^{*}$ had substantially smaller SE than $r_{2}^{*}$ and $r_{3\left(J\right)}^{*}$. For situations like this, $r_{4\left(J\right)}^{*}$ and $r_{5\left(J\right)}^{*}$ are our estimators of choice. In various scenarios we evaluated, $r_{5\left(J\right)}^{*}$ gave higher level of statistical power than $r_{4\left(J\right)}^{*}$, except that they had similar performance when $\mathrm{CV}\left(p_{\mathrm{ijk}}\right)$ was small. In that case, the magnitude of the benefits may not justify the extra cost in collection of person‐time data in both the target and non‐target groups. Otherwise, $r_{5\left(J\right)}^{*}$ tends to be preferable over $r_{4\left(J\right)}^{*}$.

We foresee that the estimators that use person‐time in the target group and estimators that use number of events in the non‐target groups as a denominator may be used in different parts of the same CRT, depending on the considerations aforementioned. For example, $r_{2}$ or $r_{3}$ or their extensions may be used in efficacy analysis while $r_{4}$ and $r_{5}$ or their extensions may be used in safety analysis.

Furthermore, $r_{4}$ and $r_{5}$ are generic quantities in the sense that $y_{\mathrm{ij}0}$ may be replaced by quantities other than the number of events in non‐target groups to achieve other purposes. For example, there has been interest in the use of “negative control events” to remove the bias arising from differential ascertainment of outcome events in non‐blinded CRTs.^4^ The proposed estimator in the literature is in the form of $r_{4}$, with $y_{1j0}$ and $y_{0j0}$ replaced by the number of negative control events in the intervention and control arm, respectively.^4^ The previous study did not consider the properties of the estimator in situations with small number of clusters. The results here apply directly. Another example of application is to the CRT with Before and after observations (CRT‐BA) design,^23^ which collects data in a baseline period before launching the randomized intervention and control comparator. By replacing $y_{\mathrm{ij}0}$ by the baseline event count, $r_{4}$ becomes a baseline adjusted estimator of the total effect. It offers a robust alternative to the analysis of CRT‐BA trials.

The strengths of the present study include coverage of both non‐matched and matched‐pair CRTs, analytic evaluation of the bias of existing asymptotically unbiased estimators, proposal of estimators that capitalize on denominators other than cluster population size and their potential applications, development of a bias‐corrected version of these estimators for use in studies with a small number of clusters, and simulation evaluation of the estimators with a realistic range of variability in cluster size. A limitation is that the methods do not handle covariate adjustment. However, good use of restricted randomization may reduce the need for covariate adjustment in the analysis stage.^1^

In non‐matched CRTs, one approach for controlling covariate effects is stratified analysis and pooling of stratum‐specific estimates using weights inversely proportional to variances. The limitation is that it is not practical to stratify for multiple covariates and it requires categorization of continuous covariates. Another approach is to apply ANCOVA to cluster‐level data. However, it only works for methods that generate a summary value per cluster, such as $r_{1}$ and $r_{2}$, whose calculation begins with getting an event count and event rate per cluster, respectively. This is different from the calculation of $r_{3}$ to $r_{5}$, which begins with generating an estimate for a trial arm. For example, in $r_{3}$, the incidence rate in a trial arm is the sum of events over clusters divided by the sum of person‐time over clusters within the trial arm. There is not a summary value for every cluster. Furthermore, when the purpose is to estimate rate ratio instead of rate difference, the ANCOVA approach would need to analyze the log‐transformed values instead. The exponentiated intervention effect estimate is then interpreted as a ratio of geometric means, which is not the same as the widely used estimator of ratio of arithmetic means (including $r_{1}$ and $r_{2}$). In CRTs with a small number of clusters, the bias and bias‐correction for ratios of geometric means of event counts and event rates have yet to be investigated. Another approach is to use Poisson regression analysis with cluster‐level covariates (and individual‐level covariates if available) without using the intervention variable as predictors to obtain an expected number of events for each cluster.^5^ Comparison of the deviations of the observed from the expected number of events between trial arms then offers a covariate adjusted estimate for the intervention effect. Following this idea, if $y_{\mathrm{ij}0}$ in $r_{4}$ or $p_{\mathrm{ijk}}$ in $r_{5}$ are replaced by this expected number of events, they become covariate adjusted estimators of the total or direct effect, respectively. The bias‐correction method may then be applied to obtain covariate adjusted $r_{4}^{*}$ or $r_{5}^{*}$. But the variance estimators may not work well as they do not account for the uncertainty in the prediction of the expected number of events. In short, while there are several candidate approaches available, challenges remain. Further research is needed to develop and evaluate these or other approaches to covariate adjustment.

The size of $\text{corr}\left(y_{\mathrm{ij}1},p_{\mathrm{ij}1}\right)$ is important in determining the relative strength of the different estimators. A large $\mathrm{SD}\left(\alpha_{\mathrm{ij}}\right)$ tends to dilute this correlation. As such, pilot data and careful consideration of the between‐cluster variability in event rate is important not only for sample size determination but also for choice of study design and statistical analysis procedures.

## CONFLICT OF INTEREST

The authors declare no potential conflicts of interest with respect to the research, authorship, and/or publication of this article.

## Supporting information


**Online Supplementary Material 1**: Comparisons of asymptotically unbiased estimators and derivations of approximately unbiased estimators

**Online Supplementary Material 2**: Further simulation results, by number of clusters per trial arm and three levels of SD (*α*_*ij*_); mean population size per cluster is 100

## Data Availability

The data used in the case study are available at https://doi.org/10.17037/DATA.117. Requests for access will be reviewed by a Data Access Committee to ensure use of the data protect participant privacy according to the terms of participant consent and ethics committee approval. Stata codes for the simulations will be deposited to figshare by Wiley.

## APPENDIX A.

**TABLE A1:** Estimators of incidence rate ratio for matched‐pair cluster randomized trials^a^

Label	Estimand ^b^	Estimator	Variance ^c^
Ratio of means	$\exp\left(\beta_{T}\right)$	$r_{1}^{\text{paired}}=\frac{{\bar{y}}_{1}}{{\bar{y}}_{0}}=\frac{\sum_{j=1}^{n}y_{1j1}}{\sum_{j=1}^{n}y_{0j1}}$	$\frac{{\bar{y}}_{1}^{2}}{n{\bar{y}}_{0}^{2}}\left\{\frac{\mathrm{Var}\left(y_{1j1}\right)}{{\bar{y}}_{1}^{2}}+\frac{\mathrm{Var}\left(y_{0j1}\right)}{{\bar{y}}_{0}^{2}}-\frac{2\mathrm{cov}\left(y_{1j1},y_{0j1}\right)}{{\bar{y}}_{1}{\bar{y}}_{0}}\right\}$
Ratio of mean cluster‐level event rates	$\exp\left(\beta_{T}\right)$	$r_{2}^{\text{paired}}=\frac{{\bar{c}}_{1}}{{\bar{c}}_{0}}=\frac{\sum_{j=1}^{n}c_{1j}}{\sum_{j=1}^{n}c_{0j}}$	$\frac{{\bar{c}}_{1}^{2}}{n{\bar{c}}_{0}^{2}}\left\{\frac{\mathrm{Var}\left(c_{1j}\right)}{{\bar{c}}_{1}^{2}}+\frac{\mathrm{Var}\left(c_{0j}\right)}{{\bar{c}}_{0}^{2}}-\frac{2\mathrm{cov}\left(c_{1j},c_{0j}\right)}{{\bar{c}}_{1}{\bar{c}}_{0}}\right\}$
Ratio of event rates	$\exp\left(\beta_{T}\right)$	$r_{3}^{\text{paired}}=\frac{R_{1}}{R_{0}}=\frac{\sum_{j=1}^{n}y_{1j1}/\sum_{j=1}^{n}p_{1j1}}{\sum_{j=1}^{n}y_{0j1}/\sum_{j=1}^{n}p_{0j1}}$	${\left(\frac{R_{1}}{R_{0}}\right)}^{2}\left(\frac{\mathrm{Var}\left(R_{1}\right)}{R_{1}^{2}}+\frac{\mathrm{Var}\left(R_{0}\right)}{R_{0}^{2}}-\frac{2\mathrm{cov}\left(R_{1},R_{0}\right)}{R_{1}R_{0}}\right)$
Double ratio of counts	$\exp\left(\beta_{D}\right)$	$r_{4}^{\text{paired}}=\frac{R_{1}^{*}}{R_{0}^{*}}=\frac{\sum_{j=1}^{n}y_{1j1}/\sum_{j=1}^{n}y_{1j0}}{\sum_{j=1}^{n}y_{0j1}/\sum_{j=1}^{n}y_{0j0}}$	${\left(\frac{R_{1}^{*}}{R_{0}^{*}}\right)}^{2}\left(\frac{\mathrm{Var}\left(R_{1}^{*}\right)}{R_{1}^{*2}}+\frac{\mathrm{Var}\left(R_{0}^{*}\right)}{R_{0}^{*2}}-\frac{2\mathrm{cov}\left(R_{1}^{*},R_{0}^{*}\right)}{R_{1}^{*}R_{0}^{*}}\right)$
Double ratio of event rates	$\exp\left(\beta_{D}\right)$	$r_{5}^{\text{paired}}=\frac{R_{1}^{†}}{R_{0}^{†}}=\frac{\left(\frac{\sum_{j=1}^{n}y_{1j1}}{\sum_{j=1}^{n}p_{1j1}}\right)/\left(\frac{\sum_{j=1}^{n}y_{1j0}}{\sum_{j=1}^{n}p_{1j0}}\right)}{\left(\frac{\sum_{j=1}^{n}y_{0j1}}{\sum_{j=1}^{n}p_{0j1}}\right)/\left(\frac{\sum_{j=1}^{n}y_{0j0}}{\sum_{j=1}^{n}p_{0j0}}\right)}$	${\left(\frac{R_{1}^{†}}{R_{0}^{†}}\right)}^{2}\left(\frac{\mathrm{Var}\left(R_{1}^{†}\right)}{R_{1}^{†2}}+\frac{\mathrm{Var}\left(R_{0}^{†}\right)}{R_{0}^{†2}}-\frac{2\mathrm{cov}\left(R_{1}^{†},R_{0}^{†}\right)}{R_{1}^{†}R_{0}^{†}}\right)$

^a^ $y_{\mathrm{ijk}},\;p_{\mathrm{ijk}}$, and $c_{\mathrm{ij}}=y_{\mathrm{ij}1}/p_{\mathrm{ij}1}$ are, respectively, the number of events, person‐time/population size and cluster‐level event rates in the cluster that is randomized to receive the *i*th trial arm (1 for intervention and 0 for control) in the *j*th pair of clusters and *k*th group (1 for target and 0 for non‐target group), and ${\bar{c}}_{i}=\sum_{j=1}^{n}c_{\mathrm{ij}}/n$.

^b^ $\beta_{T}$ and $\beta_{D}$: Total effect and direct effect in terms of log incidence rate ratio.

^c^ Components of the variances of $r_{l}^{\text{paired}}\;\left(l=3,4,5\right)$ are: $\mathrm{Var}\left(R_{i}\right)=\frac{1}{n{\bar{p}}_{i\cdot 1}^{2}}\left\{\mathrm{Var}\left(y_{\mathrm{ij}1}\right)+R_{i}^{2}\mathrm{Var}\left(p_{\mathrm{ij}1}\right)-2R_{i}\mathrm{cov}\left(y_{\mathrm{ij}1},p_{\mathrm{ij}1}\right)\right\},i=0,1;$ $\mathrm{cov}\left(R_{1},R_{0}\right)=\frac{1}{n{\bar{p}}_{1\cdot 1}{\bar{p}}_{0\cdot 1}}\left\{\mathrm{cov}\left(y_{1j1},y_{0j1}\right)+R_{1}R_{0}\mathrm{cov}\left(p_{1j1},p_{0j1}\right)-R_{1}\mathrm{cov}\left(y_{0j1},p_{1j1}\right)-R_{0}\mathrm{cov}\left(y_{1j1},p_{0j1}\right)\right\},$ $\mathrm{Var}\left(R_{i}^{*}\right)=\frac{1}{n{\bar{y}}_{i\cdot 0}^{2}}\left\{\mathrm{Var}\left(y_{\mathrm{ij}1}\right)+R_{i}^{*2}\mathrm{Var}\left(y_{\mathrm{ij}0}\right)-2R_{i}^{*}\mathrm{cov}\left(y_{\mathrm{ij}1},y_{\mathrm{ij}0}\right)\right\},i=0,1;$ $\mathrm{cov}\left(R_{1}^{*},R_{0}^{*}\right)=\frac{1}{n{\bar{y}}_{1\cdot 0}{\bar{y}}_{0\cdot 0}}\left\{\mathrm{cov}\left(y_{1j1},y_{0j1}\right)+R_{1}^{*}R_{0}^{*}\mathrm{cov}\left(y_{1j0},y_{0j0}\right)-R_{1}^{*}\mathrm{cov}\left(y_{0j1},y_{1j0}\right)-R_{0}^{*}\mathrm{cov}\left(y_{1j1},y_{0j0}\right)\right\},$ $\mathrm{Var}\left(R_{i}^{†}\right)=\mathrm{Var}\left\{\frac{R_{i1}^{\prime }}{R_{i0}^{\prime }}\right\}={\left(\frac{R_{i1}^{\prime }}{R_{i0}^{\prime }}\right)}^{2}\left(\frac{\mathrm{Var}\left(R_{i1}^{\prime }\right)}{R_{i1}^{\prime 2}}+\frac{\mathrm{Var}\left(R_{i0}^{\prime }\right)}{R_{i0}^{\prime 2}}-\frac{2\mathrm{cov}\left(R_{i1}^{\prime },R_{i0}^{\prime }\right)}{R_{i1}^{\prime }R_{i0}^{\prime }}\right),i=0,1;$ $\mathrm{cov}\left(R_{1}^{†},R_{0}^{†}\right)=\mathrm{cov}\left(\frac{R_{11}^{\prime }}{R_{10}^{\prime }},\frac{R_{01}^{\prime }}{R_{00}^{\prime }}\right)=\frac{1}{nR_{10}^{\prime }R_{00}^{\prime }}\left\{\mathrm{cov}\left(R_{11}^{\prime },R_{01}^{\prime }\right)+R_{1}^{†}R_{0}^{†}\mathrm{cov}\left(R_{10}^{\prime },R_{00}^{\prime }\right)-R_{1}^{†}\mathrm{cov}\left(R_{01}^{\prime },R_{10}^{\prime }\right)-R_{0}^{†}\mathrm{cov}\left(R_{11}^{\prime },R_{00}^{\prime }\right)\right\},$ $\mathrm{Var}\left(R_{\mathrm{ik}}^{\prime }\right)=\frac{1}{n{\bar{p}}_{i\cdot k}^{2}}\left\{\mathrm{Var}\left(y_{\mathrm{ijk}}\right)+R_{\mathrm{ik}}^{\prime 2}\mathrm{Var}\left(p_{\mathrm{ijk}}\right)-2R_{\mathrm{ik}}^{\prime }\mathrm{cov}\left(y_{\mathrm{ijk}},p_{\mathrm{ijk}}\right)\right\},\;i=0,1;k=0,1;$ $\mathrm{cov}\left(R_{\mathrm{ik}}^{\prime },R_{i^{\prime }k^{\prime }}^{\prime }\right)=\frac{1}{n{\bar{p}}_{i\cdot k}{\bar{p}}_{i^{\prime }\cdot k^{\prime }}}\left\{\mathrm{cov}\left(y_{\mathrm{ijk}},y_{i\prime \mathrm{jk}\prime }\right)+R_{\mathrm{ik}}^{\prime }R_{i\prime k\prime }^{\prime }\mathrm{cov}\left(p_{\mathrm{ijk}},p_{i\prime \mathrm{jk}\prime }\right)-R_{\mathrm{ik}}^{\prime }\mathrm{cov}\left(y_{i\prime \mathrm{jk}\prime },p_{\mathrm{ijk}}\right)-R_{i\prime k\prime }^{\prime }\mathrm{cov}\left(y_{\mathrm{ijk}},p_{i\prime \mathrm{jk}\prime }\right)\right\},\;i,i^{\prime }=0,1;k,k^{\prime }=0,1;$ where $R_{\mathrm{ik}}^{\prime }=\frac{\sum_{j=1}^{n}y_{\mathrm{ijk}}}{\sum_{j=1}^{n}p_{\mathrm{ijk}}}\;\text{and}\;{\bar{y}}_{i\cdot k}=\sum_{j=1}^{n}y_{\mathrm{ijk}}/n;{\bar{p}}_{i\cdot k}=\sum_{j=1}^{n}p_{\mathrm{ijk}}/n,i=0,1;k=0,1.$
