# Supplementary material for: Ratio estimators of intervention effects on event rates in cluster randomized trials
Source: Stat Med. 2021 Oct 15;41(1):128–45. doi: 10.1002/sim.9226 (PMC9292872; doi:10.1002/sim.9226)
Supplement: Supplementary file 1 — Online Supplementary Material 1: Comparisons of asymptotically unbiased estimators and derivations of approximately unbiased estimators [file SIM-41-128-s001.pdf]

Online Supplementary Material 1. Comparisons of asymptotically unbiased estimators and derivations of approximately unbiased estimators

A. Comparisons of asymptotically unbiased estimators

With equations (3) and (8), $Var(\bar{c}_i) = Var(R_i)$, $CV(p_{ijk}) = \theta_i \times CV(y_{ijk})$ and the assumption of $CV(y_{ij1}) \cong CV(y_{ij0})$, a comparison of $Var(r_4)$ and $Var(r_2)$ boils down to an evaluation of whether:

$$n_i\left[\frac{Var(R_i^*)}{(R_i^*)^2} - \frac{Var(R_i)}{\bar{c}_i^2}\right]$$

$$= \quad CV(y_{ij1})^2 + CV(y_{ij0})^2 - 2CV(y_{ij1})CV(y_{ij0})corr(y_{ij1}, y_{ij0})$$

$$-\left(\frac{R_i}{\bar{c}_i}\right)^2\left[CV(y_{ij1})^2 + CV(p_{ij1})^2 - 2CV(y_{ij1})CV(p_{ij1})corr(y_{ij1}, p_{ij1})\right]$$

$$\approx \quad 2CV(y_{ij1})^2 - 2CV(y_{ij1})CV(y_{ij1})corr(y_{ij1}, y_{ij0})$$

$$-\left(\frac{R_i}{\bar{c}_i}\right)^2\left[CV(y_{ij1})^2 + \theta_i^2 CV(y_{ij1})^2 - 2\theta_i CV(y_{ij1})CV(y_{ij1})corr(y_{ij1}, p_{ij1})\right]$$

$$= \quad CV(y_{ij1})^2\left[2 - 2corr(y_{ij1}, y_{ij0}) - \left(\frac{R_i}{\bar{c}_i}\right)^2\left[1 + \theta_i^2 - 2\theta_i corr(y_{ij1}, p_{ij1})\right]\right] < 0.$$

Therefore, $Var(r_4) < Var(r_2)$ if in both trial arms:

$$corr(y_{ij1}, y_{ij0}) - \left(\frac{R_i}{\bar{c}_i}\right)^2 \theta_i corr(y_{ij1}, p_{ij1}) > 1 - \left(\frac{R_i}{\bar{c}_i}\right)^2 \times \frac{1 + \theta_i^2}{2}.$$

Furthermore, the difference between the test statistics for r_4 and r_2 is:

$$|t(r_4)| - |t(r_2)| = \frac{|r_4 - 1|}{r_4\sqrt{\frac{Var(R_1^*)}{(R_1^*)^2} + \frac{Var(R_0^*)}{(R_0^*)^2}}} - \frac{|r_2 - 1|}{r_2\sqrt{\frac{Var(R_1)}{\bar{c}_1^2} + \frac{Var(R_0)}{\bar{c}_0^2}}}$$

$$= \frac{\sqrt{\left(\frac{r_4 - 1}{r_4}\right)^2\left(\frac{Var(R_1)}{\bar{c}_1^2} + \frac{Var(R_0)}{\bar{c}_0^2}\right)} - \sqrt{\left(\frac{r_2 - 1}{r_2}\right)^2\left(\frac{Var(R_1^*)}{(R_1^*)^2} + \frac{Var(R_0^*)}{(R_0^*)^2}\right)}}{\sqrt{\left(\frac{Var(R_1^*)}{(R_1^*)^2} + \frac{Var(R_0^*)}{(R_0^*)^2}\right)\left(\frac{Var(R_1)}{\bar{c}_1^2} + \frac{Var(R_0)}{\bar{c}_0^2}\right)}}.$$

Let $r_2 = \zeta r_4$. Then,

$$n_i\left[\left(\frac{r_4 - 1}{r_4}\right)^2\frac{Var(R_i)}{\bar{c}_i^2} - \left(\frac{r_2 - 1}{r_2}\right)^2\frac{Var(R_i^*)}{(R_i^*)^2}\right]$$

$$= n_i\left[\left(\frac{r_4 - 1}{r_4}\right)^2\left(\frac{R_i}{\bar{c}_i}\right)^2\frac{Var(R_i)}{R_i^2} - \left(\frac{r_2 - 1}{r_2}\right)^2\frac{Var(R_i^*)}{(R_i^*)^2}\right]$$

$$= \left(\frac{r_4 - 1}{r_4}\right)^2 \left(\frac{R_i}{\bar{c}_i}\right)^2 \left[CV(y_{ij1})^2 + CV(p_{ij1})^2 - 2CV(y_{ij1})CV(p_{ij1})corr(y_{ij1}, p_{ij1})\right]$$

$$- \left(\frac{r_2 - 1}{r_2}\right)^2 \left[CV(y_{ij1})^2 + CV(y_{ij0})^2 - 2CV(y_{ij1})CV(y_{ij0})corr(y_{ij1}, y_{ij0})\right]$$

$$\approx \left(\frac{r_4 - 1}{r_4}\right)^2 \left(\frac{R_i}{\bar{c}_i}\right)^2 \left[CV(y_{ij1})^2 + \theta_i^2 CV(y_{ij1})^2 - 2\theta_i CV(y_{ij1})^2 corr(y_{ij1}, p_{ij1})\right]$$

$$- \left(\frac{r_2 - 1}{r_2}\right)^2 \left[CV(y_{ij1})^2 + CV(y_{ij1})^2 - 2CV(y_{ij1})^2 corr(y_{ij1}, y_{ij0})\right]$$

$$= CV(y_{ij1})^2 \left[\left(\frac{r_4 - 1}{r_4}\right)^2 \left(\frac{R_i}{\bar{c}_i}\right)^2 \left(1 + \theta_i^2 - 2\theta_i corr(y_{ij1}, p_{ij1})\right)\right.$$

$$\left. - \left(\frac{\zeta r_4 - 1}{\zeta r_4}\right)^2 \left(2 - 2corr(y_{ij1}, y_{ij0})\right)\right].$$

Therefore, $|t(r_2)| < |t(r_4)|$ if in both trial arms:

$$corr(y_{ij1}, y_{ij0}) > 1 - \zeta^2 \left(\frac{r_4 - 1}{\zeta r_4 - 1}\right)^2 \left(\frac{R_i}{\bar{c}_i}\right)^2 \left[\frac{1 + \theta_i^2}{2} - \theta_i corr(y_{ij1}, p_{ij1})\right].$$

Assuming $CV(y_{ij1}) \cong CV(y_{ij0}), CV(p_{ij1}) \cong CV(p_{ij0})$, and $corr(p_{ij1}, p_{ij0}) \approx 1$, a comparison of $Var(r_4)$ versus $Var(r_5)$ boils down to an evaluation of whether:

$$n_i \left[\frac{Var(R_i^\dagger)}{R_i^{\dagger 2}} - \frac{Var(R_i^*)}{(R_i^*)^2}\right]$$

$$= CV(p_{ij1})^2 + CV(p_{ij0})^2 - 2CV(y_{ij1})CV(p_{ij1})corr(y_{ij1}, p_{ij1})$$

$$- 2CV(y_{ij0})CV(p_{ij0})corr(y_{ij0}, p_{ij0}) - 2CV(p_{ij1})CV(p_{ij0})corr(p_{ij1}, p_{ij0})$$

$$+ 2CV(y_{ij1})CV(p_{ij0})corr(y_{ij1}, p_{ij0}) + 2CV(y_{ij0})CV(p_{ij1})corr(y_{ij0}, p_{ij1})$$

$$\approx 2CV(p_{ij1})^2 - 2CV(p_{ij1})^2 corr(p_{ij1}, p_{ij0})$$

$$- 2CV(y_{ij1})CV(p_{ij1})corr(y_{ij1}, p_{ij1}) + 2CV(y_{ij1})CV(p_{ij1})corr(y_{ij1}, p_{ij0})$$

$$+ 2CV(y_{ij1})CV(p_{ij1})corr(y_{ij0}, p_{ij1}) - 2CV(y_{ij1})CV(p_{ij1})corr(y_{ij0}, p_{ij0})$$

$$\approx 2CV(y_{ij1})CV(p_{ij1})\left[corr(y_{ij1}, p_{ij0}) - corr(y_{ij1}, p_{ij1}) - corr(y_{ij0}, p_{ij0})\right.$$

$$\left. + corr(y_{ij0}, p_{ij1})\right] < 0.$$

Therefore, $Var(R_i^\dagger)/(R_i^\dagger)^2 - Var(R_i^*)/(R_i^*)^2 < 0$ and $Var(r_5) < Var(r_4)$ if in both trial arms:

$$corr(y_{ij1}, p_{ij0}) + corr(y_{ij0}, p_{ij1}) < corr(y_{ij0}, p_{ij0}) + corr(y_{ij1}, p_{ij1}).$$

B. Derivations of approximately unbiased estimators

Preamble

Let x and y be random variables with population means $E(x) = \mu_x$ and $E(y) = \mu_y$. Let $\bar{x}$ and $\bar{y}$ be the sample mean of x and y. Define $R = \frac{\mu_y}{\mu_x}, \hat{R} = \frac{\bar{y}}{\bar{x}}, \delta\bar{y} = \frac{\bar{y}-\mu_y}{\mu_y}$ and $\delta\bar{x} = \frac{\bar{x}-\mu_x}{\mu_x}$. Using a second-order Taylor series expansion of $\frac{1}{1+\delta\bar{x}} \approx 1 - \delta\bar{x} + (\delta\bar{x})^2$, and assuming $|\delta\bar{x}| < 1$ and $|\delta\bar{y}| < 1$, we have

$$\frac{1+\delta\bar{y}}{1+\delta\bar{x}} \approx (1+\delta\bar{y})[1-\delta\bar{x}+(\delta\bar{x})^2] = 1+\delta\bar{y}-\delta\bar{x}-\delta\bar{y}\delta\bar{x}+(\delta\bar{x})^2+\delta\bar{y}(\delta\bar{x})^2$$
$$\approx 1+\delta\bar{y}-\delta\bar{x}-\delta\bar{y}\delta\bar{x}+(\delta\bar{x})^2.$$

Then the expectation of $\hat{R}$ is

$$E(\hat{R}) = E\left(R \times \frac{1+\delta\bar{y}}{1+\delta\bar{x}}\right) = R \times E\left[\frac{1+\delta\bar{y}}{1+\delta\bar{x}}\right] \approx R[1 - E(\delta\bar{y}\delta\bar{x}) + E(\delta\bar{x})^2],$$

since $E(\delta\bar{y}) = E(\delta\bar{x}) = 0$.

In studies that have n paired samples of (x, y),

$$E(\delta\bar{x})^2 = \frac{Var(\bar{x})}{\mu_x^2} = \frac{Var(x)}{n\mu_x^2};\ E(\delta\bar{y}\delta\bar{x}) = \frac{cov(\bar{y},\bar{x})}{\mu_y\mu_x} = \frac{cov(y,x)}{n\mu_y\mu_x}.$$

In studies that have two independent samples of x and y, with sample sizes n_x and n_y, respectively,

$$E(\delta\bar{x})^2 = \frac{Var(\bar{x})}{\mu_x^2} = \frac{Var(x)}{n_x\mu_x^2};\ E(\delta\bar{y}\delta\bar{x}) = 0.$$

In cluster randomized trials, define $\delta\bar{y}_{i\cdot k} = \frac{\bar{y}_{i\cdot k}-\bar{Y}_{i\cdot k}}{\bar{Y}_{i\cdot k}}$, where $\bar{y}_{i\cdot 1}$ and $\bar{Y}_{i\cdot 1}$ are sample and population means of number of events in the clusters in the *i*-th trial arm and *k*-th group, respectively, and assume that $|\delta\bar{y}_{i\cdot k}| < 1$. Define $\delta\bar{p}_{i\cdot k} = \frac{\bar{p}_{i\cdot k}-\bar{P}_{i\cdot k}}{\bar{P}_{i\cdot 1}}$, where $\bar{p}_{i\cdot k}$ and $\bar{P}_{i\cdot k}$ are sample and population means of person-time measures in the clusters in the *i*-th trial arm and *k*-th group, respectively, and assume that $|\delta\bar{p}_{i\cdot k}| < 1$.

Ratio of means in matched-pair CRTs

The expectation of the asymptotically unbiased estimator r_1^{paired} and approximately unbiased estimator $r_1^{*,paired}$ of the ratio of means estimator are, respectively:

$$E\left(r_1^{paired}\right) = E\left[\frac{\bar{Y}_{1\cdot 1}}{\bar{Y}_{0\cdot 1}} \times \frac{1+\delta\bar{y}_{1\cdot 1}}{1+\delta\bar{y}_{0\cdot 1}}\right] \approx \frac{\bar{Y}_{1\cdot 1}}{\bar{Y}_{0\cdot 1}}[1 - E(\delta\bar{y}_{1\cdot 1}\delta\bar{y}_{0\cdot 1}) + E(\delta\bar{y}_{0\cdot 1})^2]$$

$$= \frac{\bar{Y}_{1\cdot 1}}{\bar{Y}_{0\cdot 1}}\left[1 - \frac{cov\left(y_{1j1}, y_{0j1}\right)}{n\bar{Y}_{1\cdot 1}\bar{Y}_{0\cdot 1}} + \frac{Var\left(y_{0j1}\right)}{n\bar{Y}_{0\cdot 1}^2}\right].$$

$$r_1^{*,paired} = r_1^{paired} - \left[E\left(r_1^{paired}\right) - \frac{\bar{Y}_{1\cdot 1}}{\bar{Y}_{0\cdot 1}}\right] = r_1^{paired} + \frac{\bar{Y}_{1\cdot 1}}{\bar{Y}_{0\cdot 1}} \times \left[\frac{cov(y_{1j1}, y_{0j1})}{n\bar{Y}_{1\cdot 1}\bar{Y}_{0\cdot 1}} - \frac{Var\left(y_{0j1}\right)}{n\bar{Y}_{0\cdot 1}^2}\right]$$

$$\approx r_1^{paired}\left[1 + \frac{1}{n}CV\left(y_{1j1}\right)CV\left(y_{0j1}\right)corr(y_{1j1}, y_{0j1}) - \frac{1}{n}CV^2\left(y_{0j1}\right)\right],$$

with the unknown population means $\bar{Y}_{0\cdot 1}, \bar{Y}_{1\cdot 1}$ approximated by the sample mean $\bar{y}_{0\cdot 1}, \bar{y}_{1\cdot 1}$ to form the sample *CV*s.

Ratio of mean cluster-level event rates in matched-pair CRTs

$$E\left(r_2^{paired}\right) = E\left[\frac{\bar{C}_1}{\bar{C}_0} \times \frac{1+\delta\bar{c}_{1j}}{1+\delta\bar{c}_{0j}}\right] \approx \frac{\bar{C}_1}{\bar{C}_0}\left[1 - E\left(\delta\bar{c}_{1j}\delta\bar{c}_{0j}\right) + E\left(\delta\bar{c}_{0j}\right)^2\right]$$

$$\approx \frac{\bar{C}_1}{\bar{C}_0}\left[1 - \frac{cov\left(c_{1j}, c_{0j}\right)}{n\bar{C}_1\bar{C}_0} + \frac{Var\left(c_{0j}\right)}{n\bar{C}_0^2}\right],$$

assuming $\left|\delta\bar{c}_{0j}\right| = \left|\frac{\bar{c}_{0j}-\bar{C}_0}{\bar{C}_0}\right| < 1$; $|\delta\bar{c}_{1j}| = |\frac{\bar{c}_{1j}-\bar{C}_1}{\bar{C}_1}| < 1$.

$$r_2^{*,paired} = r_2^{paired} - \left[E\left(r_2^{paired}\right) - \frac{\bar{C}_1}{\bar{C}_0}\right]$$

$$\approx r_2^{paired}\left[1 + \frac{1}{n}CV\left(c_{1j}\right)CV\left(c_{0j}\right)corr(c_{1j}, c_{0j}) - \frac{1}{n}CV^2\left(c_{0j}\right)\right],$$

with the unknown population mean of cluster event rates $\bar{C}_0, \bar{C}_1$ replaced by the sample estimate to form the sample *CV*s.

Ratio of event rates in matched-pair CRTs

$$E\left(r_3^{paired}\right) = E\left[\frac{\bar{Y}_{1\cdot1}/\bar{P}_{1\cdot1}}{\bar{Y}_{0\cdot1}/\bar{P}_{0\cdot1}} \times \frac{(1+\delta\bar{y}_{1\cdot1})/(1+\delta\bar{p}_{1\cdot1})}{(1+\delta\bar{y}_{0\cdot1})/(1+\delta\bar{p}_{0\cdot1})}\right]$$

$$= E\left[\frac{\bar{Y}_{1\cdot1}/\bar{P}_{1\cdot1}}{\bar{Y}_{0\cdot1}/\bar{P}_{0\cdot1}} \times (1+\delta\bar{y}_{1\cdot1})(1+\delta\bar{p}_{0\cdot1}) \times \frac{1}{(1+\delta\bar{y}_{0\cdot1})} \times \frac{1}{(1+\delta\bar{p}_{1\cdot1})}\right]$$

$$\approx \frac{\bar{Y}_{1\cdot1}/\bar{P}_{1\cdot1}}{\bar{Y}_{0\cdot1}/\bar{P}_{0\cdot1}}[1 + E(\delta\bar{y}_{1\cdot1}\delta\bar{p}_{0\cdot1}) - E(\delta\bar{y}_{1\cdot1}\delta\bar{y}_{0\cdot1}) - E(\delta\bar{y}_{1\cdot1}\delta\bar{p}_{1\cdot1})$$

$$- E(\delta\bar{p}_{0\cdot1}\delta\bar{y}_{0\cdot1}) - E(\delta\bar{p}_{0\cdot1}\delta\bar{p}_{1\cdot1}) + E(\delta\bar{y}_{0\cdot1}\delta\bar{p}_{1\cdot1}) + E(\delta\bar{y}_{0\cdot1})^2 + E(\delta\bar{p}_{1\cdot1})^2]$$

$$= \frac{\bar{Y}_{1\cdot1}/\bar{P}_{1\cdot1}}{\bar{Y}_{0\cdot1}/\bar{P}_{0\cdot1}}\left[1 + \frac{cov\left(y_{1j1}, p_{0j1}\right)}{n\bar{Y}_{1\cdot1}\bar{P}_{0\cdot1}} + \frac{cov\left(y_{0j1}, p_{1j1}\right)}{n\bar{Y}_{0\cdot1}\bar{P}_{1\cdot1}} - \frac{cov\left(y_{1j1}, p_{1j1}\right)}{n\bar{Y}_{1\cdot1}\bar{P}_{1\cdot1}}\right.$$

$$- \frac{cov\left(y_{0j1}, p_{0j1}\right)}{n\bar{Y}_{0\cdot1}\bar{P}_{0\cdot1}} - \frac{cov\left(y_{1j1}, y_{0j1}\right)}{n\bar{Y}_{1\cdot1}\bar{Y}_{0\cdot1}} - \frac{cov\left(p_{1j1}, p_{0j1}\right)}{n\bar{P}_{1\cdot1}\bar{P}_{0\cdot1}} + \frac{Var\left(y_{0j1}\right)}{n\bar{Y}_{0\cdot1}^2}$$

$$\left. + \frac{Var\left(p_{1j1}\right)}{n\bar{P}_{1\cdot1}^2}\right].$$

$$r_3^{*,paired} = r_3^{paired}$$

$$+ \frac{\bar{Y}_{1\cdot1}/\bar{P}_{1\cdot1}}{\bar{Y}_{0\cdot1}/\bar{P}_{0\cdot1}}$$

$$\times\left[-\frac{cov\left(y_{1j1}, p_{0j1}\right)}{n\bar{Y}_{1\cdot1}\bar{P}_{0\cdot1}} - \frac{cov\left(y_{0j1}, p_{1j1}\right)}{n\bar{Y}_{0\cdot1}\bar{P}_{1\cdot1}} + \frac{cov\left(y_{1j1}, y_{0j1}\right)}{n\bar{Y}_{1\cdot1}\bar{Y}_{0\cdot1}} + \frac{cov\left(p_{1j1}, p_{0j1}\right)}{n\bar{P}_{1\cdot1}\bar{P}_{0\cdot1}}\right.$$

$$\left. + \frac{cov\left(y_{1j1}, p_{1j1}\right)}{n\bar{Y}_{1\cdot1}\bar{P}_{1\cdot1}} + \frac{cov\left(y_{0j1}, p_{0j1}\right)}{n\bar{Y}_{0\cdot1}\bar{P}_{0\cdot1}} - \frac{Var\left(y_{0j1}\right)}{n\bar{Y}_{0\cdot1}^2} - \frac{Var\left(p_{1j1}\right)}{n\bar{P}_{1\cdot1}^2}\right]$$

$$\approx r_3^{paired}\left[1 + \frac{1}{n}\sum_{i=0}^{1}\sum_{i'=0}^{1}(-1)^{i+i'}CV\left(y_{ij1}\right)CV\left(p_{i'j1}\right)corr\left(y_{ij1}, p_{i'j1}\right)\right.$$

$$+ \frac{1}{n}CV\left(y_{1j1}\right)CV\left(y_{0j1}\right)corr\left(y_{1j1}, y_{0j1}\right)$$

$$\left. + \frac{1}{n}CV\left(p_{1j1}\right)CV\left(p_{0j1}\right)corr\left(p_{1j1}, p_{0j1}\right) - \frac{1}{n}CV^2\left(y_{0j1}\right) - \frac{1}{n}CV^2\left(p_{1j1}\right)\right],$$

with the unknown population means $\bar{Y}_{i\cdot1}$ and $\bar{P}_{i\cdot1}$ $(i = 0,1)$ replaced by their sample estimates to form the sample *CV*s.

Double ratio of counts in matched-pair CRTs

$E\left(r_4^{paired}\right)$ and $r_4^{*,paired}$ can be obtained by replacing r_3^{paired}, p_{ij1} and $\bar{P}_{i\cdot1}$ by r_4^{paired}, y_{ij0}, and $\bar{Y}_{i\cdot0}$ $(i = 0$ or $1)$ in the formula in the previous sub-section on ratio of event rates in matched-pair CRTs.

Double ratio of event rates in matched-pair CRTs

$$E\left(r_5^{paired}\right) = E\left[\frac{\frac{\bar{Y}_{1\cdot 1}}{\bar{P}_{1\cdot 1}}\Big/\frac{\bar{Y}_{1\cdot 0}}{\bar{P}_{1\cdot 0}}}{\frac{\bar{Y}_{0\cdot 1}}{\bar{P}_{0\cdot 1}}\Big/\frac{\bar{Y}_{0\cdot 0}}{\bar{P}_{0\cdot 0}}} \times \frac{\frac{(1+\delta\bar{y}_{1\cdot 1})}{(1+\delta\bar{p}_{1\cdot 1})}\Big/\frac{(1+\delta\bar{y}_{1\cdot 0})}{(1+\delta\bar{p}_{1\cdot 0})}}{\frac{(1+\delta\bar{y}_{0\cdot 1})}{(1+\delta\bar{p}_{0\cdot 1})}\Big/\frac{(1+\delta\bar{y}_{0\cdot 0})}{(1+\delta\bar{p}_{0\cdot 0})}}\right]$$

$$= E\left[\frac{\frac{\bar{Y}_{1\cdot 1}}{\bar{P}_{1\cdot 1}}\Big/\frac{\bar{Y}_{1\cdot 0}}{\bar{P}_{1\cdot 0}}}{\frac{\bar{Y}_{0\cdot 1}}{\bar{P}_{0\cdot 1}}\Big/\frac{\bar{Y}_{0\cdot 0}}{\bar{P}_{0\cdot 0}}} \times (1+\delta\bar{y}_{1\cdot 1})(1+\delta\bar{p}_{10})\frac{1}{(1+\delta\bar{y}_{1\cdot 0})}\frac{1}{(1+\delta\bar{p}_{1\cdot 1})}(1+\delta\bar{y}_{0\cdot 0})(1 + \delta\bar{p}_{0\cdot 1})\frac{1}{(1+\delta\bar{y}_{0\cdot 1})}\frac{1}{(1+\delta\bar{p}_{0\cdot 0})}\right]$$

$$\approx \frac{\frac{\bar{Y}_{1\cdot 1}}{\bar{P}_{1\cdot 1}}\Big/\frac{\bar{Y}_{1\cdot 0}}{\bar{P}_{1\cdot 0}}}{\frac{\bar{Y}_{0\cdot 1}}{\bar{P}_{0\cdot 1}}\Big/\frac{\bar{Y}_{0\cdot 0}}{\bar{P}_{0\cdot 0}}} \times [1 + E(\delta\bar{y}_{1\cdot 1}\delta\bar{p}_{1\cdot 0}) + E(\delta\bar{y}_{1\cdot 1}\delta\bar{y}_{0\cdot 0}) + E(\delta\bar{y}_{1\cdot 1}\delta\bar{p}_{0\cdot 1}) - E(\delta\bar{y}_{1\cdot 1}\delta\bar{y}_{1\cdot 0}) - E(\delta\bar{y}_{1\cdot 1}\delta\bar{p}_{1\cdot 1}) - E(\delta\bar{y}_{1\cdot 1}\delta\bar{y}_{0\cdot 1}) - E(\delta\bar{y}_{1\cdot 1}\delta\bar{p}_{0\cdot 0}) + E(\delta\bar{p}_{1\cdot 0}\delta\bar{y}_{0\cdot 0}) + E(\delta\bar{p}_{1\cdot 0}\delta\bar{p}_{0\cdot 1}) - E(\delta\bar{p}_{1\cdot 0}\delta\bar{y}_{1\cdot 0}) - E(\delta\bar{p}_{1\cdot 0}\delta\bar{p}_{1\cdot 1}) - E(\delta\bar{p}_{1\cdot 0}\delta\bar{y}_{0\cdot 1}) - E(\delta\bar{p}_{1\cdot 0}\delta\bar{p}_{0\cdot 0}) + E(\delta\bar{y}_{0\cdot 0}\delta\bar{p}_{0\cdot 1}) - E(\delta\bar{y}_{0\cdot 0}\delta\bar{y}_{1\cdot 0}) - E(\delta\bar{y}_{0\cdot 0}\delta\bar{p}_{1\cdot 1}) - E(\delta\bar{y}_{0\cdot 0}\delta\bar{y}_{0\cdot 1}) - E(\delta\bar{y}_{0\cdot 0}\delta\bar{p}_{0\cdot 0}) - E(\delta\bar{p}_{0\cdot 1}\delta\bar{y}_{1\cdot 0}) - E(\delta\bar{p}_{0\cdot 1}\delta\bar{x}_{1\cdot 1}) - E(\delta\bar{p}_{0\cdot 1}\delta\bar{y}_{0\cdot 1}) - E(\delta\bar{p}_{0\cdot 1}\delta\bar{p}_{0\cdot 0}) + E(\delta\bar{y}_{1\cdot 0}\delta\bar{p}_{1\cdot 1}) + E(\delta\bar{y}_{1\cdot 0}\delta\bar{y}_{0\cdot 1}) + E(\delta\bar{y}_{1\cdot 0}\delta\bar{p}_{0\cdot 0}) + E(\delta\bar{p}_{1\cdot 1}\delta\bar{y}_{0\cdot 1}) + E(\delta\bar{p}_{1\cdot 1}\delta\bar{p}_{0\cdot 0}) + E(\delta\bar{y}_{0\cdot 1}\delta\bar{p}_{0\cdot 0}) + E(\delta\bar{y}_{1\cdot 0})^2 + E(\delta\bar{p}_{1\cdot 1})^2 + E(\delta\bar{y}_{0\cdot 1})^2 + E(\delta\bar{p}_{0\cdot 0})^2]$$

$$\approx \frac{\frac{\bar{Y}_{1\cdot 1}}{\bar{P}_{1\cdot 1}}\Big/\frac{\bar{Y}_{1\cdot 0}}{\bar{P}_{1\cdot 0}}}{\frac{\bar{Y}_{0\cdot 1}}{\bar{P}_{0\cdot 1}}\Big/\frac{\bar{Y}_{0\cdot 0}}{\bar{P}_{0\cdot 0}}}\left[1 - \sum_{i=0}^{1}\sum_{i'=0}^{1}\sum_{k=0}^{1}\sum_{k'=0}^{1}(-1)^{i+i'+k+k'}\frac{cov\left(y_{ijk}, p_{i'jk'}\right)}{n\bar{Y}_{i\cdot k}\bar{P}_{i'\cdot k'}} + \sum_{i=0}^{1}\sum_{i'=0}^{1}\sum_{k=0}^{1}\sum_{k'=0}^{1}(-1)^{i+i'+k+k'}\frac{cov\left(y_{ijk}, y_{i'jk'}\right)}{2n\bar{Y}_{i\cdot k}\bar{Y}_{i'\cdot k'}} + \frac{Var\left(y_{1j0}\right)}{2n\bar{Y}_{1\cdot 0}^2} + \frac{Var\left(y_{0j1}\right)}{2n\bar{Y}_{0\cdot 1}^2} - \frac{Var\left(y_{1j1}\right)}{2n\bar{Y}_{1\cdot 1}^2} - \frac{Var\left(y_{0j0}\right)}{2n\bar{Y}_{0\cdot 0}^2} + \sum_{i=0}^{1}\sum_{i'=0}^{1}\sum_{k=0}^{1}\sum_{k'=0}^{1}(-1)^{i+i'+k+k'}\frac{cov\left(p_{ijk}, p_{i'jk'}\right)}{2n\bar{P}_{i\cdot k}\bar{P}_{i'\cdot k'}} + \frac{Var\left(p_{1j1}\right)}{2n\bar{P}_{1\cdot 1}^2} + \frac{Var\left(p_{0j0}\right)}{2n\bar{P}_{0\cdot 0}^2} - \frac{Var\left(p_{1j0}\right)}{2n\bar{P}_{1\cdot 0}^2} - \frac{Var\left(p_{0j1}\right)}{2n\bar{P}_{0\cdot 1}^2}\right].$$

$$
\begin{aligned}
r_5^{*,paired} \approx r_5^{paired} & \\
& + \frac{\frac{\bar{Y}_{1\cdot 1}}{\bar{P}_{1\cdot 1}} \Big/ \frac{\bar{Y}_{1\cdot 0}}{\bar{P}_{1\cdot 0}}}{\frac{\bar{Y}_{0\cdot 1}}{\bar{P}_{0\cdot 1}} \Big/ \frac{\bar{Y}_{0\cdot 0}}{\bar{P}_{0\cdot 0}}} \\
& \times \left\{ \sum_{i=0}^{1}\sum_{i'=0}^{1}\sum_{k=0}^{1}\sum_{k'=0}^{1} (-1)^{i+i'+k+k'} \left[\frac{cov(y_{ijk}, p_{i'jk'})}{n\bar{Y}_{i\cdot k}\bar{P}_{i'\cdot k'}} - \frac{cov(y_{ijk}, y_{i'jk'})}{2n\bar{Y}_{i\cdot k}\bar{Y}_{i'\cdot k'}} \right.\right. \\
& \left. - \frac{cov(p_{ijk}, p_{i'jk'})}{2n\bar{P}_{i\cdot k}\bar{P}_{i'\cdot k'}} \right] - \frac{Var(y_{1j0})}{2n\bar{Y}_{1\cdot 0}^2} - \frac{Var(y_{0j1})}{2n\bar{Y}_{0\cdot 1}^2} + \frac{Var(y_{1j1})}{2n\bar{Y}_{1\cdot 1}^2} + \frac{Var(y_{0j0})}{2n\bar{Y}_{0\cdot 0}^2} \\
& \left. - \frac{Var(p_{1j1})}{2n\bar{P}_{1\cdot 1}^2} - \frac{Var(p_{0j0})}{2n\bar{P}_{0\cdot 0}^2} + \frac{Var(p_{1j0})}{2n\bar{P}_{1\cdot 0}^2} + \frac{Var(p_{0j1})}{2n\bar{P}_{0\cdot 1}^2} \right\} \\
& \approx r_4^{paired} \left\{ 1 \right. \\
& + \frac{1}{2n} \sum_{i=0}^{1}\sum_{i'=0}^{1}\sum_{k=0}^{1}\sum_{k'=0}^{1} (-1)^{i+i'+k+k'} \left[2CV(y_{ijk})CV(p_{i'jk'})corr(y_{ijk}, p_{i'jk'}) \right. \\
& - CV(y_{ijk})CV(y_{i'jk'})corr(y_{ijk}, y_{i'jk'}) \\
& \left. - CV(p_{ijk})CV(p_{i'jk'})corr(p_{ijk}, p_{i'jk'}) \right] \\
& \left. + \frac{1}{2n} \sum_{i=0}^{1}\sum_{k=0}^{1} (-1)^{i+k} \left[CV^2(y_{ijk}) - CV^2(p_{ijk}) \right] \right\}.
\end{aligned}
$$

Variances of approximately unbiased estimators in matched-pair CRT

The variance of $r_1^{*,paired}$ is:

$$
\begin{aligned}
Var(r_1^{*,paired}) &= E\left[r_1^{*,paired} - E(r_1^{*,paired}) \right]^2 \\
&= E\left\{ r_1^{paired} + \frac{\bar{Y}_{1\cdot 1}}{\bar{Y}_{0\cdot 1}} \times \left[\frac{cov(y_{1j1}, y_{0j1})}{n\bar{Y}_{1\cdot 1}\bar{Y}_{0\cdot 1}} - \frac{Var(y_{0j1})}{n\bar{Y}_{0\cdot 1}^2} \right] - \frac{\bar{Y}_{1\cdot 1}}{\bar{Y}_{0\cdot 1}} \right\}^2 \\
&= E\left[r_1^{paired} - E(r_1^{paired}) \right]^2 = Var(r_1^{paired}).
\end{aligned}
$$

Following the same steps, it can be shown that $Var(r_l^{*,paired}) \approx Var(r_l^{paired})$ for $l =$ 2, 3, 4 and 5 as well.

Non-matched CRTs

Derivations of the approximately unbiased estimators and their variances for non-matched CRTs follow the same procedures above. Their formulas are shown in the main body of the manuscript.
