# Supplementary material for: Ratio estimators of intervention effects on event rates in cluster randomized trials
Source: Stat Med. 2021 Oct 15;41(1):128–45. doi: 10.1002/sim.9226 (PMC9292872; doi:10.1002/sim.9226)
Supplement: Supplementary file 2 — Online Supplementary Material 2: Further simulation results, by number of clusters per trial arm and three levels of SD (α ij ); mean population size per cluster is 100 [file SIM-41-128-s002.pdf]

Online Supplementary Material 2. Further simulation results, by number of clusters per trial arm and three levels of $SD(\alpha_{ij})$; mean population size per cluster is 100.

Table of Contents
**Figures S1 to S26: Non-matched cluster randomized trials; population per cluster follows a skewed distribution.**
Figure S1. Relative bias of intervention effect estimators; CV of population size per cluster is 0.2; intervention has a direct effect only ($\exp(\beta_D) = 0.5;\ \exp(\beta_I) = 1$).
Figure S2. Relative bias of intervention effect estimators; CV of population size per cluster is 0.6; intervention has a direct effect only ($\exp(\beta_D) = 0.5;\ \exp(\beta_I) = 1$).
Figure S3. Root mean squared error (RMSE) of intervention effect estimators; CV of population size per cluster is 0.2; intervention has a direct effect only ($\exp(\beta_D) = 0.5;\ \exp(\beta_I) = 1$).
Figure S4. Root mean squared error (RMSE) of intervention effect estimators; CV of population size per cluster is 0.6; intervention has a direct effect only ($\exp(\beta_D) = 0.5;\ \exp(\beta_I) = 1$).
Figure S5. Coverage probability (CP) of 95% confidence interval (calculated on log-scale) of intervention effect estimators; CV of population size per cluster is 0.2; intervention has a direct effect only ($\exp(\beta_D) = 0.5;\ \exp(\beta_I) = 1$).
Figure S6. Coverage probability (CP) of 95% confidence interval (calculated on log-scale) of intervention effect estimators; CV of population size per cluster is 0.6; intervention has a direct effect only ($\exp(\beta_D) = 0.5;\ \exp(\beta_I) = 1$).
Figure S7. Power of $r_2^*, r_{3(J)}^*, r_{4(J)}^*$, and $r_{5(J)}^*$; CV of population size per cluster is 0.2. Upper panel: with direct effect only ($\exp(\beta_D) = 0.5;\ \exp(\beta_I) = 1$); lower panel: with direct and indirect effects ($\exp(\beta_D) = 0.5;\ \exp(\beta_I) = 0.75$).
Figure S8. Power of $r_2^*, r_{3(J)}^*, r_{4(J)}^*$, and $r_{5(J)}^*$; CV of population size per cluster is 0.6. Upper panel: with direct effect only ($\exp(\beta_D) = 0.5;\ \exp(\beta_I) = 1$); lower panel: with direct and indirect effects ($\exp(\beta_D) = 0.5;\ \exp(\beta_I) = 0.75$).
Figure S9. Relative bias of intervention effect estimators; CV of population size per cluster is 0.2; intervention has direct and indirect effects ($\exp(\beta_D) = 0.5;\ \exp(\beta_I) = 0.75$).
Figure S10. Relative bias of intervention effect estimators; CV of population size per cluster is 0.4; intervention has direct and indirect effects ($\exp(\beta_D) = 0.5;\ \exp(\beta_I) = 0.75$).
Figure S11. Relative bias of intervention effect estimators; CV of population size per cluster is 0.6; intervention has direct and indirect effects ($\exp(\beta_D) = 0.5;\ \exp(\beta_I) = 0.75$).
Figure S12. Root mean squared error (RMSE) of intervention effect estimators; CV of population size per cluster is 0.2; intervention has direct and indirect effects ($\exp(\beta_D) = 0.5;\ \exp(\beta_I) = 0.75$).
Figure S13. Root mean squared error (RMSE) of intervention effect estimators; CV of population size per cluster is 0.4; intervention has direct and indirect effects ($\exp(\beta_D) = 0.5;\ \exp(\beta_I) = 0.75$).
Figure S14. Root mean squared error (RMSE) of intervention effect estimators; CV of population size per cluster is 0.6; intervention has direct and indirect effects ($\exp(\beta_D) = 0.5;\ \exp(\beta_I) = 0.75$).

Figure S15. Coverage probability (CP) of 95% confidence interval (calculated on log-scale) of intervention effect estimators; CV of population size per cluster is 0.2; intervention has direct and indirect effects ($\exp(\beta_D) = 0.5;\ \exp(\beta_I) = 0.75$).
Figure S16. Coverage probability (CP) of 95% confidence interval (calculated on log-scale) of intervention effect estimators; CV of population size per cluster is 0.4; intervention has direct and indirect effects ($\exp(\beta_D) = 0.5;\ \exp(\beta_I) = 0.75$).
Figure S17. Coverage probability (CP) of 95% confidence interval (calculated on log-scale) of intervention effect estimators; CV of population size per cluster is 0.6; intervention has direct and indirect effects ($\exp(\beta_D) = 0.5;\ \exp(\beta_I) = 0.75$).
Figure S18. Relative bias of intervention effect estimators; CV of population size per cluster is 0.2; intervention has no effect ($\exp(\beta_D) = 1;\ \exp(\beta_I) = 1$).
Figure S19. Relative bias of intervention effect estimators; CV of population size per cluster is 0.4; intervention has no effect ($\exp(\beta_D) = 1;\ \exp(\beta_I) = 1$).
Figure S20. Relative bias of intervention effect estimators; CV of population size per cluster is 0.6; intervention has no effect ($\exp(\beta_D) = 1;\ \exp(\beta_I) = 1$).
Figure S21. Root mean squared error (RMSE) of intervention effect estimators; CV of population size per cluster is 0.2; intervention has no effect ($\exp(\beta_D) = 1;\ \exp(\beta_I) = 1$).
Figure S22. Root mean squared error (RMSE) of intervention effect estimators; CV of population size per cluster is 0.4; intervention has no effect ($\exp(\beta_D) = 1;\ \exp(\beta_I) = 1$).
Figure S23. Root mean squared error (RMSE) of intervention effect estimators; CV of population size per cluster is 0.6; intervention has no effect ($\exp(\beta_D) = 1;\ \exp(\beta_I) = 1$).
Figure S24. Type 1 error rate; CV of population size per cluster is 0.2; intervention has no effect ($\exp(\beta_D) = 1;\ \exp(\beta_I) = 1$).
Figure S25. Type 1 error rate; CV of population size per cluster is 0.4; intervention has no effect ($\exp(\beta_D) = 1;\ \exp(\beta_I) = 1$).
Figure S26. Type 1 error rate; CV of population size per cluster is 0.6; intervention has no effect ($\exp(\beta_D) = 1;\ \exp(\beta_I) = 1$).
**Figures S27 to S33: Non-matched cluster randomized trials; population per cluster follows a normal distribution.**
Figure S27. Relative bias of intervention effect estimators; CV of population size per cluster is 0.2; intervention has a direct effect only ($\exp(\beta_D) = 0.5;\ \exp(\beta_I) = 1$).
Figure S28. Relative bias of intervention effect estimators; CV of population size per cluster is 0.4; intervention has a direct effect only ($\exp(\beta_D) = 0.5;\ \exp(\beta_I) = 1$).
Figure S29. Root mean squared error (RMSE) of intervention effect estimators; CV of population size per cluster is 0.2; intervention has a direct effect only ($\exp(\beta_D) = 0.5;\ \exp(\beta_I) = 1$).
Figure S30. Root mean squared error (RMSE) of intervention effect estimators; CV of population size per cluster is 0.4; intervention has a direct effect only ($\exp(\beta_D) = 0.5;\ \exp(\beta_I) = 1$).
Figure S31. Coverage probability (CP) of 95% confidence interval (calculated on log-scale) of intervention effect estimators; CV of population size per cluster is 0.2; intervention has a direct effect only ($\exp(\beta_D) = 0.5;\ \exp(\beta_I) = 1$).
Figure S32. Coverage probability (CP) of 95% confidence interval (calculated on log-scale) of intervention effect estimators ; CV of population size per cluster is 0.4; intervention has a direct effect only ($\exp(\beta_D) = 0.5;\ \exp(\beta_I) = 1$).
Figure S33. Power of $r_2^*, r_{3(J)}^*, r_{4(J)}^*$, and $r_{5(J)}^*$; CV of population size per cluster is 0.2, 0.4, respectively; intervention has a direct effect only ($\exp(\beta_D) = 0.5;\ \exp(\beta_I) = 1$). Upper panel: $CV = 0.2$; lower panel: $CV = 0.4$.

**Figures S34 to S63: Matched-pair cluster randomized trials; population per cluster follows a skewed distribution.**
Figure S34. Relative bias of intervention effect estimators; CV of population size per cluster is 0.2; intervention has a direct effect only $(\exp(\beta_D) = 0.5;\ \exp(\beta_I) = 1)$.
Figure S35. Relative bias of intervention effect estimators; CV of population size per cluster is 0.4; intervention has a direct effect only $(\exp(\beta_D) = 0.5;\ \exp(\beta_I) = 1)$.
Figure S36. Relative bias of intervention effect estimators; CV of population size per cluster is 0.6; intervention has a direct effect only $(\exp(\beta_D) = 0.5;\ \exp(\beta_I) = 1)$.
Figure S37. Root mean squared error (RMSE) of intervention effect estimators; CV of population size per cluster is 0.2; intervention has a direct effect only $(\exp(\beta_D) = 0.5;\ \exp(\beta_I) = 1)$.
Figure S38. Root mean squared error (RMSE) of intervention effect estimators; CV of population size per cluster is 0.4; intervention has a direct effect only $(\exp(\beta_D) = 0.5;\ \exp(\beta_I) = 1)$.
Figure S39. Root mean squared error (RMSE) of intervention effect estimators; CV of population size per cluster is 0.6; intervention has a direct effect only $(\exp(\beta_D) = 0.5;\ \exp(\beta_I) = 1)$.
Figure S40. Coverage probability (CP) of 95% confidence interval (calculated on log-scale) of intervention effect estimators; CV of population size per cluster is 0.2; intervention has a direct effect only $(\exp(\beta_D) = 0.5;\ \exp(\beta_I) = 1)$.
Figure S41. Coverage probability (CP) of 95% confidence interval (calculated on log-scale) of intervention effect estimators; CV of population size per cluster is 0.4; intervention has a direct effect only $(\exp(\beta_D) = 0.5;\ \exp(\beta_I) = 1)$.
Figure S42. Coverage probability (CP) of 95% confidence interval (calculated on log-scale) of intervention effect estimators; CV of population size per cluster is 0.6; intervention has a direct effect only $(\exp(\beta_D) = 0.5;\ \exp(\beta_I) = 1)$.
Figure S43. Power of $r_2^*, r_{3(J)}^*, r_{4(J)}^*$, and $r_{5(J)}^*$; CV of population size per cluster is 0.2. Upper panel: with direct effect only $(\exp(\beta_D) = 0.5;\ \exp(\beta_I) = 1)$; lower panel: with direct and indirect effects $(\exp(\beta_D) = 0.5;\ \exp(\beta_I) = 0.75)$.
Figure S44. Power of $r_2^*, r_{3(J)}^*, r_{4(J)}^*$, and $r_{5(J)}^*$; CV of population size per cluster is 0.4. Upper panel: with direct effect only $(\exp(\beta_D) = 0.5;\ \exp(\beta_I) = 1)$; lower panel: with direct and indirect effects $(\exp(\beta_D) = 0.5;\ \exp(\beta_I) = 0.75)$.
Figure S45. Power of $r_2^*, r_{3(J)}^*, r_{4(J)}^*$, and $r_{5(J)}^*$; CV of population size per cluster is 0.6. Upper panel: with direct effect only $(\exp(\beta_D) = 0.5;\ \exp(\beta_I) = 1)$; lower panel: with direct and indirect effects $(\exp(\beta_D) = 0.5;\ \exp(\beta_I) = 0.75)$.
Figure S46. Relative bias of intervention effect estimators; CV of population size per cluster is 0.2; intervention has direct and indirect effects $(\exp(\beta_D) = 0.5;\ \exp(\beta_I) = 0.75)$.
Figure S47. Relative bias of intervention effect estimators; CV of population size per cluster is 0.4; intervention has direct and indirect effects $(\exp(\beta_D) = 0.5;\ \exp(\beta_I) = 0.75)$.
Figure S48. Relative bias of intervention effect estimators; CV of population size per cluster is 0.6; intervention has direct and indirect effects $(\exp(\beta_D) = 0.5;\ \exp(\beta_I) = 0.75)$.
Figure S49. Root mean squared error (RMSE) of intervention effect estimators; CV of population size per cluster is 0.2; intervention has direct and indirect effects $(\exp(\beta_D) = 0.5;\ \exp(\beta_I) = 0.75)$.

Figure S50. Root mean squared error (RMSE) of intervention effect estimators; CV of population size per cluster is 0.4; intervention has direct and indirect effects $(\exp(\beta_D) = 0.5;\ \exp(\beta_I) = 0.75)$.
Figure S51. Root mean squared error (RMSE) of intervention effect estimators; CV of population size per cluster is 0.6; intervention has direct and indirect effects $(\exp(\beta_D) = 0.5;\ \exp(\beta_I) = 0.75)$.
Figure S52. Coverage probability (CP) of 95% confidence interval (calculated on log-scale) of intervention effect estimators; CV of population size per cluster is 0.2; intervention has direct and indirect effects $(\exp(\beta_D) = 0.5;\ \exp(\beta_I) = 0.75)$.
Figure S53. Coverage probability (CP) of 95% confidence interval (calculated on log-scale) of intervention effect estimators; CV of population size per cluster is 0.4; intervention has direct and indirect effects $(\exp(\beta_D) = 0.5;\ \exp(\beta_I) = 0.75)$.
Figure S54. Coverage probability (CP) of 95% confidence interval (calculated on log-scale) of intervention effect estimators; CV of population size per cluster is 0.6; intervention has direct and indirect effects $(\exp(\beta_D) = 0.5;\ \exp(\beta_I) = 0.75)$.
Figure S55. Relative bias of intervention effect estimators; CV of population size per cluster is 0.2; intervention has no effect $(\exp(\beta_D) = 1;\ \exp(\beta_I) = 1)$.
Figure S56. Relative bias of intervention effect estimators; CV of population size per cluster is 0.4; intervention has no effect $(\exp(\beta_D) = 1;\ \exp(\beta_I) = 1)$.
Figure S57. Relative bias of intervention effect estimators; CV of population size per cluster is 0.6; intervention has no effect $(\exp(\beta_D) = 1;\ \exp(\beta_I) = 1)$.
Figure S58. Root mean squared error (RMSE) of intervention effect estimators; CV of population size per cluster is 0.2; intervention has no effect $(\exp(\beta_D) = 1;\ \exp(\beta_I) = 1)$.
Figure S59. Root mean squared error (RMSE) of intervention effect estimators; CV of population size per cluster is 0.4; intervention has no effect $(\exp(\beta_D) = 1;\ \exp(\beta_I) = 1)$.
Figure S60. Root mean squared error (RMSE) of intervention effect estimators; CV of population size per cluster is 0.6; intervention has no effect $(\exp(\beta_D) = 1;\ \exp(\beta_I) = 1)$.
Figure S61. Type 1 error rate; CV of population size per cluster is 0.2; intervention has no effect $(\exp(\beta_D) = 1;\ \exp(\beta_I) = 1)$.
Figure S62. Type 1 error rate; CV of population size per cluster is 0.4; intervention has no effect $(\exp(\beta_D) = 1;\ \exp(\beta_I) = 1)$.
Figure S63. Type 1 error rate; CV of population size per cluster is 0.6; intervention has no effect $(\exp(\beta_D) = 1;\ \exp(\beta_I) = 1)$.
**Figures S64 to S70: Matched-pair cluster randomized trials; population per cluster follows a normal distribution.**
Figure S64. Relative bias of intervention effect estimators; CV of population size per cluster is 0.2; intervention has a direct effect only $(\exp(\beta_D) = 0.5;\ \exp(\beta_I) = 1)$.
Figure S65. Relative bias of intervention effect estimators; CV of population size per cluster is 0.4; intervention has a direct effect only $(\exp(\beta_D) = 0.5;\ \exp(\beta_I) = 1)$.
Figure S66. Root mean squared error (RMSE) of intervention effect estimators; CV of population size per cluster is 0.2; intervention has a direct effect only $(\exp(\beta_D) = 0.5;\ \exp(\beta_I) = 1)$.
Figure S67. Root mean squared error (RMSE) of intervention effect estimators; CV of population size per cluster is 0.4; intervention has a direct effect only $(\exp(\beta_D) = 0.5;\ \exp(\beta_I) = 1)$.
Figure S68. Coverage probability (CP) of 95% confidence interval (calculated on log-scale) of intervention effect estimators; CV of population size per cluster is 0.2; intervention has a direct effect only $(\exp(\beta_D) = 0.5;\ \exp(\beta_I) = 1)$.

Figure S69. Coverage probability (CP) of 95% confidence interval (calculated on log-scale) of intervention effect estimators; CV of population size per cluster is 0.4; intervention has a direct effect only $(\exp(\beta_D) = 0.5;\ \exp(\beta_I) = 1)$.
Figure S70. Power of $r_2^*, r_{3(J)}^*, r_{4(J)}^*$, and $r_{5(J)}^*$; CV of population size per cluster is 0.2, 0.4, respectively; intervention has a direct effect only $(\exp(\beta_D) = 0.5;\ \exp(\beta_I) = 1)$. Upper panel: $CV = 0.2$; lower panel: $CV = 0.4$.

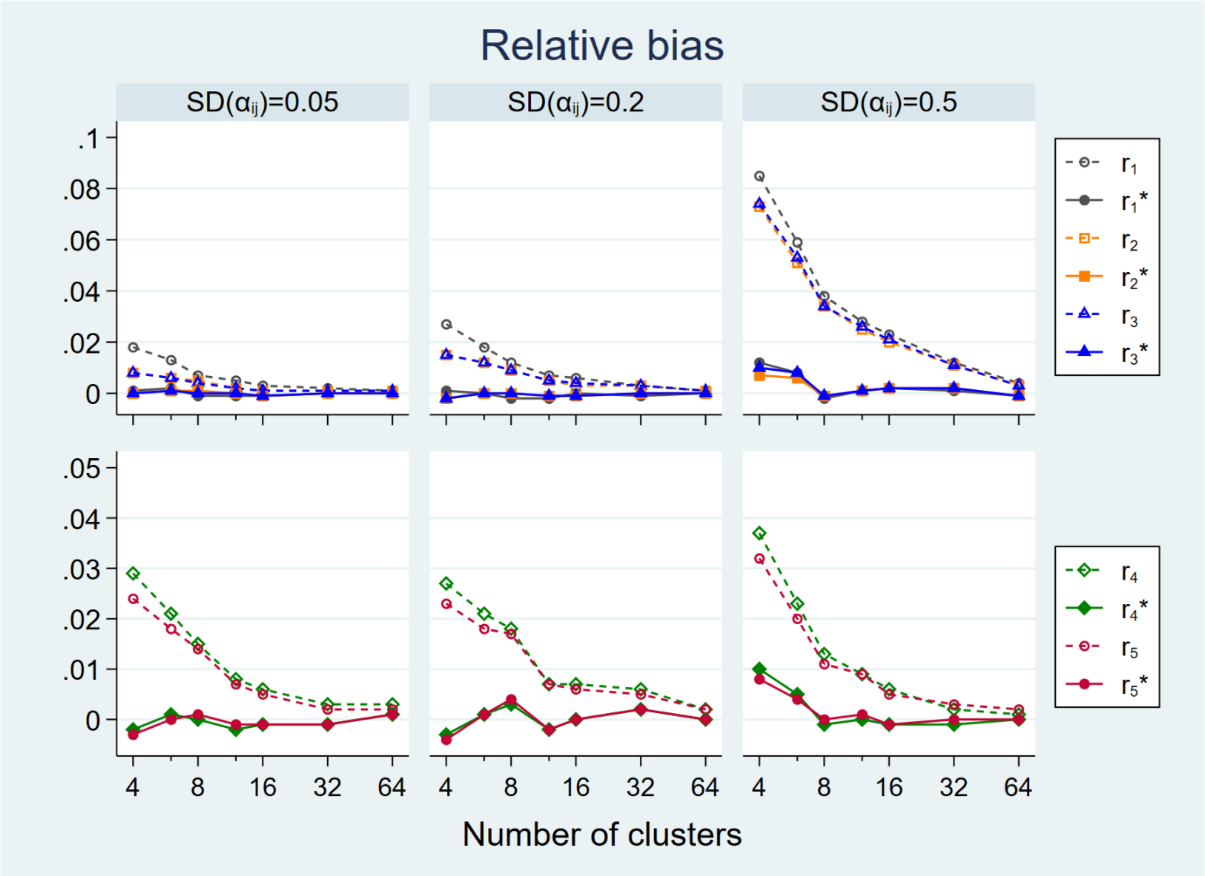

Figure S1. Relative bias of intervention effect estimators in relation to the number of clusters per trial arm for non-matched CRTs, by three levels of $SD(\alpha_{ij})$; population size per cluster follows a skewed distribution with mean = 100 and $CV = 0.2$; intervention has a direct effect only $(\exp(\beta_D) = 0.5;\ \exp(\beta_I) = 1)$.

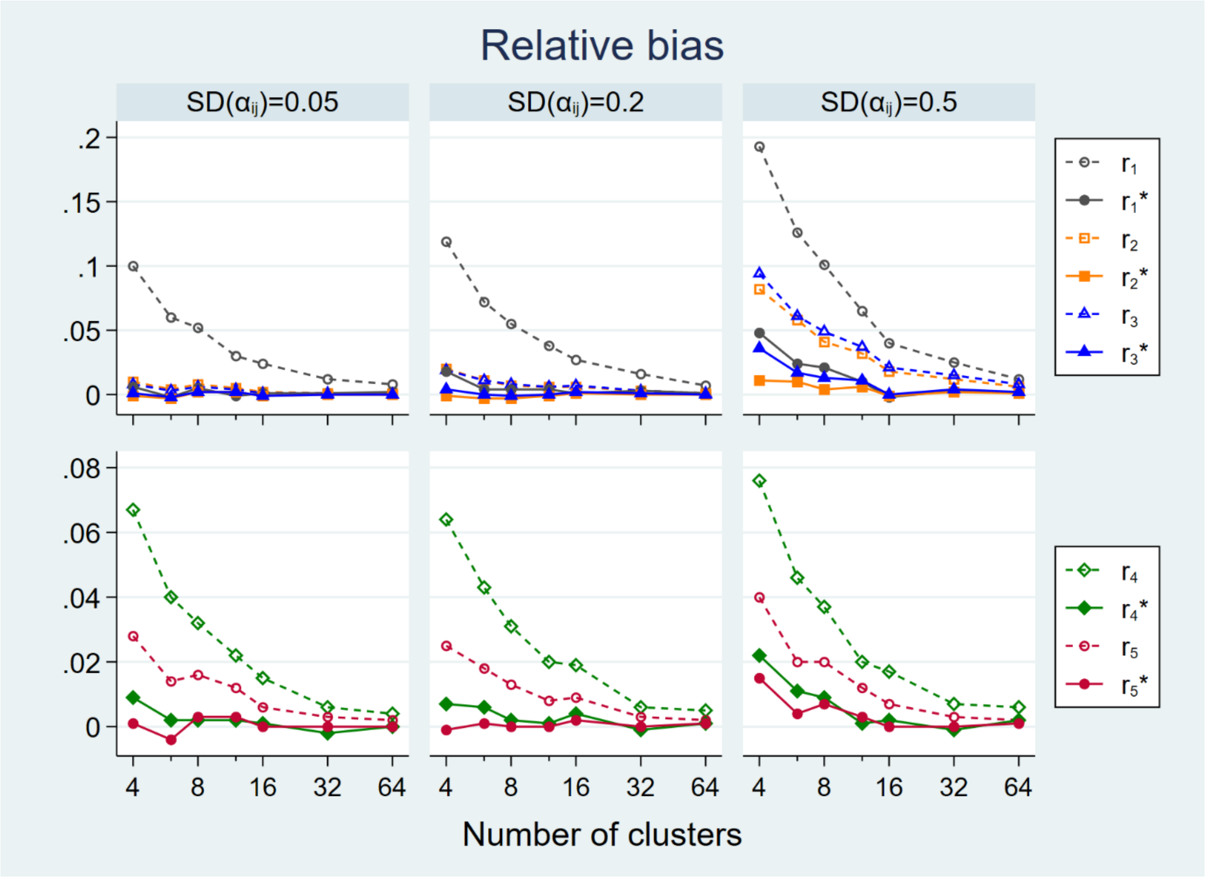

Figure S2. Relative bias of intervention effect estimators in relation to the number of clusters per trial arm for non-matched CRTs, by three levels of $SD(\alpha_{ij})$; population size per cluster follows a skewed distribution with mean = 100 and $CV = 0.6$; intervention has a direct effect only $(\exp(\beta_D) = 0.5;\ \exp(\beta_I) = 1)$.

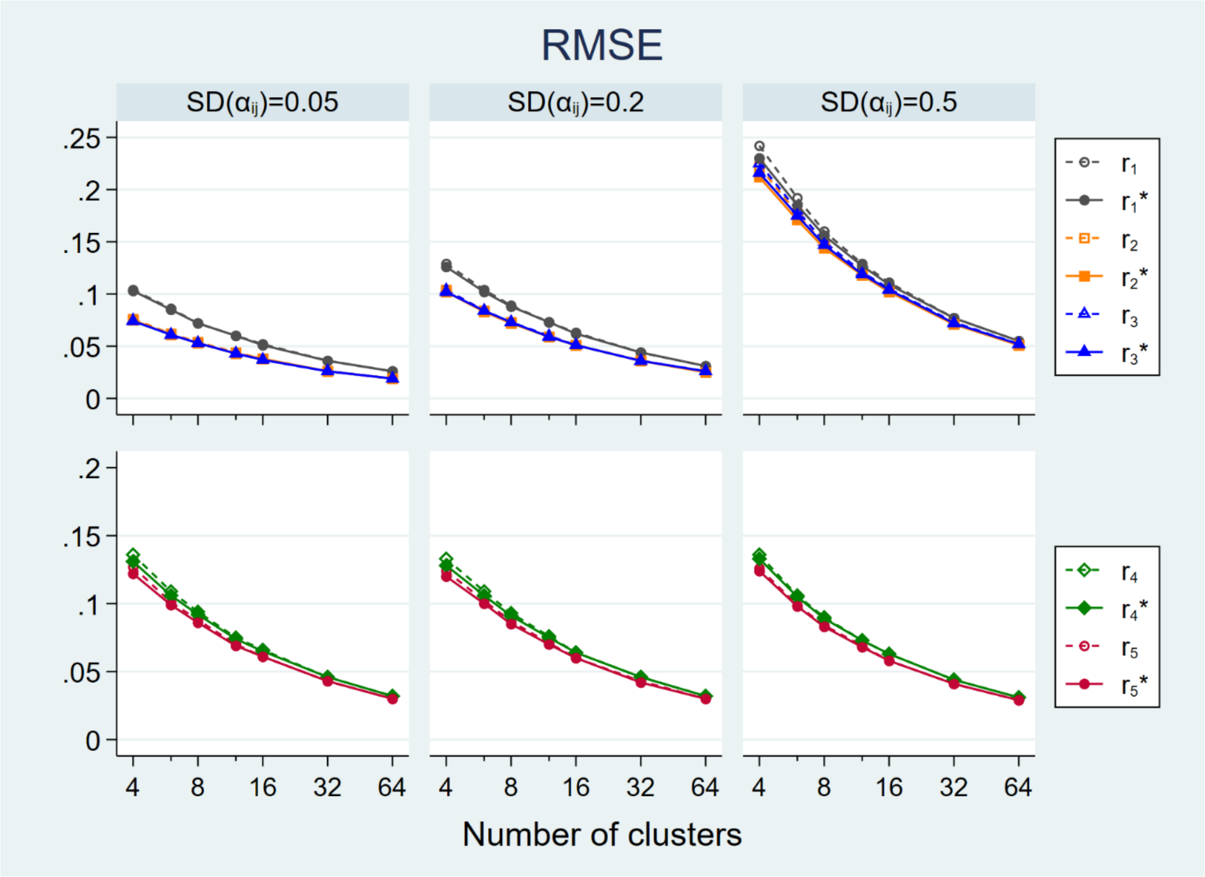

Figure S3. Root mean squared error (RMSE) of intervention effect estimators in relation to the number of clusters per trial arm for non-matched CRTs, by three levels of $SD(\alpha_{ij})$; population size per cluster follows a skewed distribution with mean = 100 and $CV = 0.2$; intervention has a direct effect only $(\exp(\beta_D) = 0.5;\ \exp(\beta_I) = 1)$.

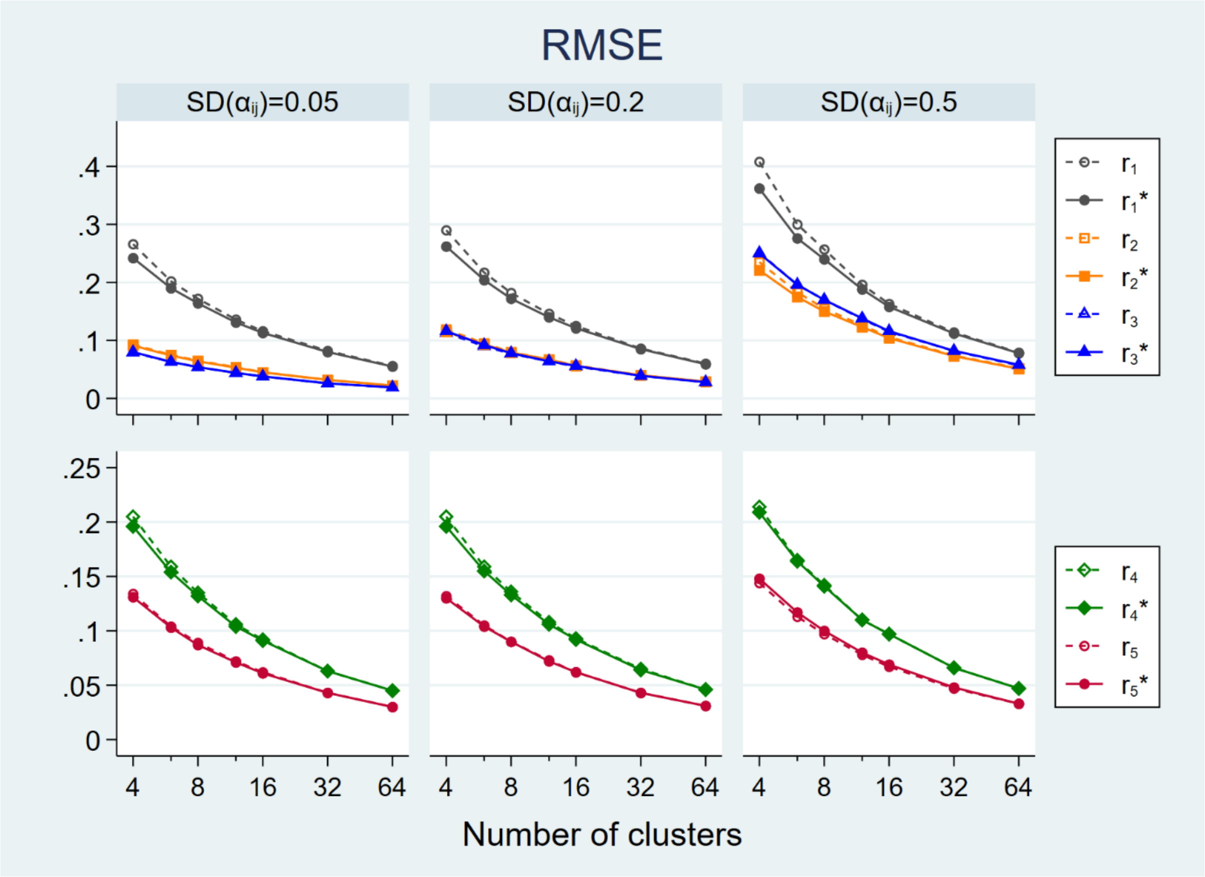

Figure S4. Root mean squared error (RMSE) of intervention effect estimators in relation to the number of clusters per trial arm for non-matched CRTs, by three levels of $SD(\alpha_{ij})$; population size per cluster follows a skewed distribution with mean = 100 and $CV = 0.6$; intervention has a direct effect only $(\exp(\beta_D) = 0.5;\ \exp(\beta_I) = 1)$.

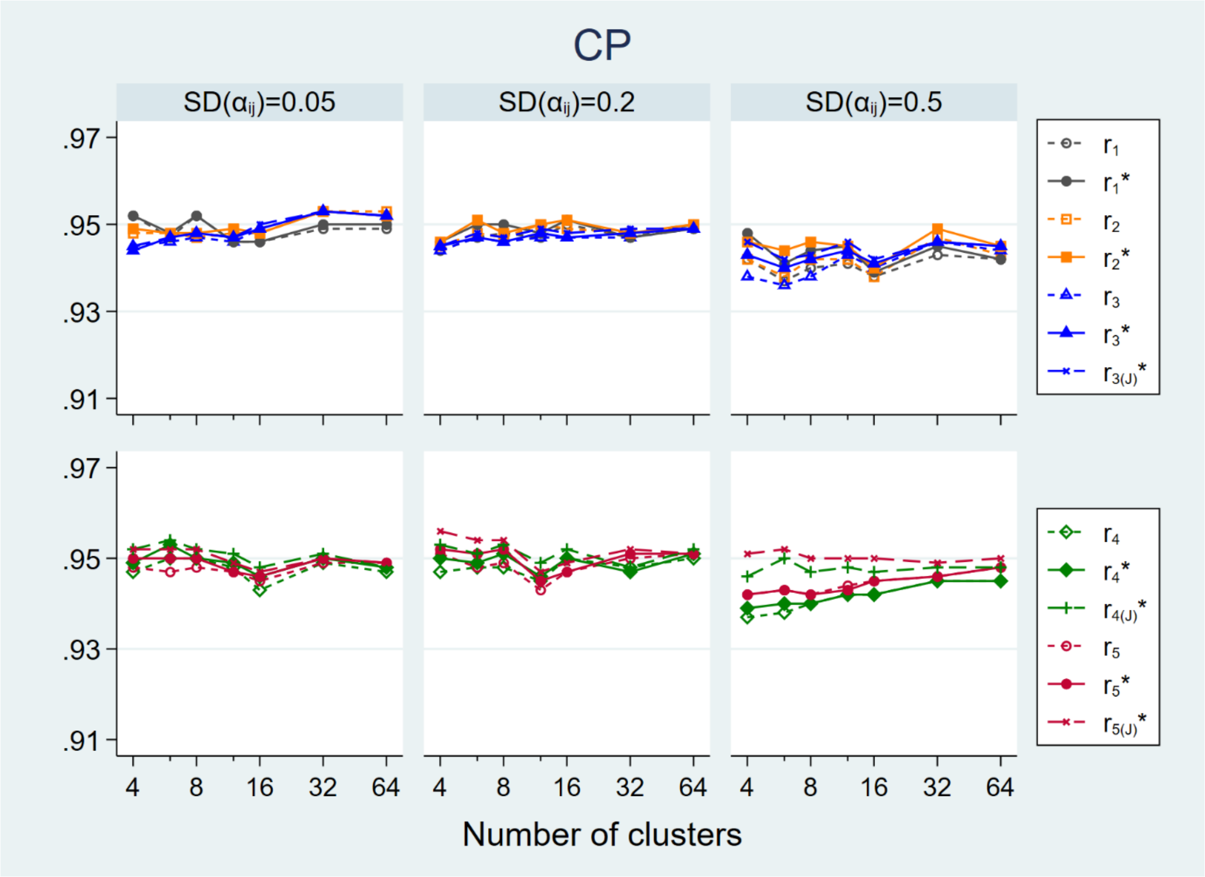

Figure S5. Coverage probability (CP) of 95% confidence interval (calculated on log-scale) in relation to the number of clusters per trial arm for non-matched CRTs, by three levels of $SD(\alpha_{ij})$; population size per cluster follows a skewed distribution with mean = 100 and $CV = 0.2$; intervention has a direct effect only ($\exp(\beta_D) = 0.5;\ \exp(\beta_I) = 1$).

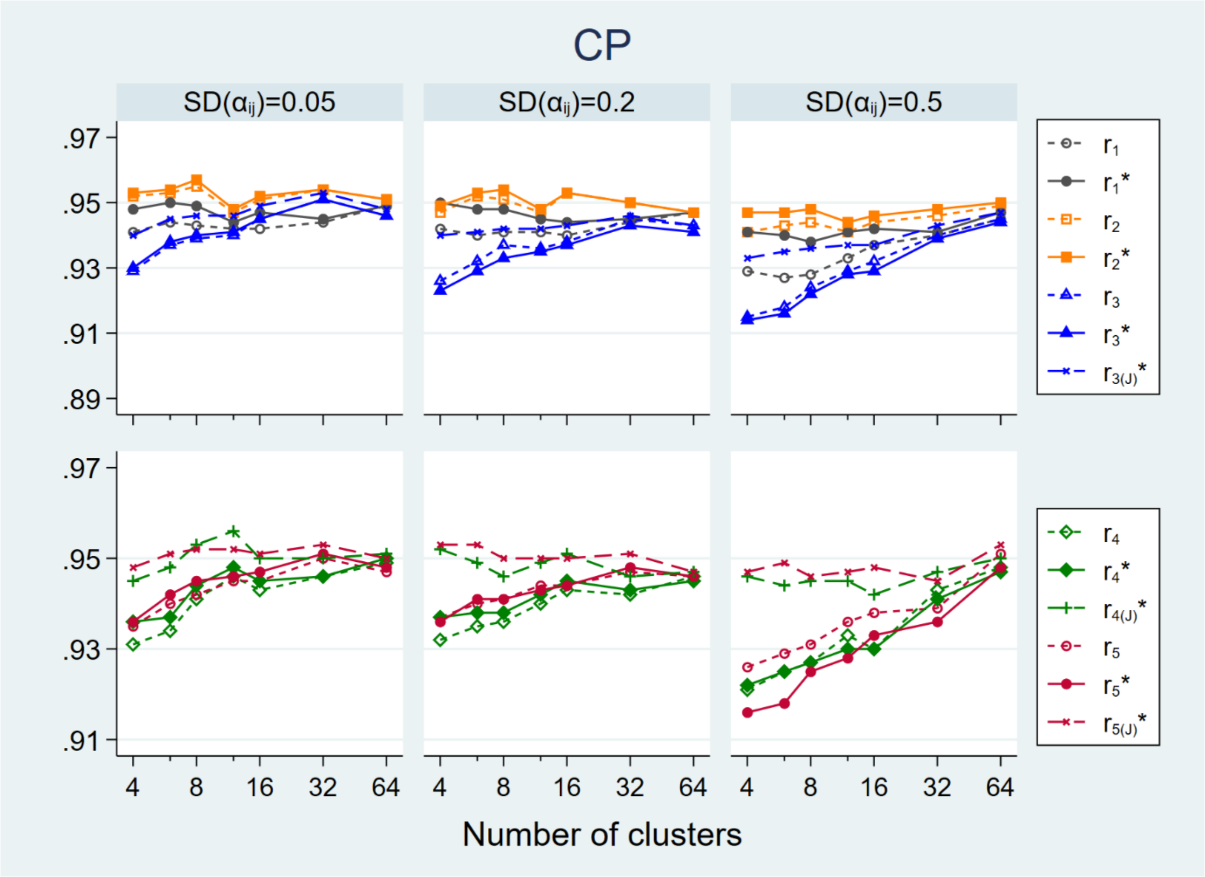

Figure S6. Coverage probability (CP) of 95% confidence interval (calculated on log-scale) of intervention effect estimators in relation to the number of clusters per trial arm for non-matched CRTs, by three levels of $SD(\alpha_{ij})$; population size per cluster follows a skewed distribution with mean = 100 and $CV = 0.6$; intervention has a direct effect only $(\exp(\beta_D) = 0.5;\ \exp(\beta_I) = 1)$.

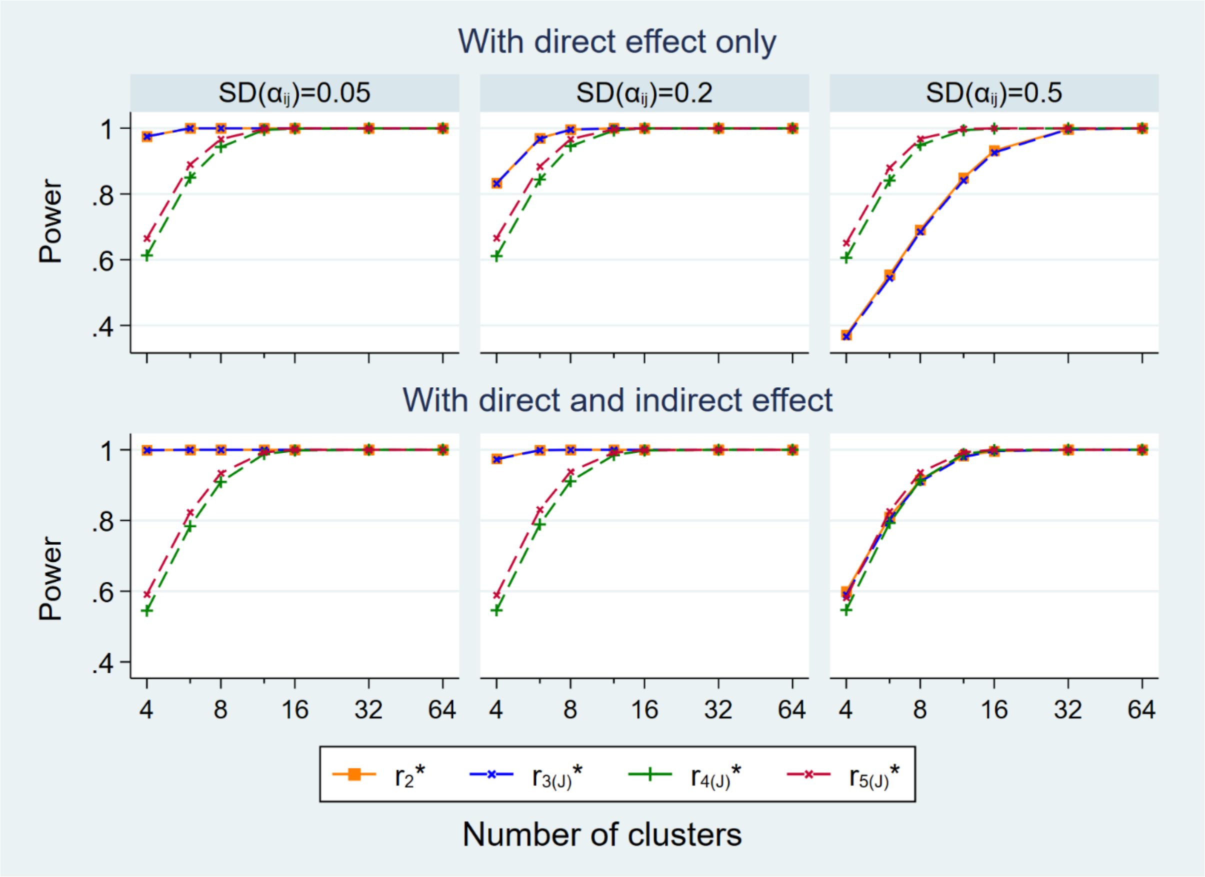

Figure S7. Power of $r_2^*, r_{3(J)}^*, r_{4(J)}^*$, and $r_{5(J)}^*$ in relation to the number of clusters per trial arm for non-matched CRTs, by three levels of $SD(\alpha_{ij})$; population size per cluster follows a skewed distribution with mean = 100 and $CV = 0.2$. Upper panel: with direct effect only $(\exp(\beta_D) = 0.5;\ \exp(\beta_I) = 1)$; lower panel: with direct and indirect effects $(\exp(\beta_D) = 0.5;\ \exp(\beta_I) = 0.75)$.

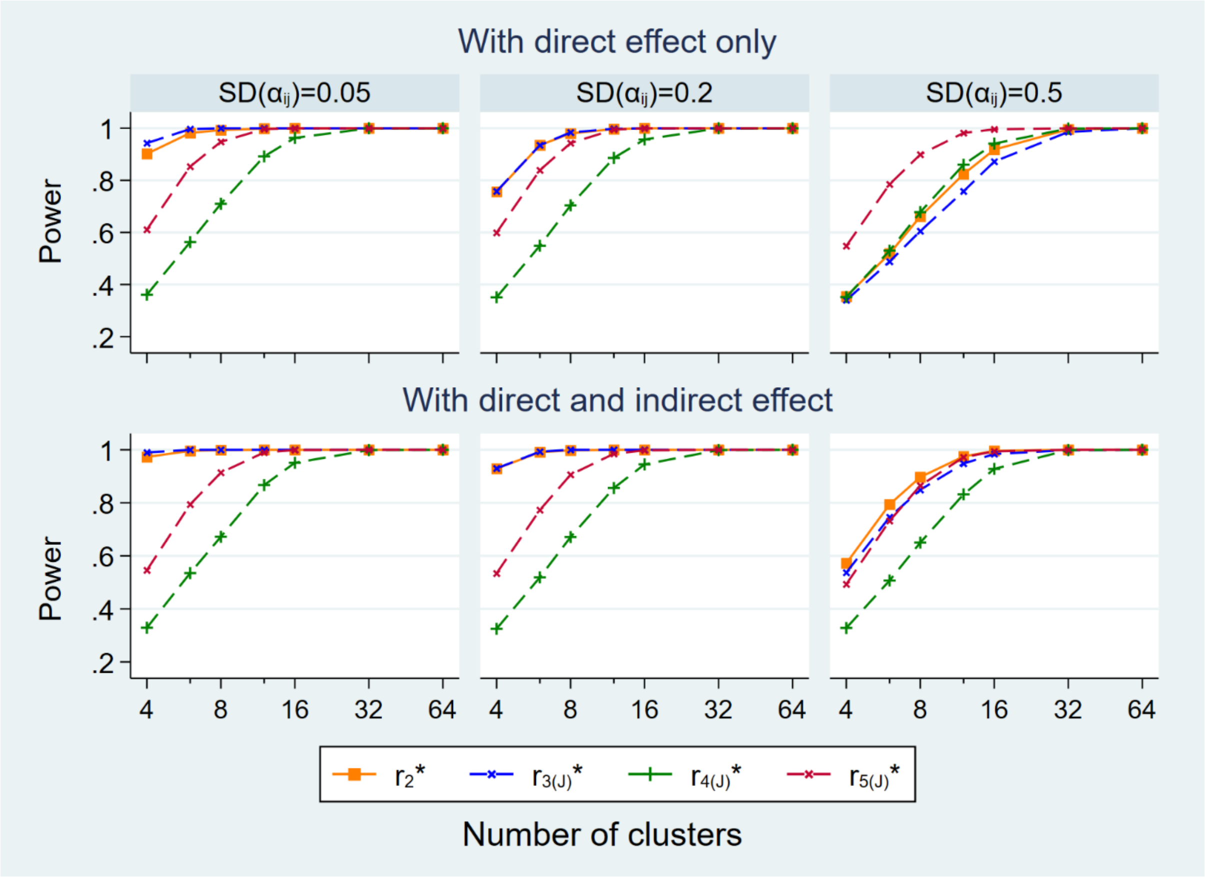

Figure S8. Power of $r_2^*, r_{3(J)}^*, r_{4(J)}^*$, and $r_{5(J)}^*$ in relation to the number of clusters per trial arm for non-matched CRTs, by three levels of $SD(\alpha_{ij})$; population size per cluster follows a skewed distribution with mean = 100 and $CV = 0.6$. Upper panel: with direct effect only ($\exp(\beta_D) = 0.5;\ \exp(\beta_I) = 1$); lower panel: with direct and indirect effects ($\exp(\beta_D) = 0.5;\ \exp(\beta_I) = 0.75$).

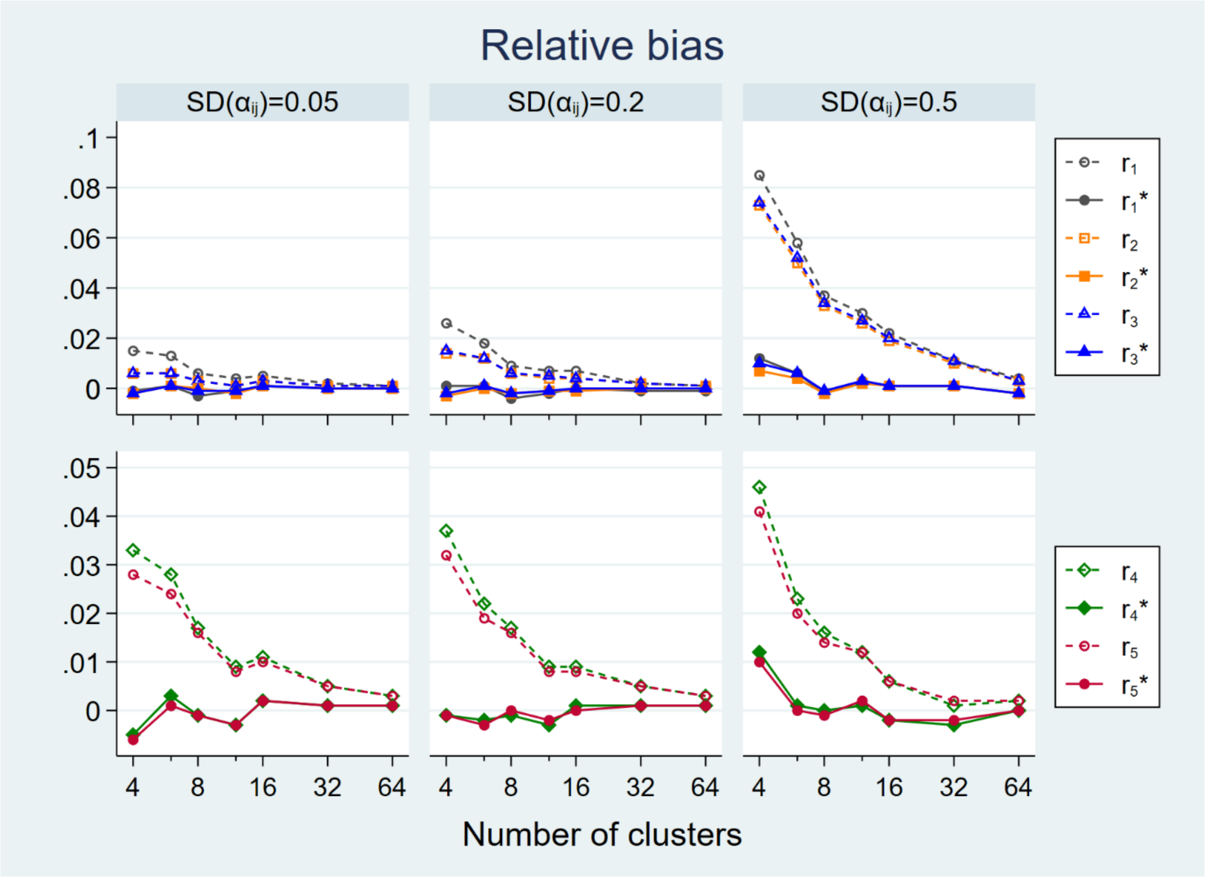

Figure S9. Relative bias of intervention effect estimators in relation to the number of clusters per trial arm for non-matched CRTs, by three levels of $SD(\alpha_{ij})$; population size per cluster follows a skewed distribution with mean = 100 and $CV = 0.2$; intervention has direct and indirect effects $(\exp(\beta_D) = 0.5;\ \exp(\beta_I) = 0.75)$.

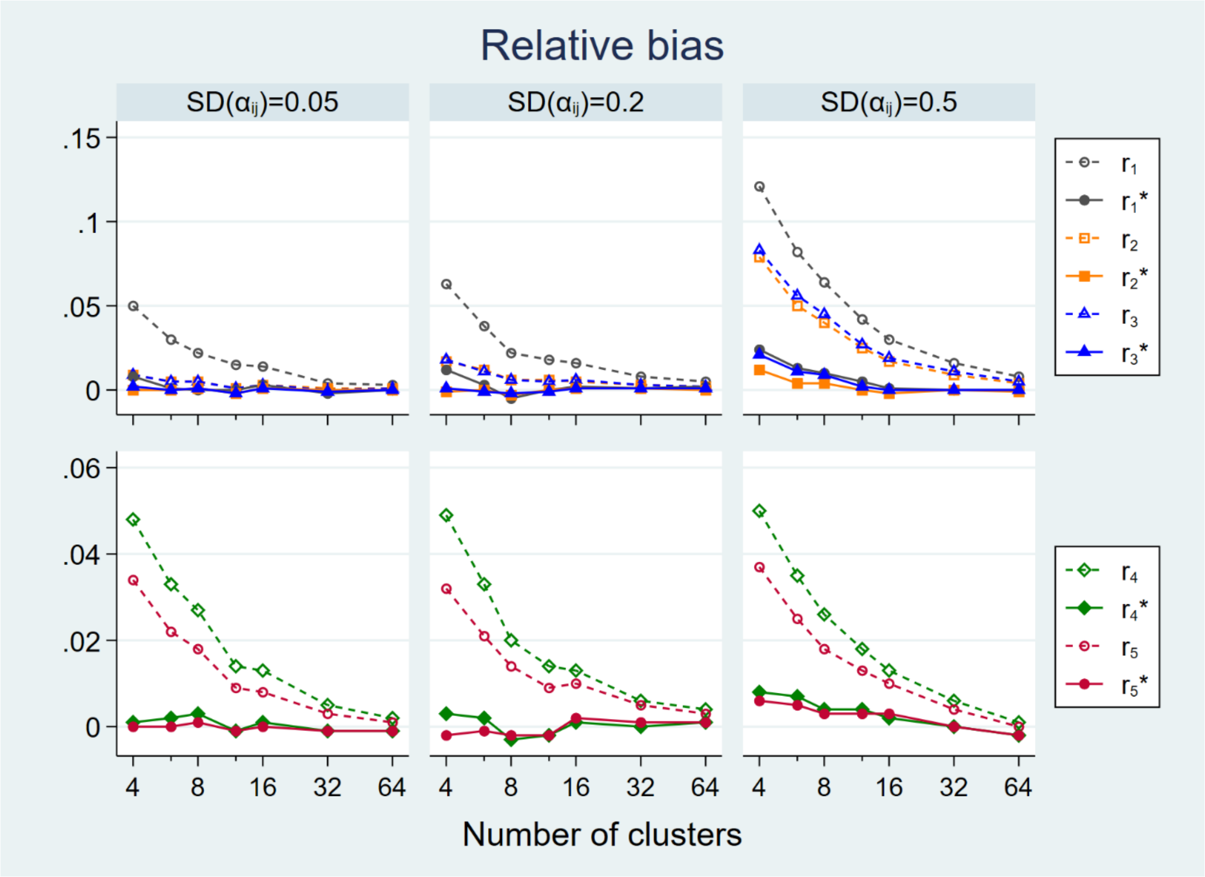

Figure S10. Relative bias of intervention effect estimators in relation to the number of clusters per trial arm for non-matched CRTs, by three levels of $SD(\alpha_{ij})$; population size per cluster follows a skewed distribution with mean = 100 and $CV = 0.4$; intervention has direct and indirect effects $(\exp(\beta_D) = 0.5;\ \exp(\beta_I) = 0.75)$.

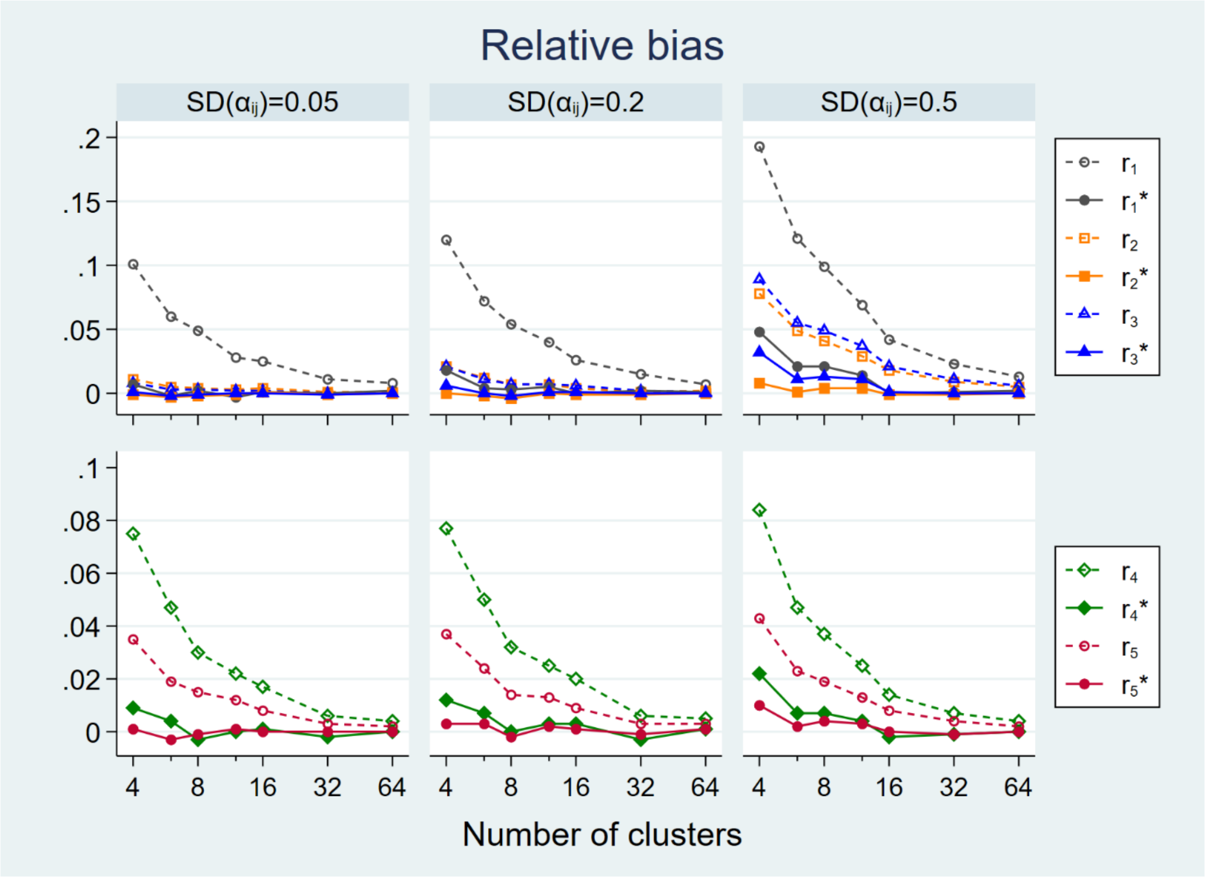

Figure S11. Relative bias of intervention effect estimators in relation to the number of clusters per trial arm for non-matched CRTs, by three levels of $SD(\alpha_{ij})$; population size per cluster follows a skewed distribution with mean = 100 and $CV = 0.6$; intervention has direct and indirect effects $(\exp(\beta_D) = 0.5;\ \exp(\beta_I) = 0.75)$.

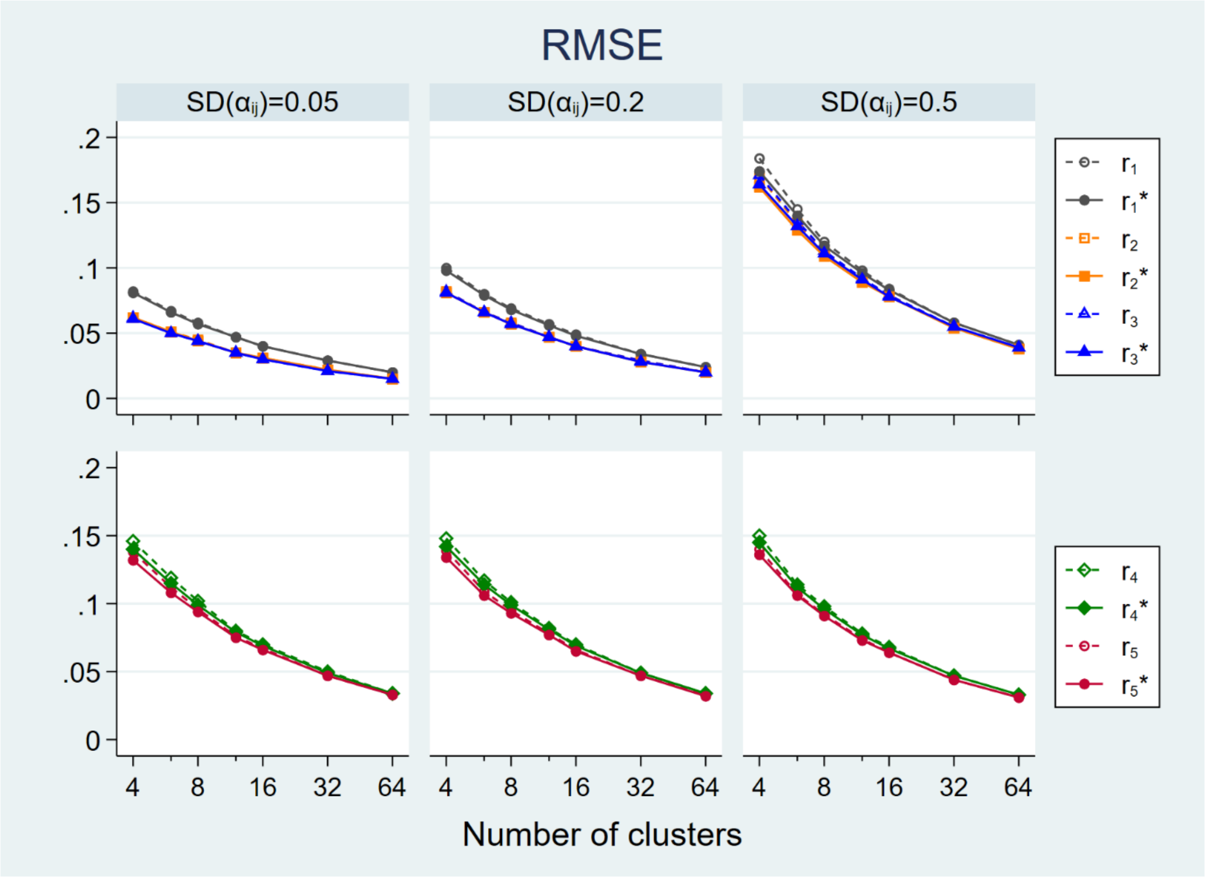

Figure S12. Root mean squared error (RMSE) of intervention effect estimators in relation to the number of clusters per trial arm for non-matched CRTs, by three levels of $SD(\alpha_{ij})$; population size per cluster follows a skewed distribution with mean = 100 and $CV = 0.2$; intervention has direct and indirect effects $(\exp(\beta_D) = 0.5;\ \exp(\beta_I) = 0.75)$.

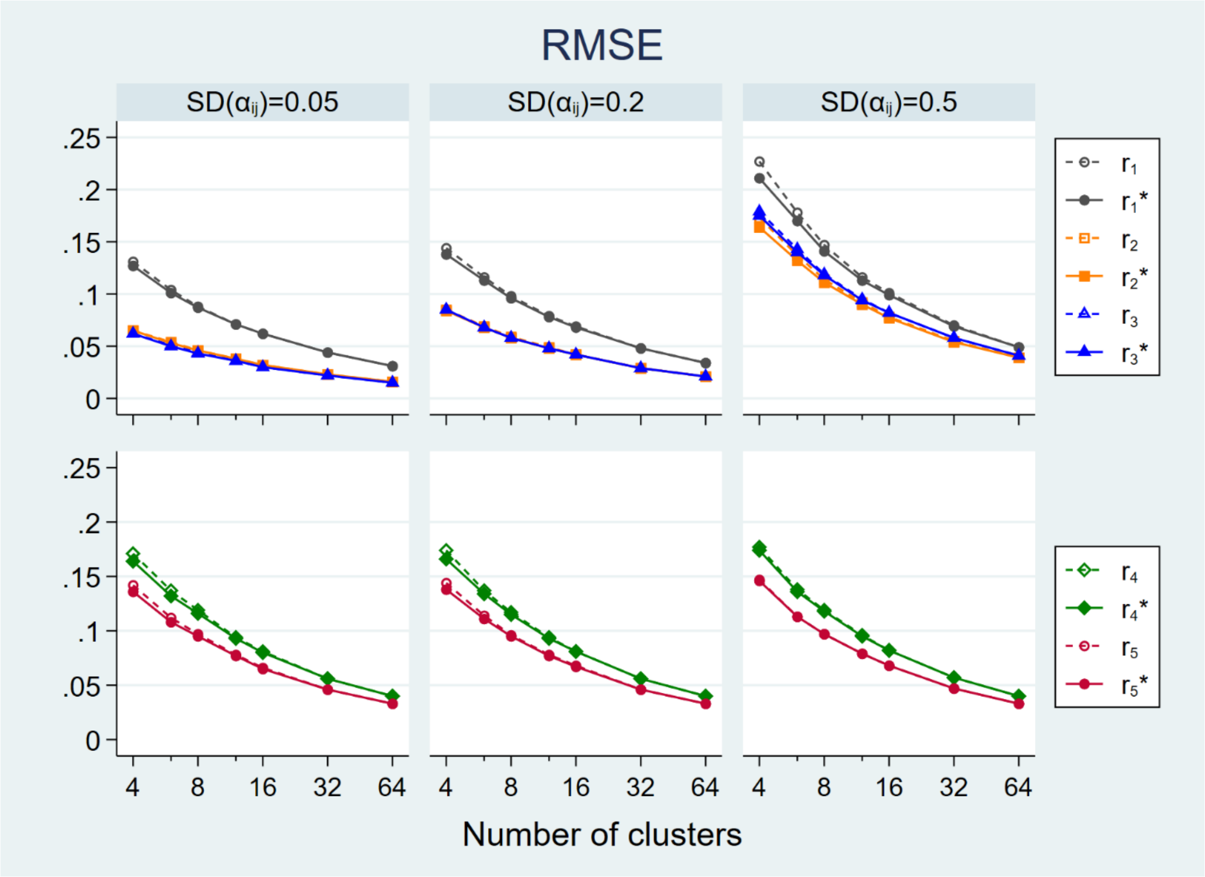

Figure S13. Root mean squared error (RMSE) of intervention effect estimators in relation to the number of clusters per trial arm for non-matched CRTs, by three levels of $SD(\alpha_{ij})$; population size per cluster follows a skewed distribution with mean = 100 and $CV = 0.4$; intervention has direct and indirect effects $(\exp(\beta_D) = 0.5;\ \exp(\beta_I) = 0.75)$.

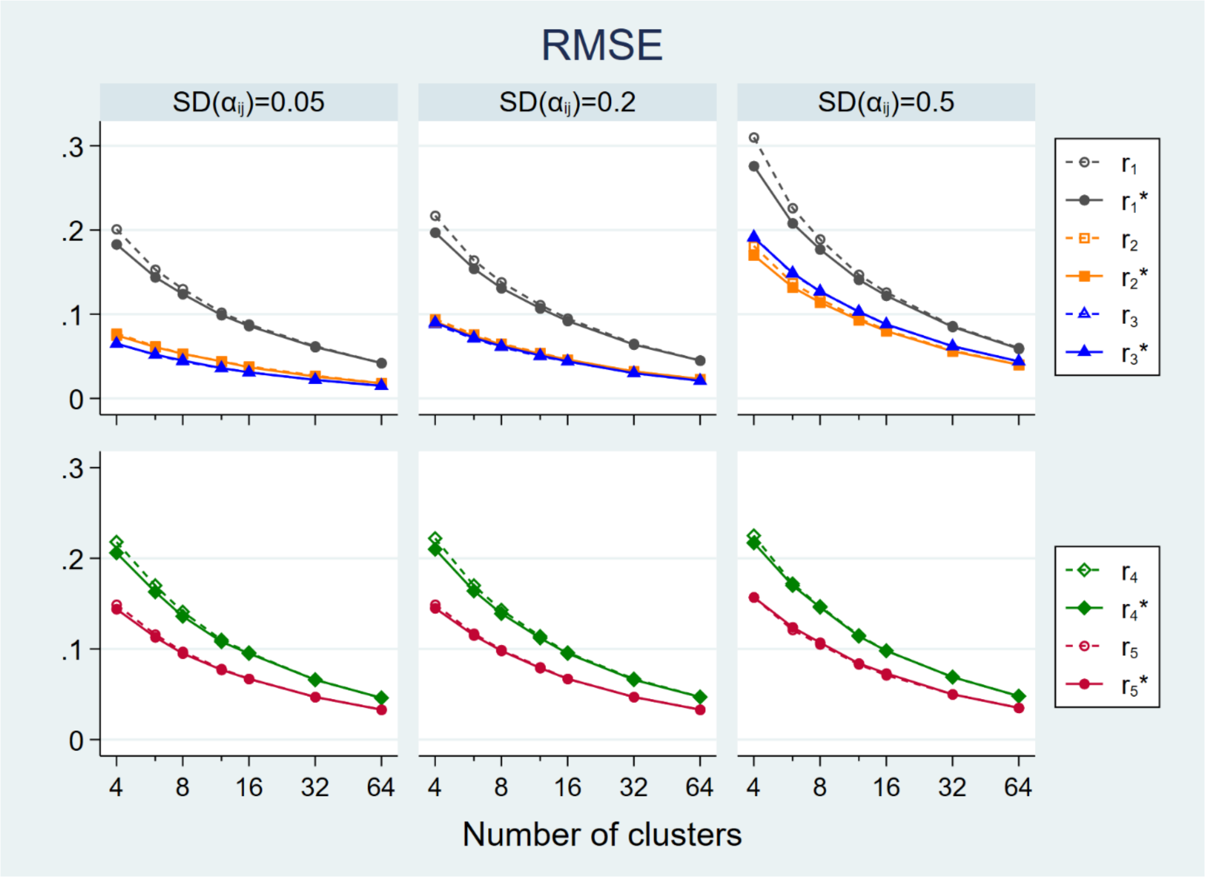

Figure S14. Root mean squared error (RMSE) of intervention effect estimators in relation to the number of clusters per trial arm for non-matched CRTs, by three levels of $SD(\alpha_{ij})$; population size per cluster follows a skewed distribution with mean = 100 and $CV = 0.6$; intervention has direct and indirect effects $(\exp(\beta_D) = 0.5;\ \exp(\beta_I) = 0.75)$.

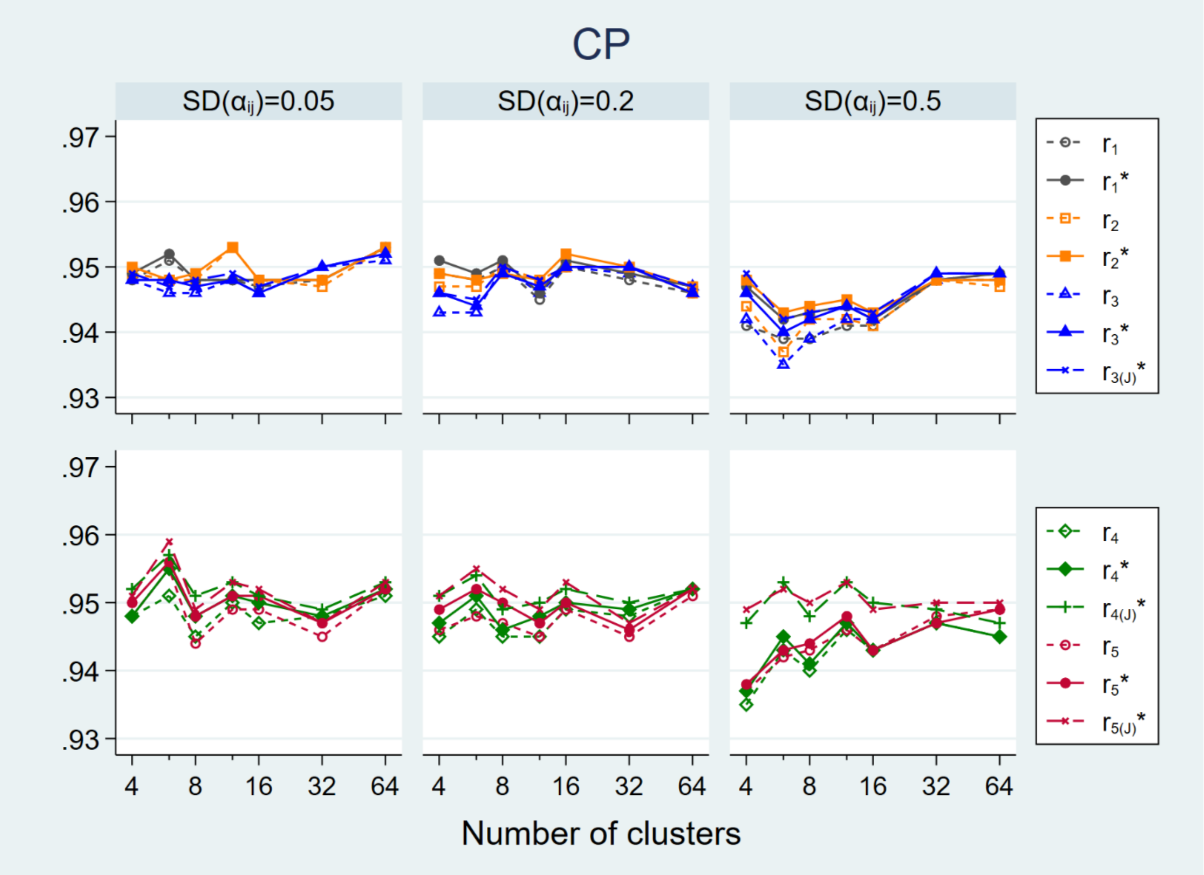

Figure S15. Coverage probability (CP) of 95% confidence interval (calculated on log-scale) of intervention effect estimators in relation to the number of clusters per trial arm for non-matched CRTs, by three levels of $SD(\alpha_{ij})$; population size per cluster follows a skewed distribution with mean = 100 and $CV = 0.2$; intervention has direct and indirect effects $(\exp(\beta_D) = 0.5;\ \exp(\beta_I) = 0.75)$.

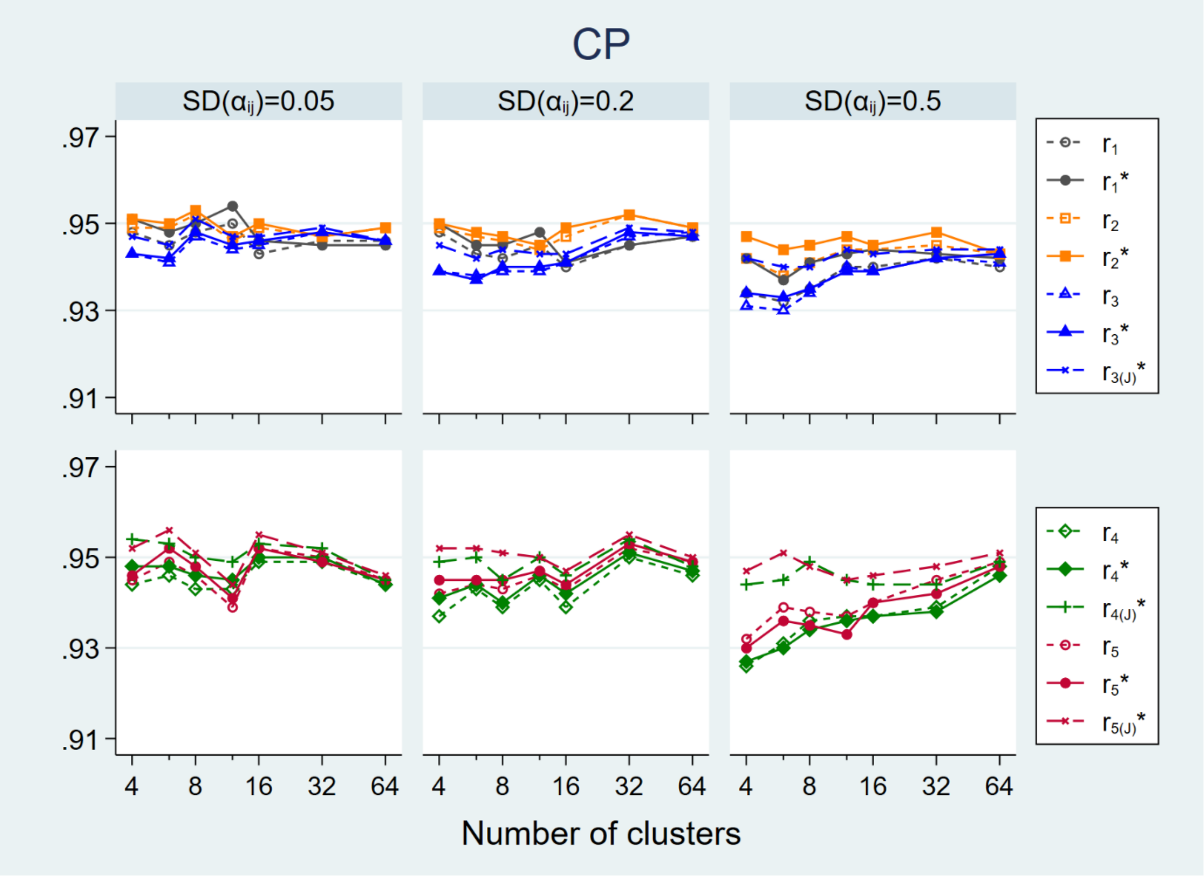

Figure S16. Coverage probability (CP) of 95% confidence interval (calculated on log-scale) of intervention effect estimators in relation to the number of clusters per trial arm for non-matched CRTs, by three levels of $SD(\alpha_{ij})$, population size per cluster follows a skewed distribution with mean = 100 and $CV = 0.4$; intervention has direct and indirect effects ($\exp(\beta_D) = 0.5;\ \exp(\beta_I) = 0.75$).

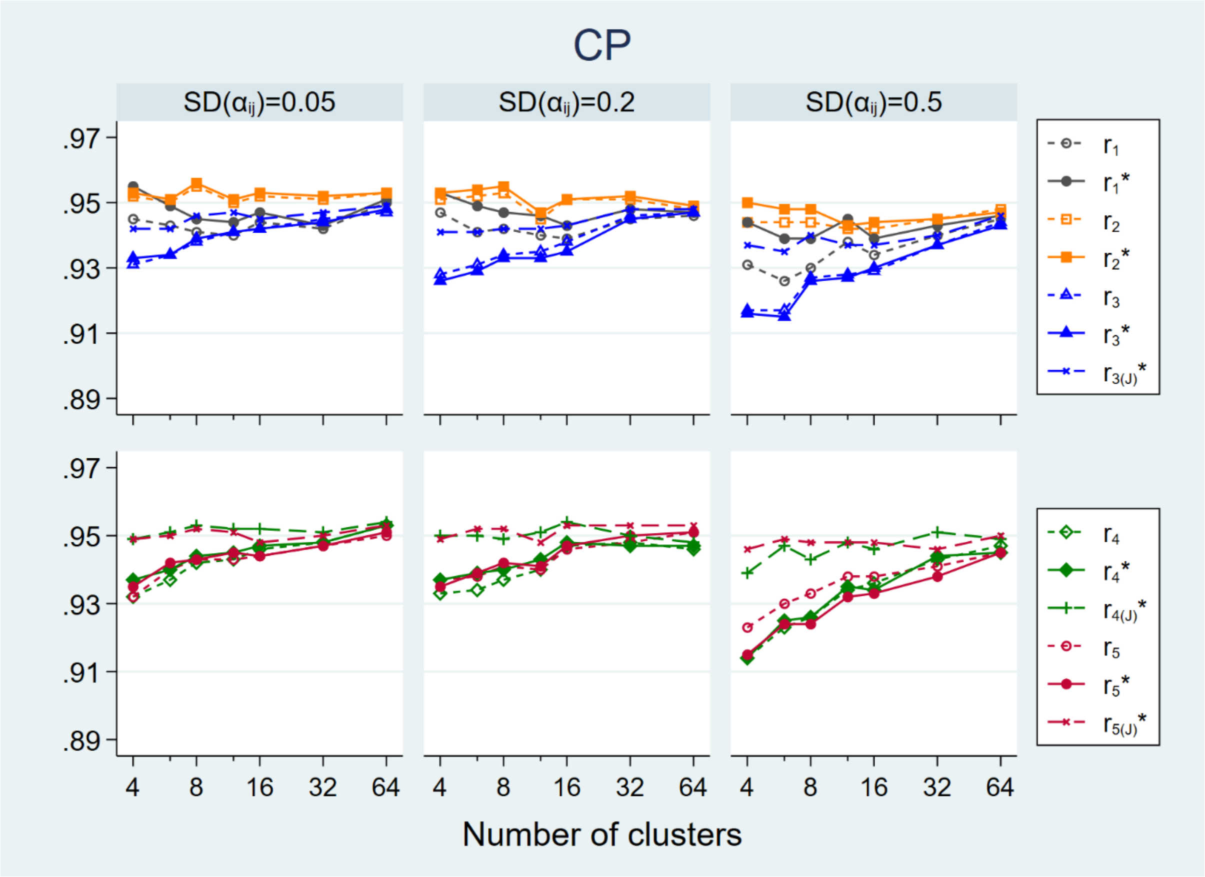

Figure S17. Coverage probability (CP) of 95% confidence interval (calculated on log-scale) of intervention effect estimators in relation to the number of clusters per trial arm for non-matched CRTs, by three levels of $SD(\alpha_{ij})$; population size per cluster follows a skewed distribution with mean = 100 and $CV = 0.6$; intervention has direct and indirect effects $(\exp(\beta_D) = 0.5;\ \exp(\beta_I) = 0.75)$.

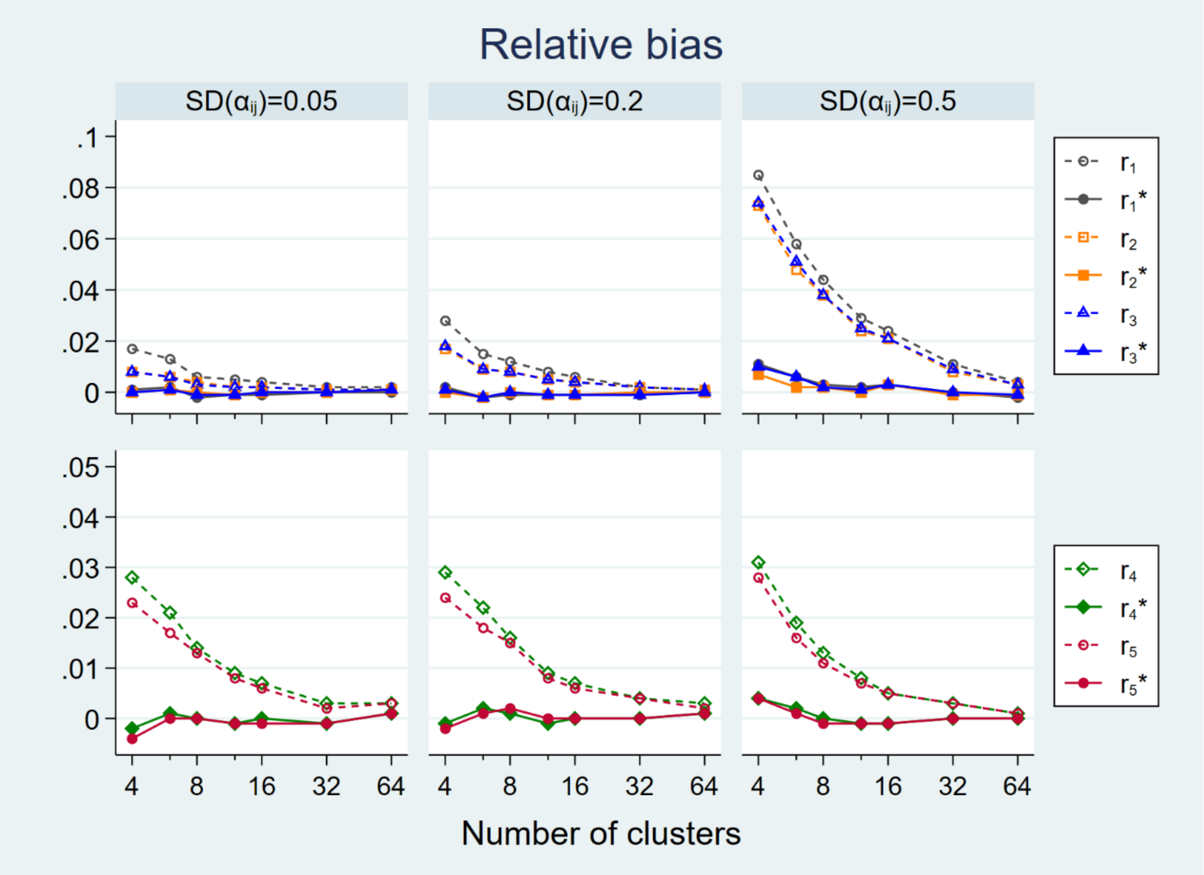

Figure S18. Relative bias of intervention effect estimators in relation to the number of clusters per trial arm for non-matched CRTs, by three levels of $SD(\alpha_{ij})$; population size per cluster follows a skewed distribution with mean = 100 and $CV = 0.2$; intervention has no effect ($\exp(\beta_D) = 1;\ \exp(\beta_I) = 1$).

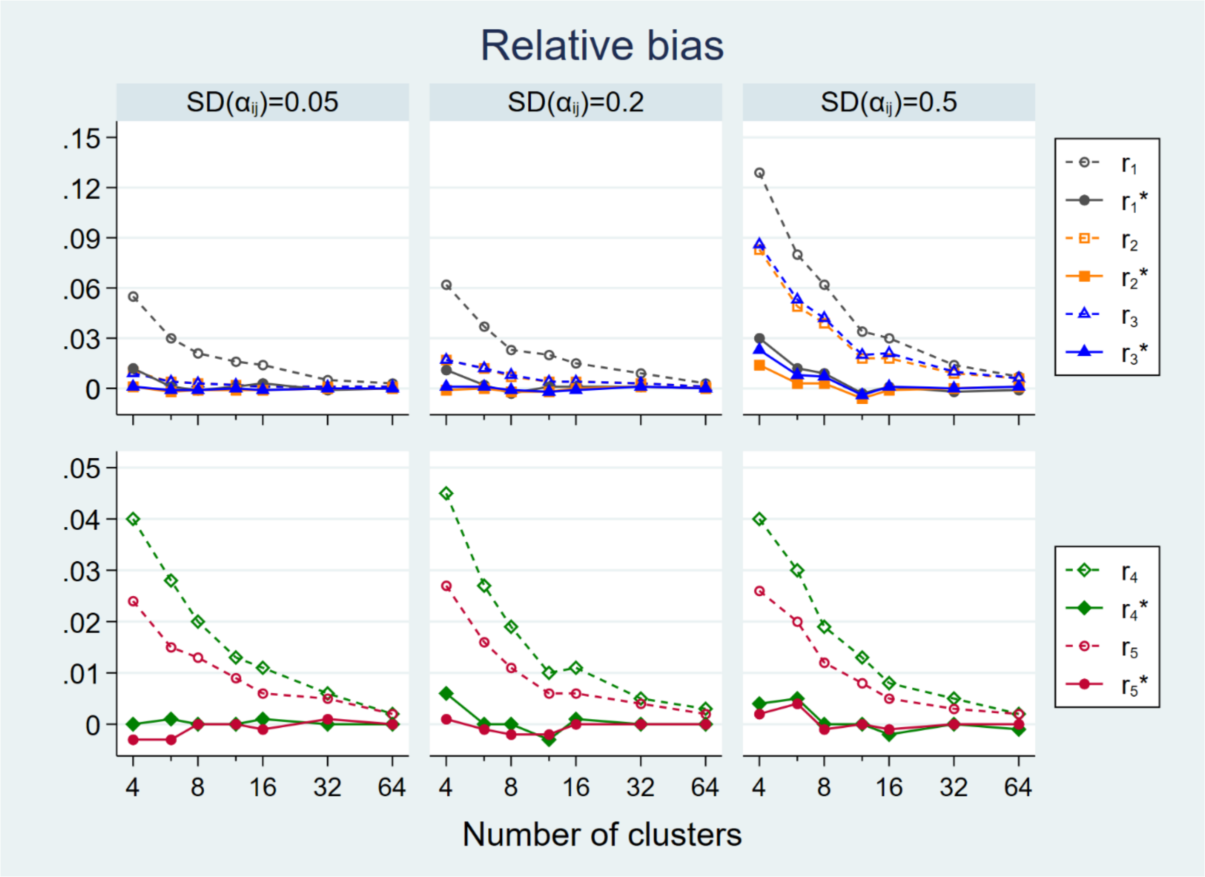

Figure S19. Relative bias of intervention effect estimators in relation to the number of clusters per trial arm for non-matched CRTs, by three levels of $SD(\alpha_{ij})$; population size per cluster follows a skewed distribution with mean = 100 and $CV = 0.4$; intervention has no effect ($\exp(\beta_D) = 1;\ \exp(\beta_I) = 1$).

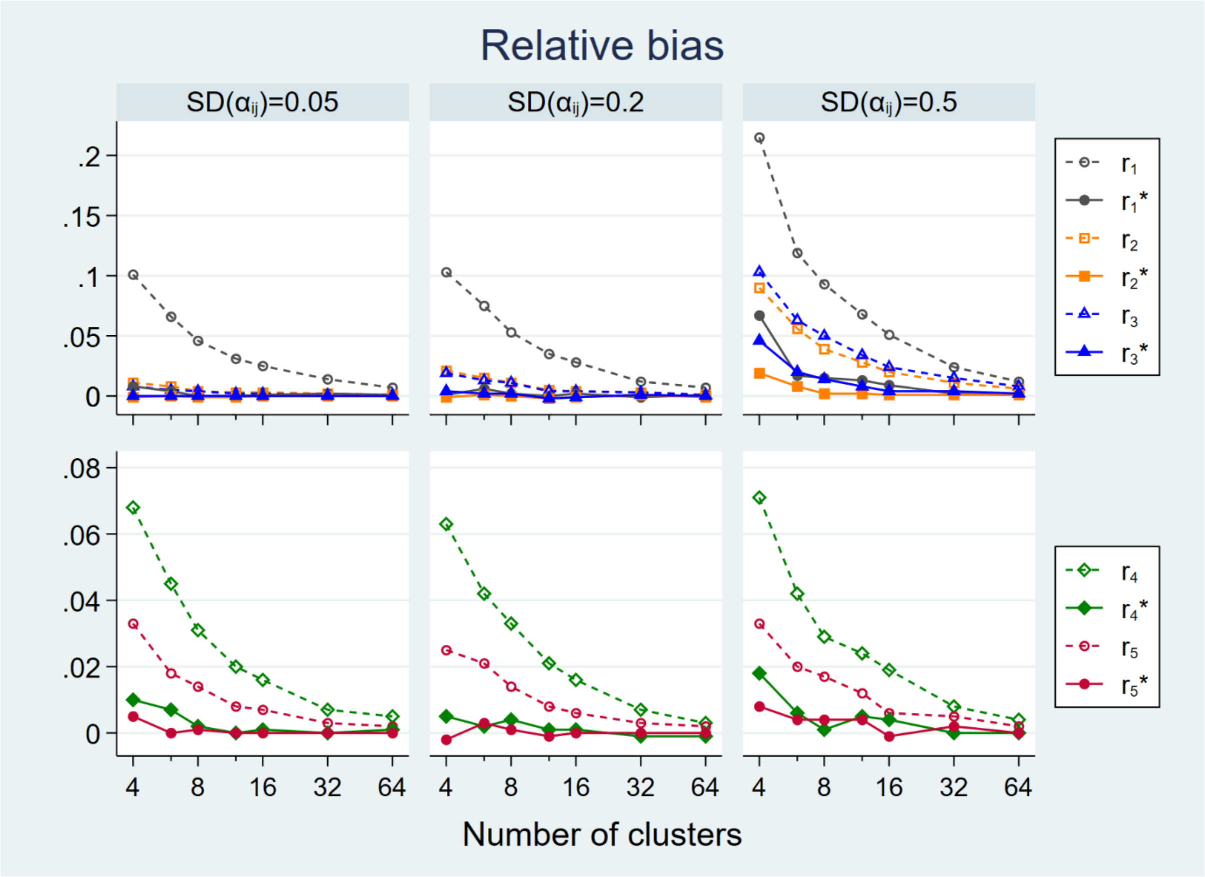

Figure S20. Relative bias of intervention effect estimators in relation to the number of clusters per trial arm for non-matched CRTs, by three levels of $SD(\alpha_{ij})$; population size per cluster follows a skewed distribution with mean = 100 and $CV = 0.6$; intervention has no effect ($\exp(\beta_D) = 1;\ \exp(\beta_I) = 1$).

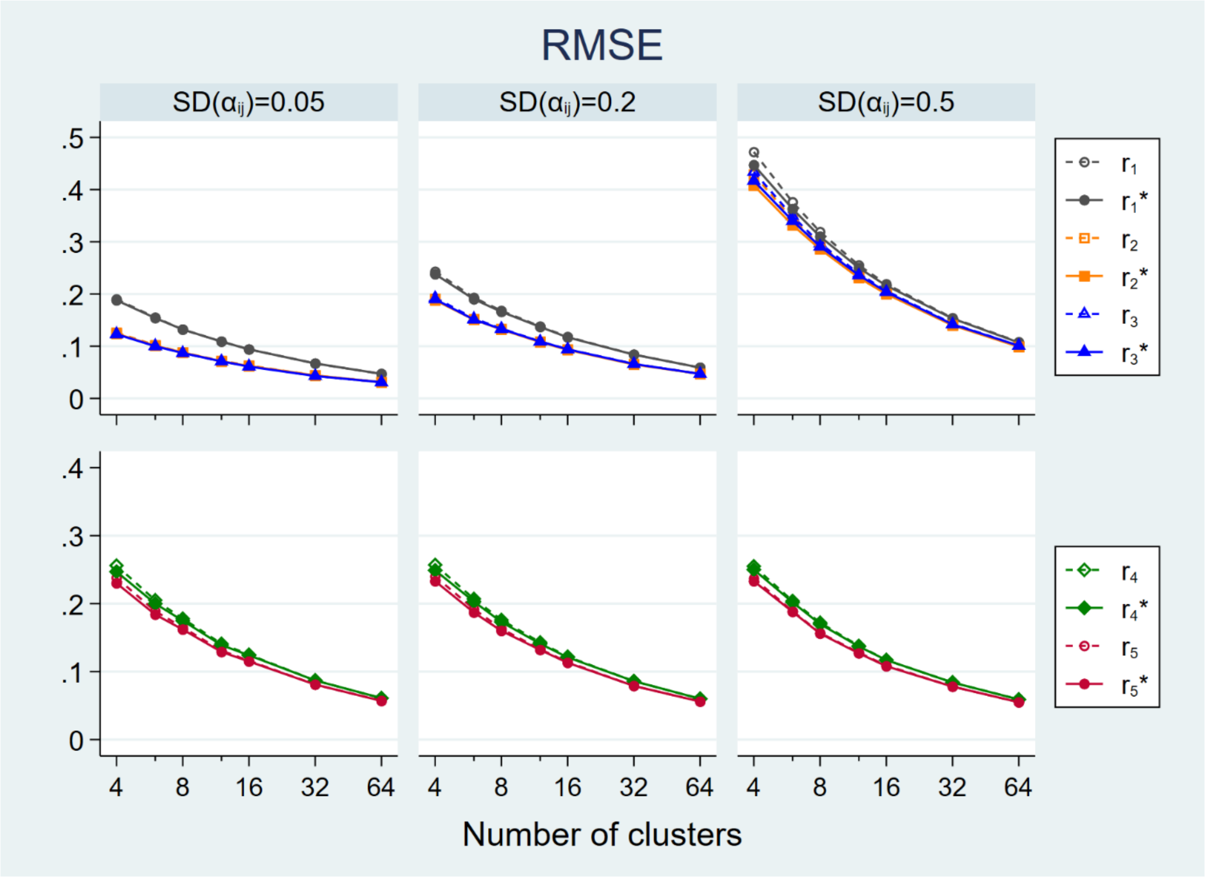

Figure S21. Root mean squared error (RMSE) of intervention effect estimators in relation to the number of clusters per trial arm for non-matched CRTs, by three levels of $SD(\alpha_{ij})$; population size per cluster follows a skewed distribution with mean = 100 and $CV = 0.2$; intervention has no effect $(\exp(\beta_D) = 1;\ \exp(\beta_I) = 1)$.

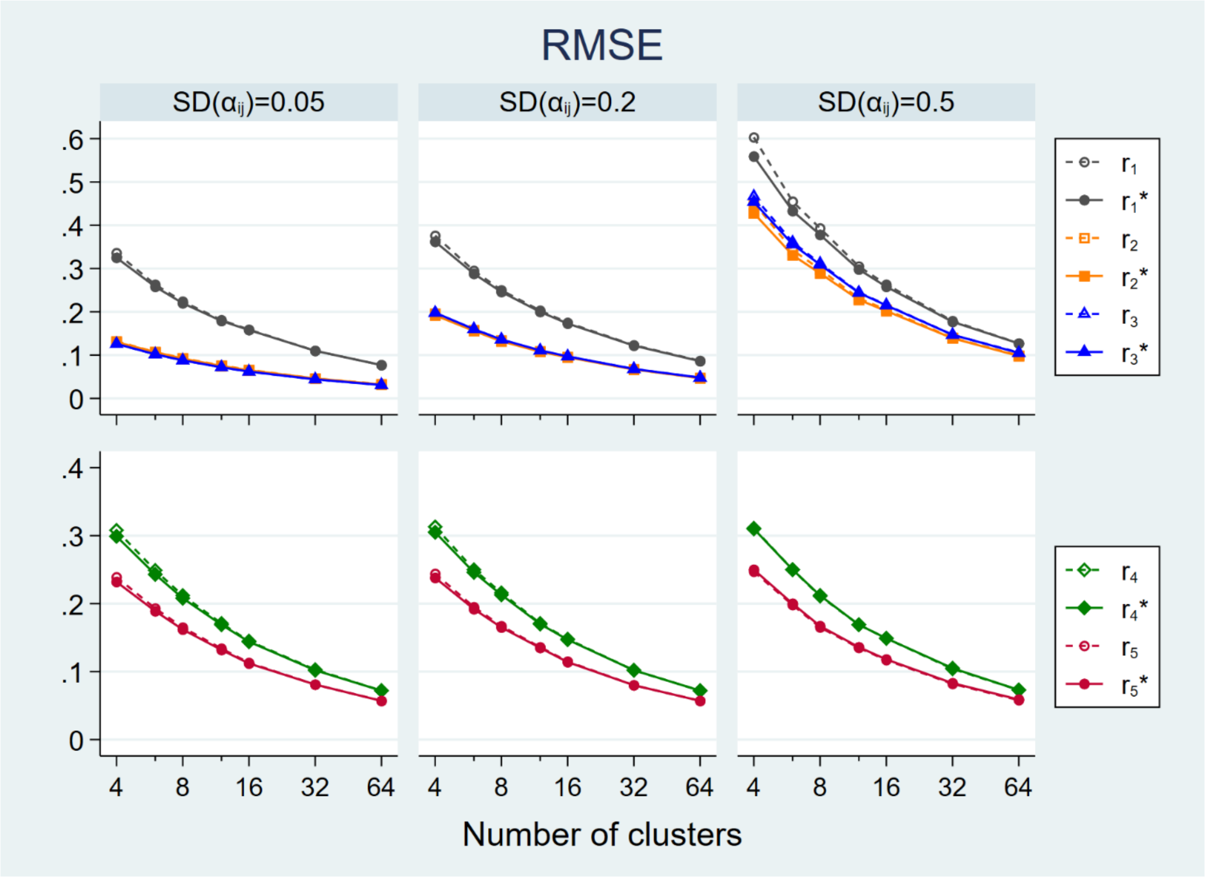

Figure S22. Root mean squared error (RMSE) of intervention effect estimators in relation to the number of clusters per trial arm for non-matched CRTs, by three levels of $SD(\alpha_{ij})$; population size per cluster follows a skewed distribution with mean = 100 and $CV = 0.4$; intervention has no effect ($\exp(\beta_D) = 1;\ \exp(\beta_I) = 1$).

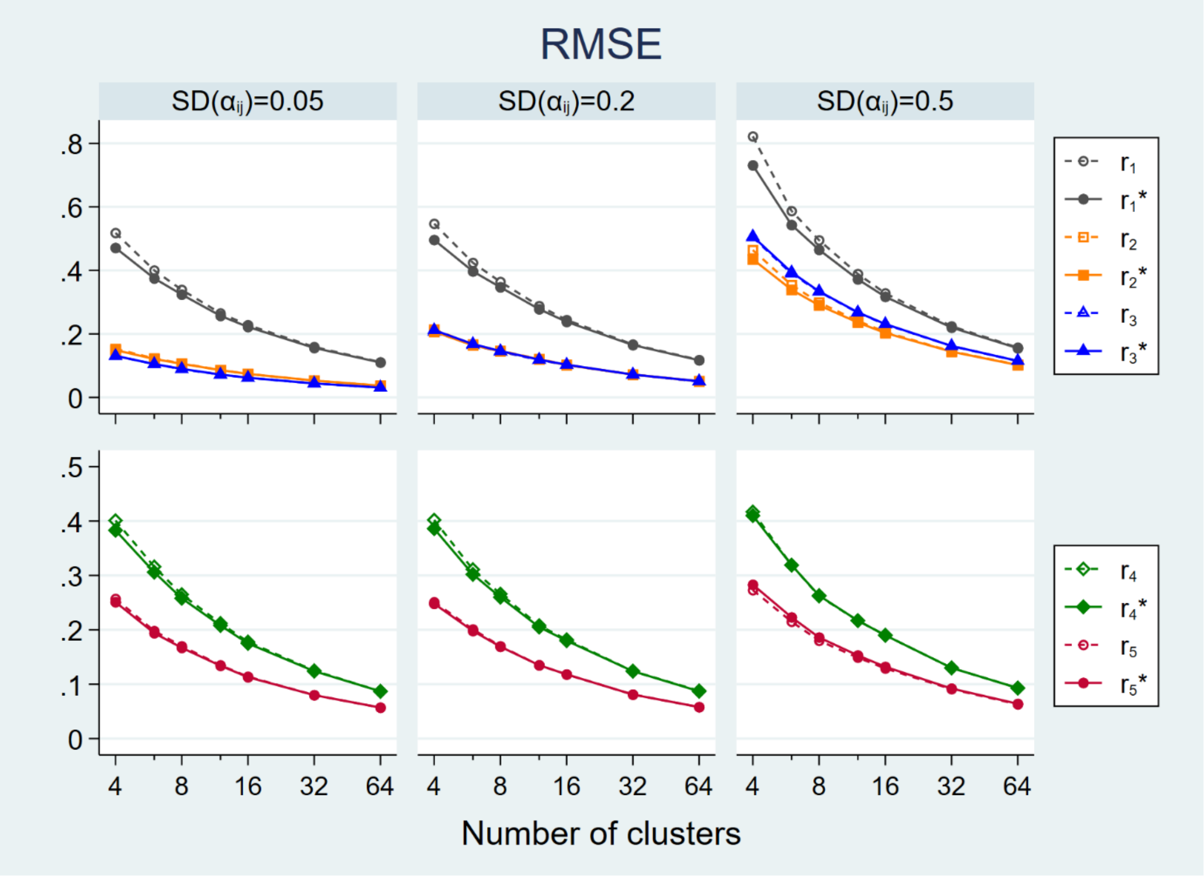

Figure S23. Root mean squared error (RMSE) of intervention effect estimators in relation to the number of clusters per trial arm for non-matched CRTs, by three levels of $SD(\alpha_{ij})$; population size per cluster follows a skewed distribution with mean = 100 and $CV = 0.6$; intervention has no effect ($\exp(\beta_D) = 1;\ \exp(\beta_I) = 1$).

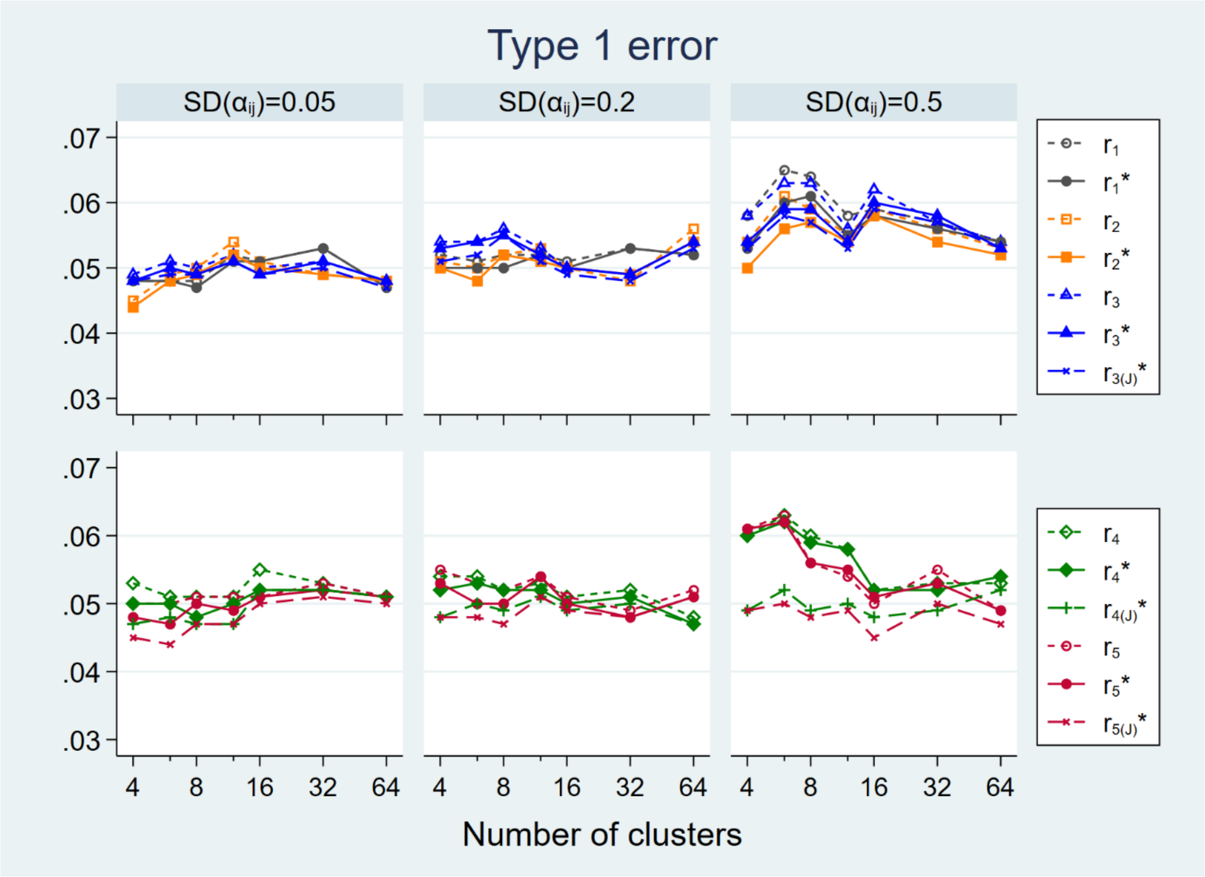

Figure S24. Type 1 error rate in relation to the number of clusters per trial arm for non-matched CRTs, by three levels of $SD(\alpha_{ij})$; population size per cluster follows a skewed distribution with mean = 100 and $CV = 0.2$; intervention has no effect $(\exp(\beta_D) = 1;\ \exp(\beta_I) = 1)$.

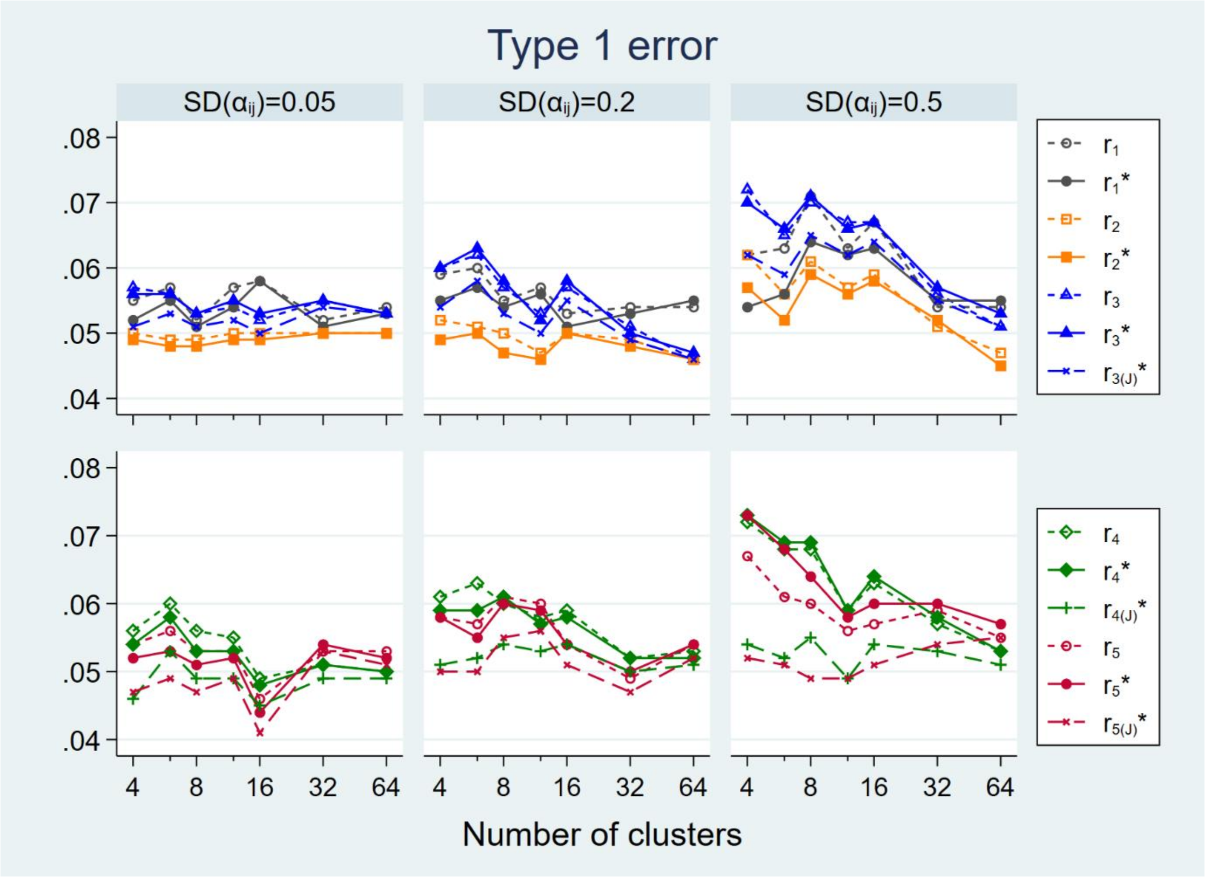

Figure S25. Type 1 error rate in relation to the number of clusters per trial arm for non-matched CRTs, by three levels of $SD(\alpha_{ij})$; population size per cluster follows a skewed distribution with mean = 100 and $CV = 0.4$; intervention has no effect $(\exp(\beta_D) = 1;\ \exp(\beta_I) = 1)$.

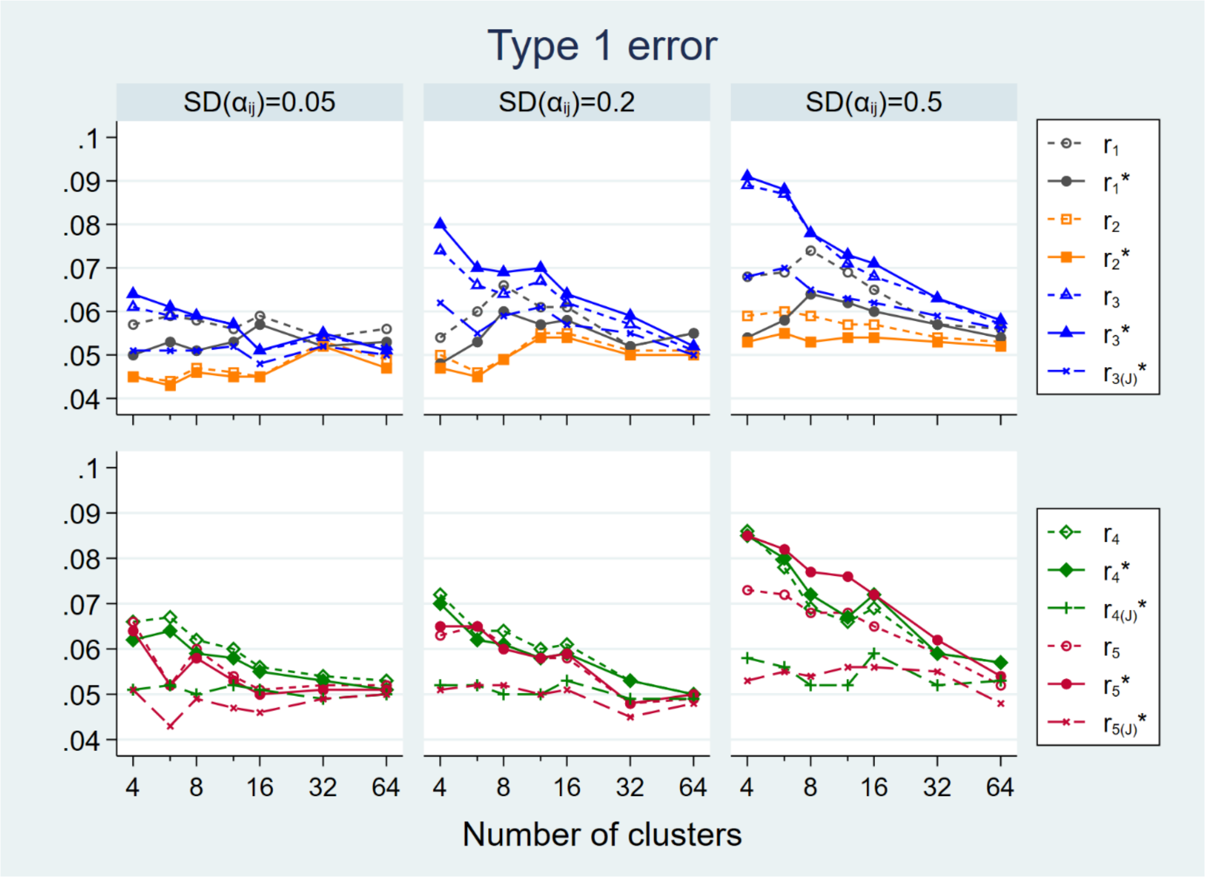

Figure S26. Type 1 error rate in relation to the number of clusters per trial arm for non-matched CRTs, by three levels of $SD(\alpha_{ij})$; population size per cluster follows a skewed distribution with mean = 100 and $CV = 0.6$; intervention has no effect ($\exp(\beta_D) = 1;\ \exp(\beta_I) = 1$).

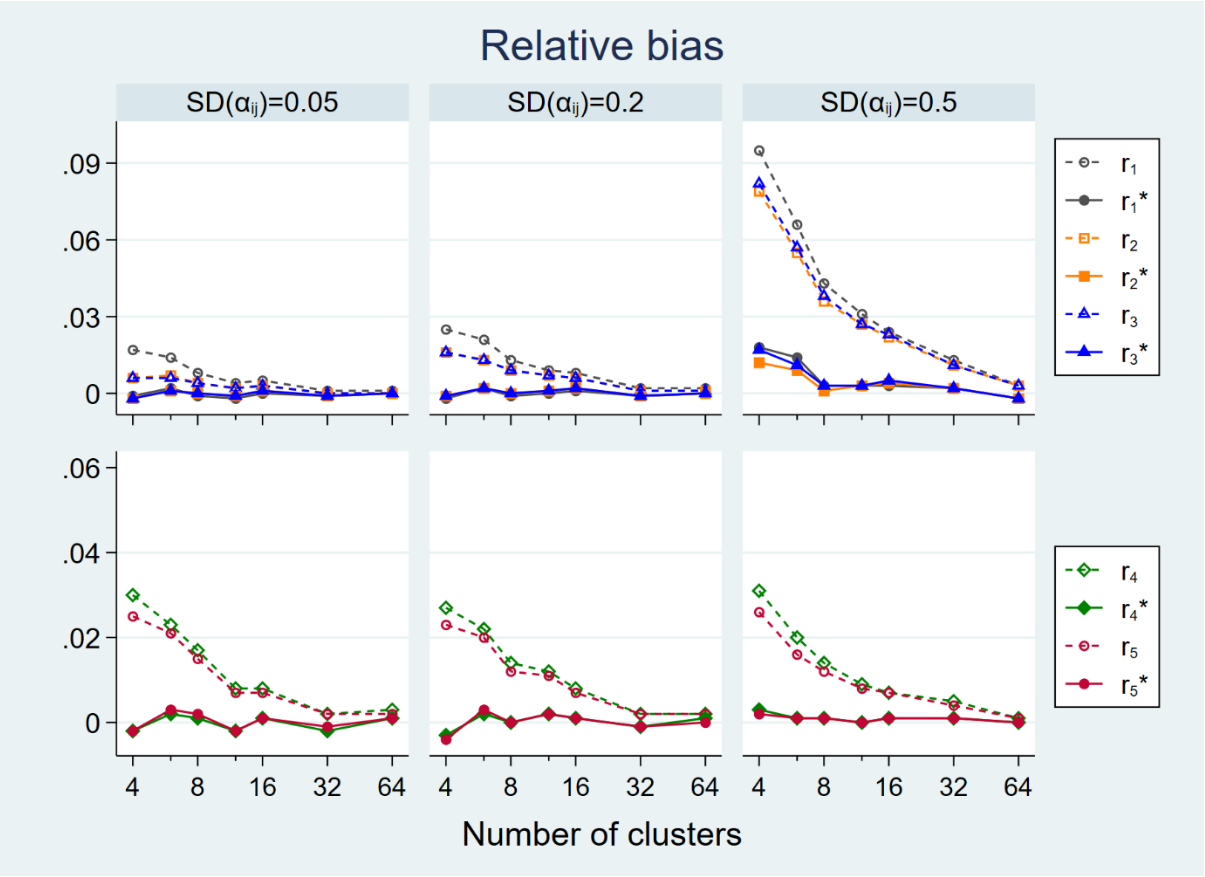

Figure S27. Relative bias of intervention effect estimators in relation to the number of clusters per trial arm for non-matched CRTs, by three levels of $SD(\alpha_{ij})$; population size per cluster follows a normal distribution with mean = 100 and $CV = 0.2$; intervention has a direct effect only $(\exp(\beta_D) = 0.5;\ \exp(\beta_I) = 1)$.

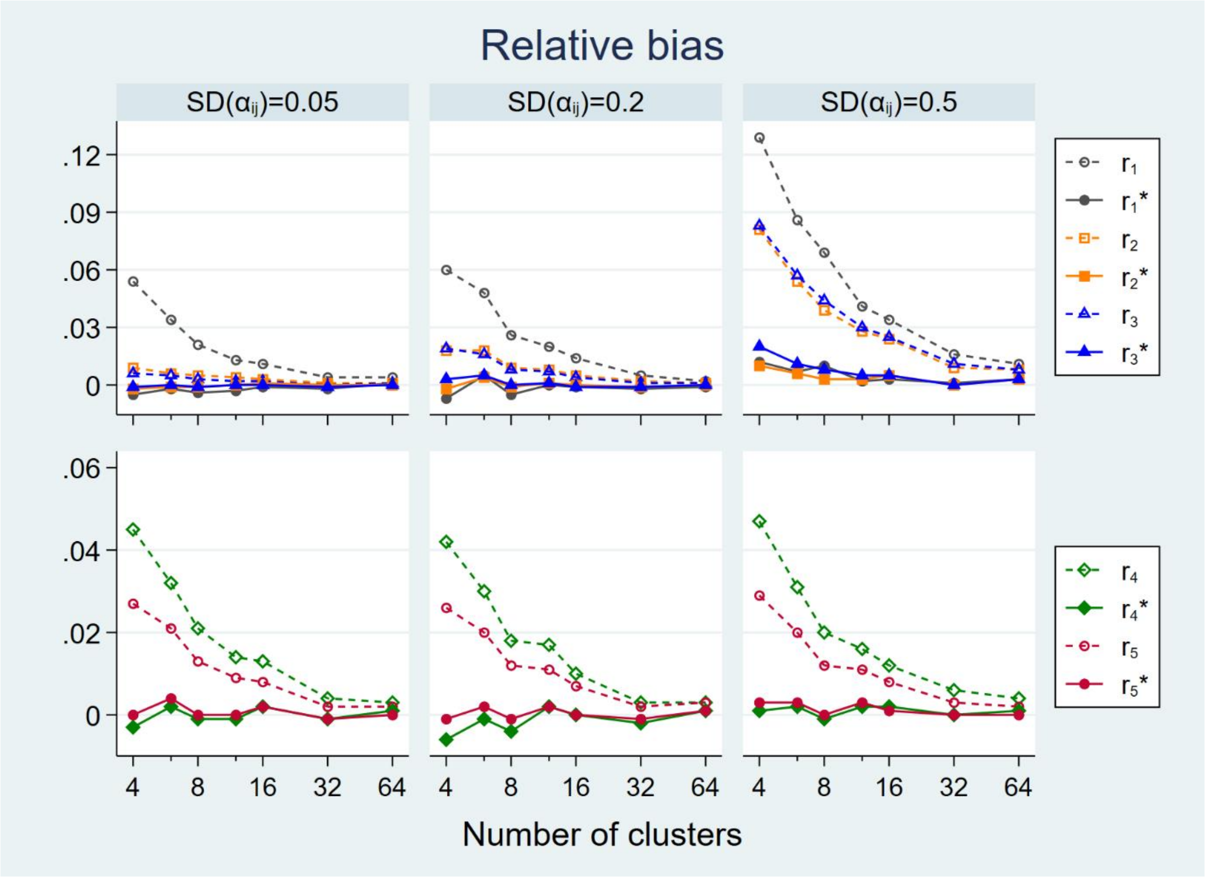

Figure S28. Relative bias of intervention effect estimators in relation to the number of clusters per trial arm for non-matched CRTs, by three levels of $SD(\alpha_{ij})$; population size per cluster follows a normal distribution with mean = 100 and $CV = 0.4$; intervention has a direct effect only $(\exp(\beta_D) = 0.5;\ \exp(\beta_I) = 1)$.

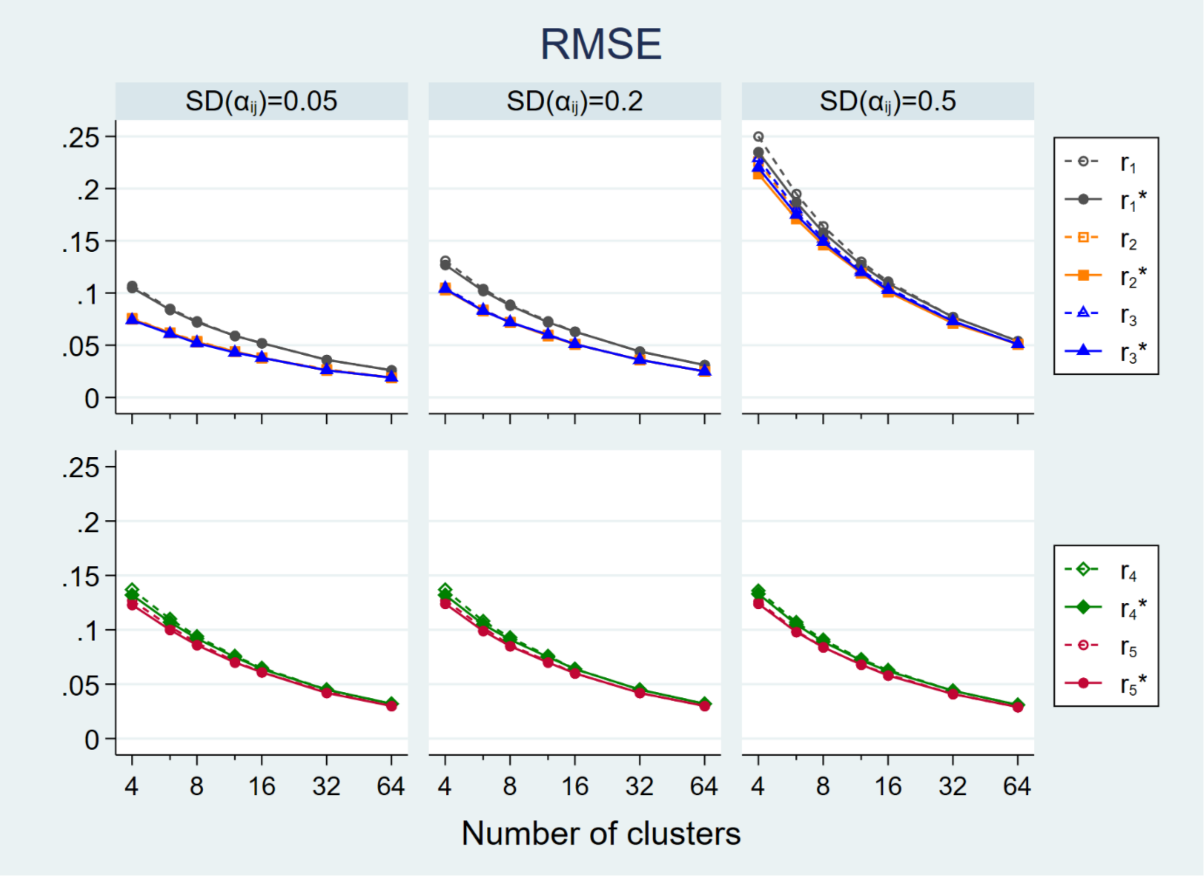

Figure S29. Root mean squared error (RMSE) of intervention effect estimators in relation to the number of clusters per trial arm for non-matched CRTs, by three levels of $SD(\alpha_{ij})$; population size per cluster follows a normal distribution with mean = 100 and $CV = 0.2$; intervention has a direct effect only $(\exp(\beta_D) = 0.5;\ \exp(\beta_I) = 1)$.

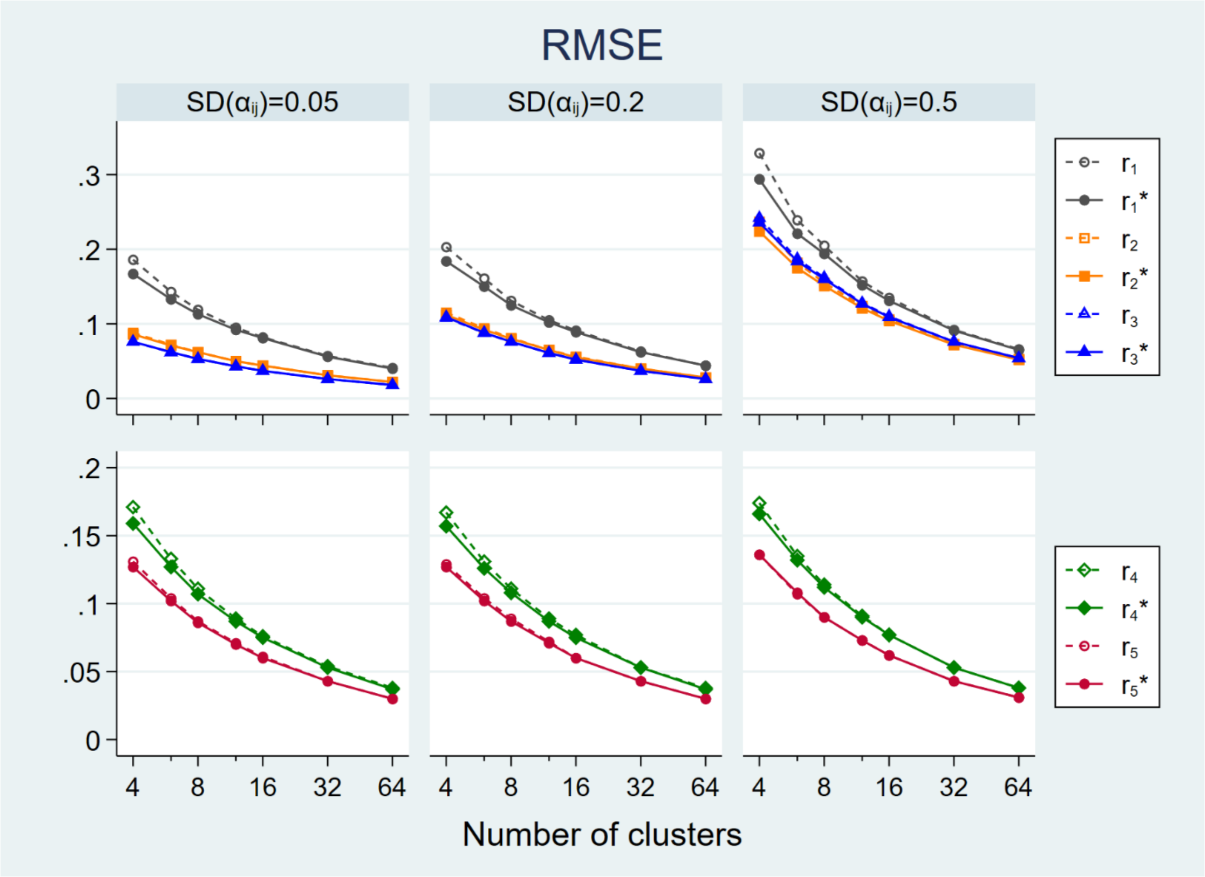

Figure S30. Root mean squared error (RMSE) of intervention effect estimators in relation to the number of clusters per trial arm for non-matched CRTs, by three levels of $SD(\alpha_{ij})$; population size per cluster follows a normal distribution with mean = 100 and $CV = 0.4$; intervention has a direct effect only $(\exp(\beta_D) = 0.5;\ \exp(\beta_I) = 1)$.

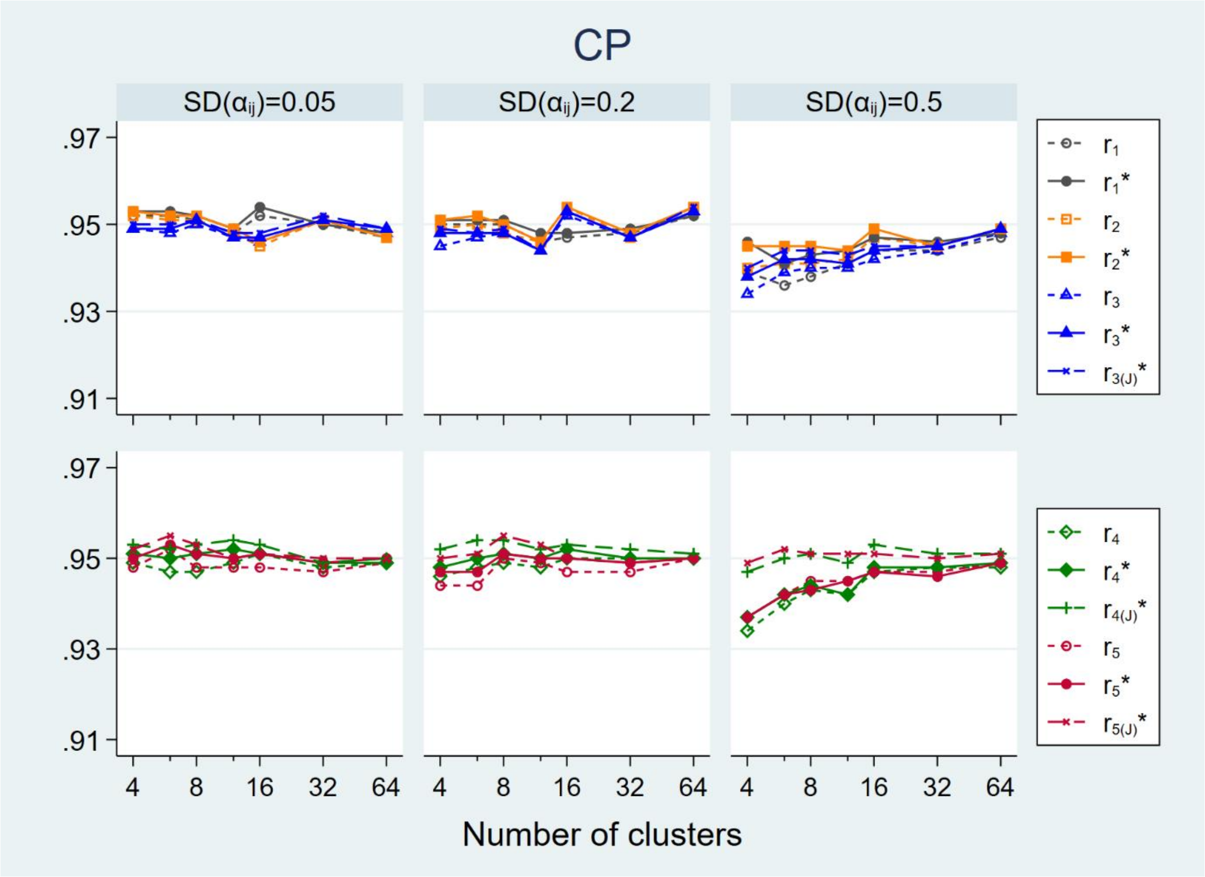

Figure S31 Coverage probability (CP) of 95% confidence interval (calculated on log-scale) of intervention effect estimators in relation to the number of clusters per trial arm for non-matched CRTs, by three levels of $SD(\alpha_{ij})$; population size per cluster follows a normal distribution with mean = 100 and $CV = 0.2$; intervention has a direct effect only $(\exp(\beta_D) = 0.5;\ \exp(\beta_I) = 1)$.

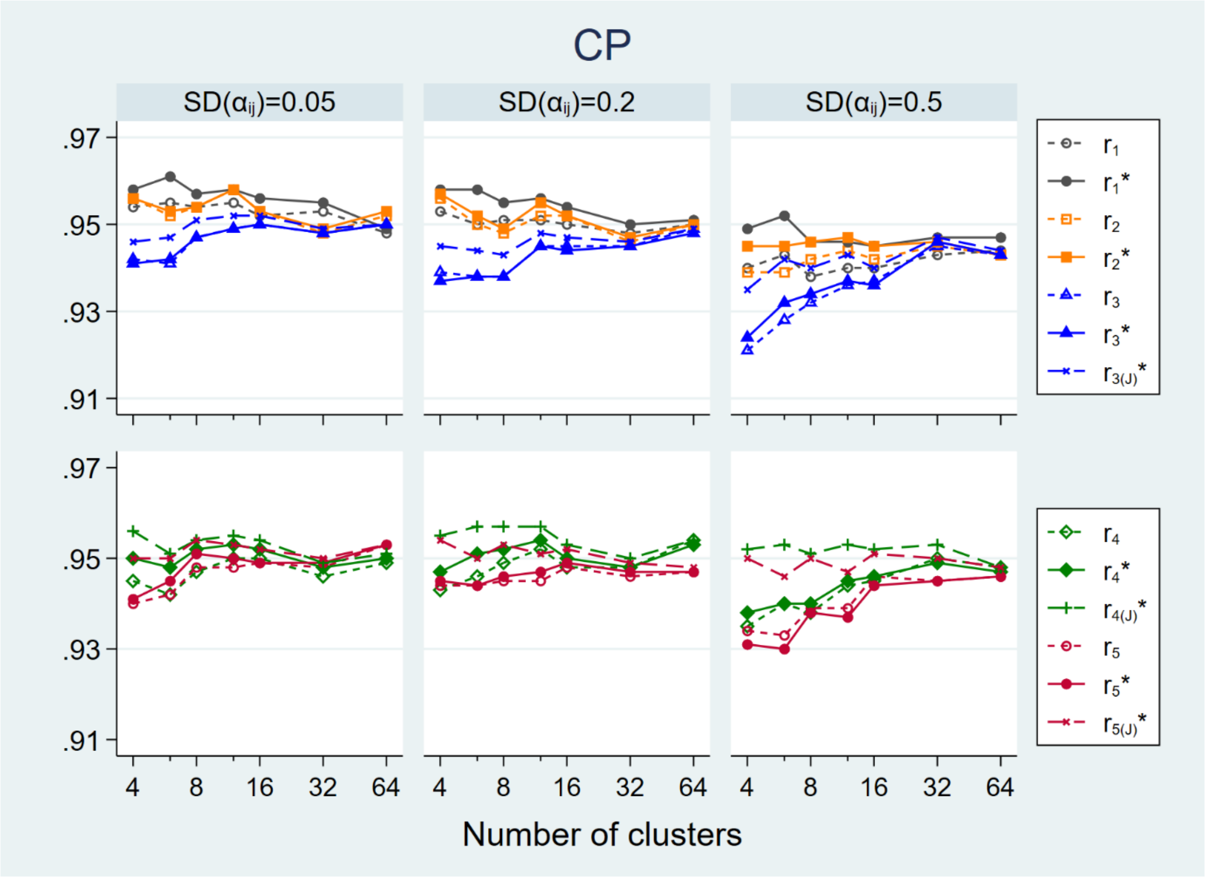

Figure S32. Coverage probability (CP) of 95% confidence interval (calculated on log-scale) of intervention effect estimators in relation to the number of clusters per trial arm for non-matched CRTs, by three levels of $SD(\alpha_{ij})$; population size per cluster follows a normal distribution with mean = 100 and $CV = 0.4$; intervention has a direct effect only $(\exp(\beta_D) = 0.5;\ \exp(\beta_I) = 1)$.

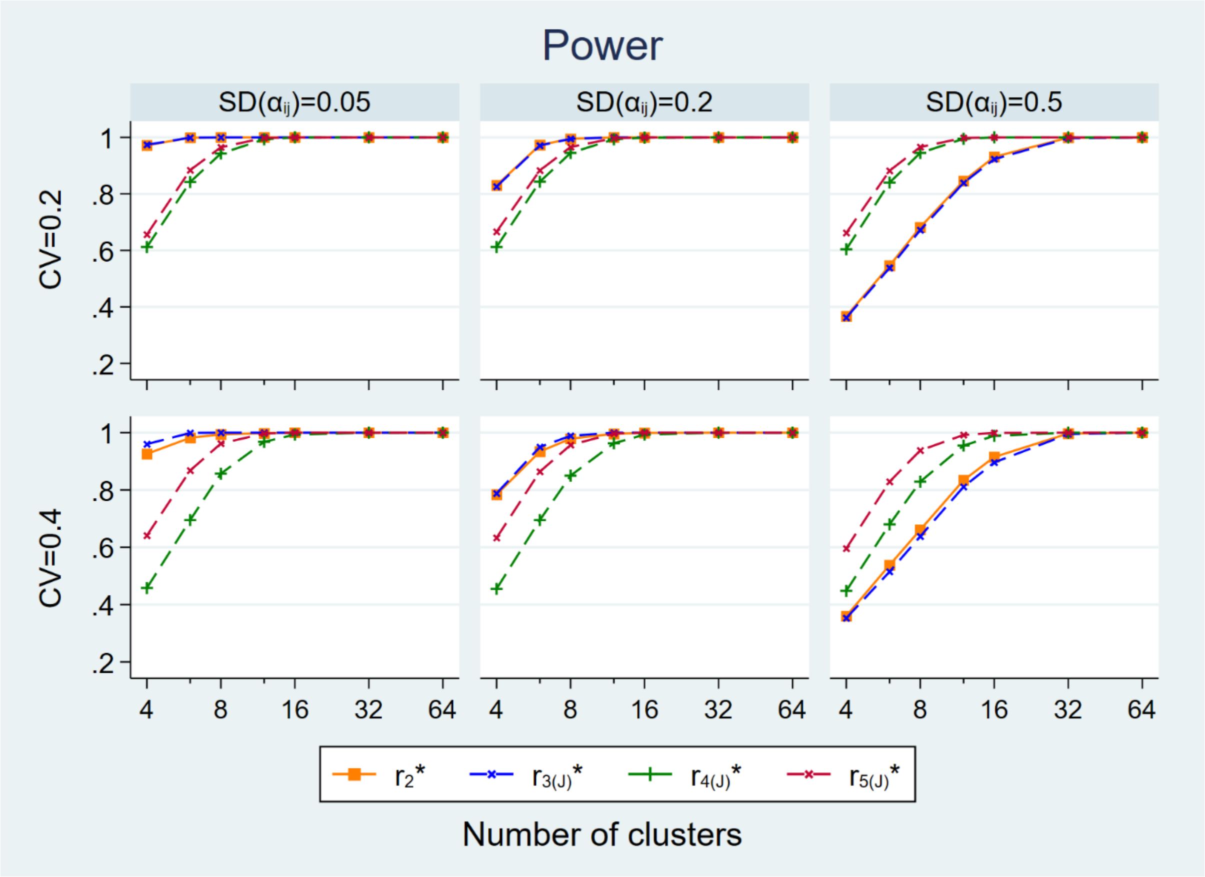

Figure S33. Power of $r_2^*, r_{3(J)}^*, r_{4(J)}^*$, and $r_{5(J)}^*$ in relation to the number of clusters per trial arm for non-matched CRTs, by three levels of $SD(\alpha_{ij})$; population size per cluster follows a normal distribution with mean = 100 and $CV = 0.2, 0.4$, respectively; intervention has a direct effect only $(\exp(\beta_D) = 0.5;\ \exp(\beta_I) = 1)$. Upper panel: $CV = 0.2$; lower panel: $CV = 0.4$.

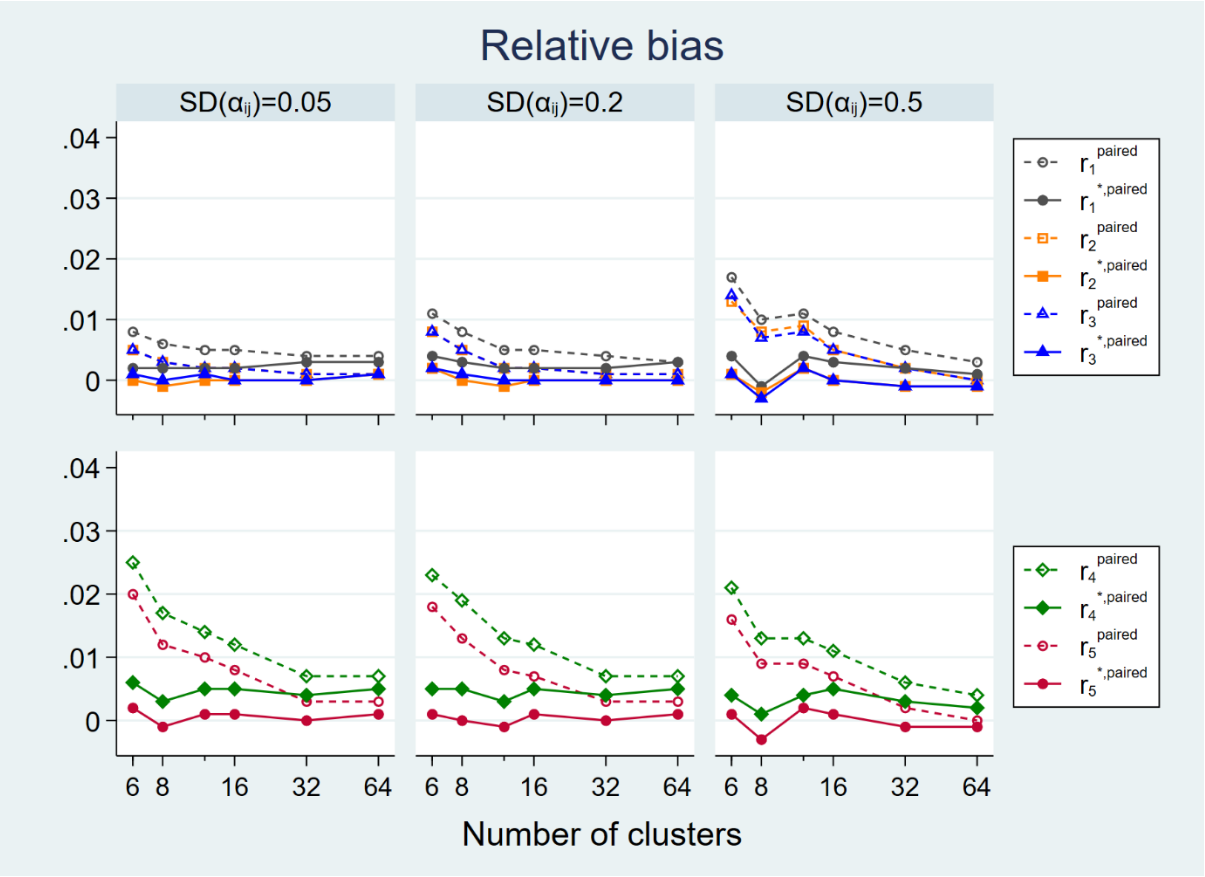

Figure S34. Relative bias of intervention effect estimators in relation to the number of clusters per trial arm for matched-pair CRTs, by three levels of $SD(\alpha_{ij})$; population size per cluster follows a skewed distribution with mean = 100 and $CV = 0.2$; intervention has a direct effect only $(\exp(\beta_D) = 0.5;\ \exp(\beta_I) = 1)$.

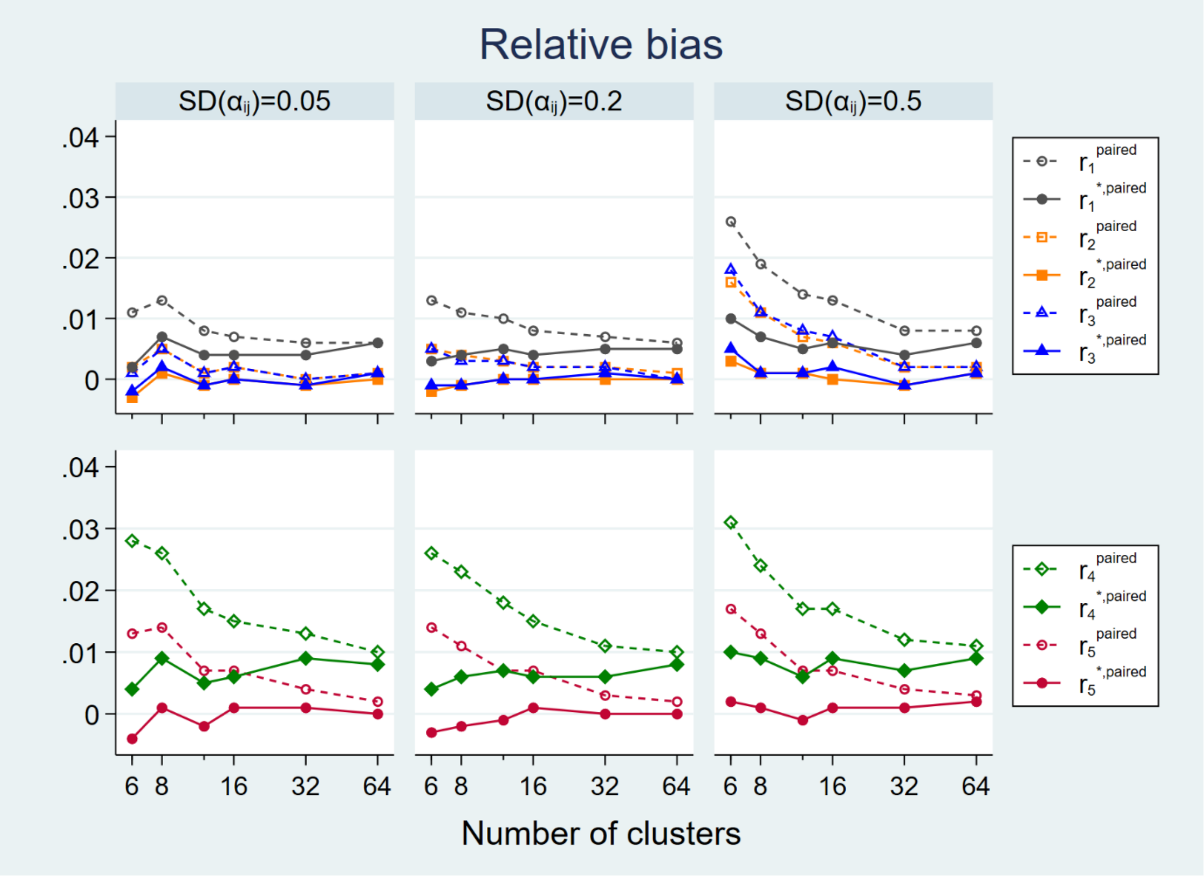

Figure S35. Relative bias of intervention effect estimators in relation to the number of clusters per trial arm for matched-pair CRTs, by three levels of $SD(\alpha_{ij})$; population size per cluster follows a skewed distribution with mean = 100 and $CV = 0.4$; intervention has a direct effect only $(\exp(\beta_D) = 0.5;\ \exp(\beta_I) = 1)$.

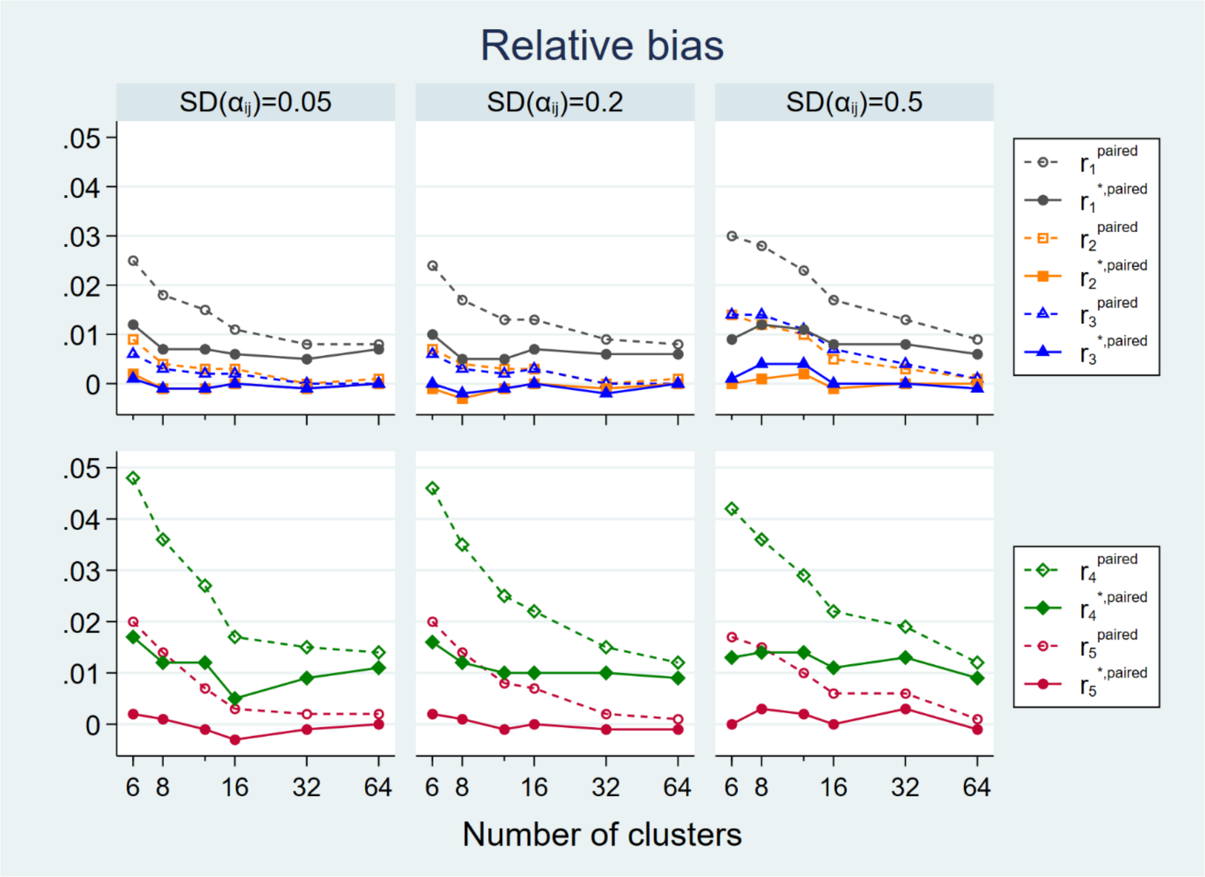

Figure S36. Relative bias of intervention effect estimators in relation to the number of clusters per trial arm for matched-pair CRTs, by three levels of $SD(\alpha_{ij})$; population size per cluster follows a skewed distribution with mean = 100 and $CV = 0.6$; intervention has a direct effect only $(\exp(\beta_D) = 0.5;\ \exp(\beta_I) = 1)$.

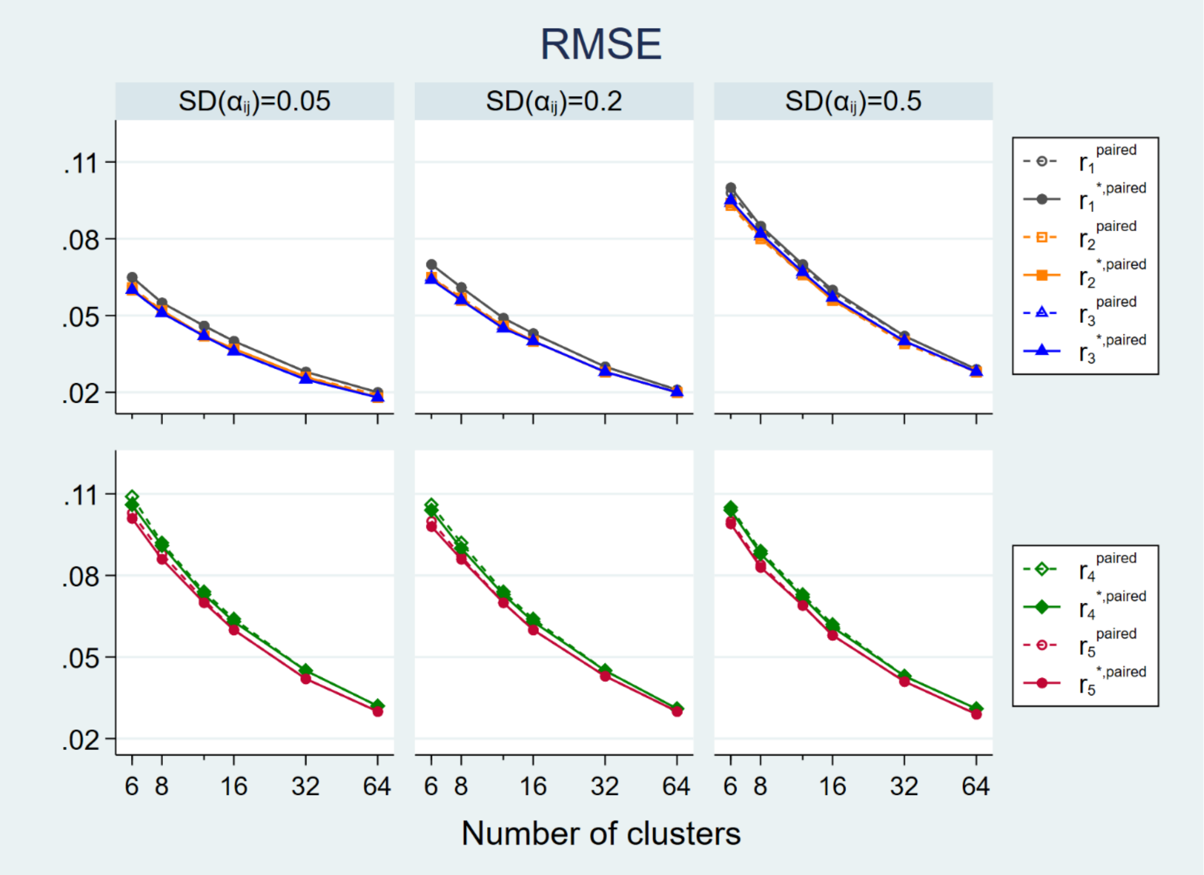

Figure S37. Root mean squared error (RMSE) of intervention effect estimators in relation to the number of clusters per trial arm for matched-pair CRTs, by three levels of $SD(\alpha_{ij})$; population size per cluster follows a skewed distribution with mean = 100 and $CV = 0.2$; intervention has a direct effect only ($\exp(\beta_D) = 0.5;\ \exp(\beta_I) = 1$).

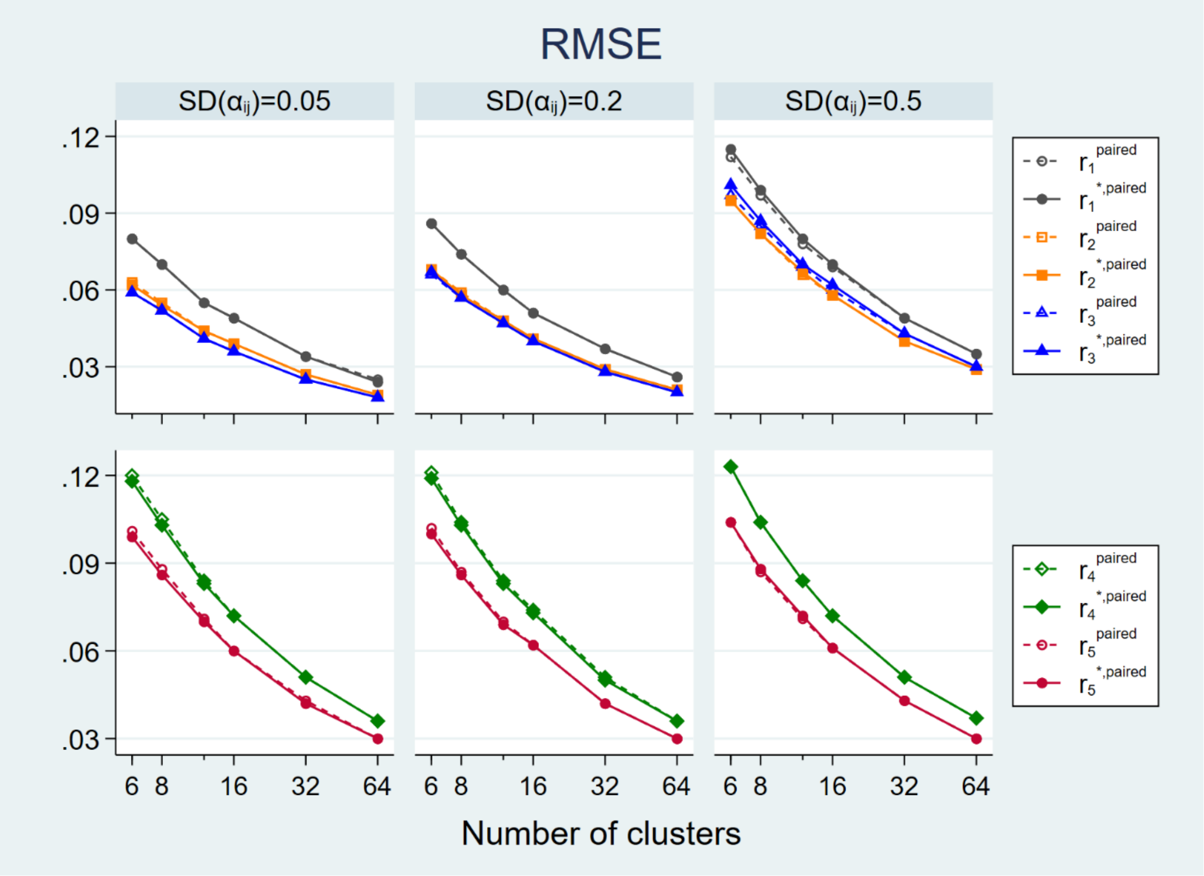

Figure S38. Root mean squared error (RMSE) of intervention effect estimators in relation to the number of clusters per trial arm for matched-pair CRTs, by three levels of $SD(\alpha_{ij})$; population size per cluster follows a skewed distribution with mean = 100 and $CV = 0.4$; intervention has a direct effect only $(\exp(\beta_D) = 0.5;\ \exp(\beta_I) = 1)$.

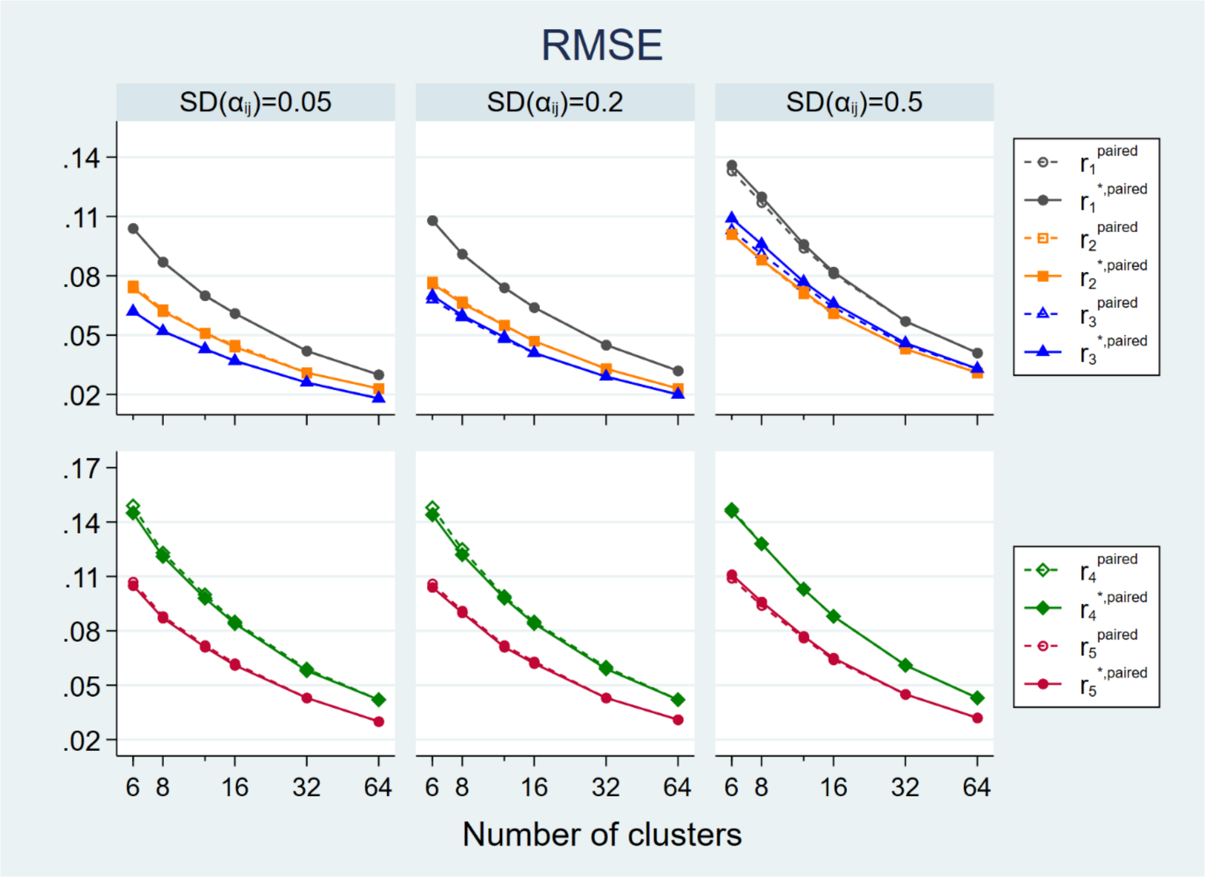

Figure S39. Root mean squared error (RMSE) of intervention effect estimators in relation to the number of clusters per trial arm for matched-pair CRTs, by three levels of $SD(\alpha_{ij})$; population size per cluster follows a skewed distribution with mean = 100 and $CV = 0.6$; intervention has a direct effect only $(\exp(\beta_D) = 0.5;\ \exp(\beta_I) = 1)$.

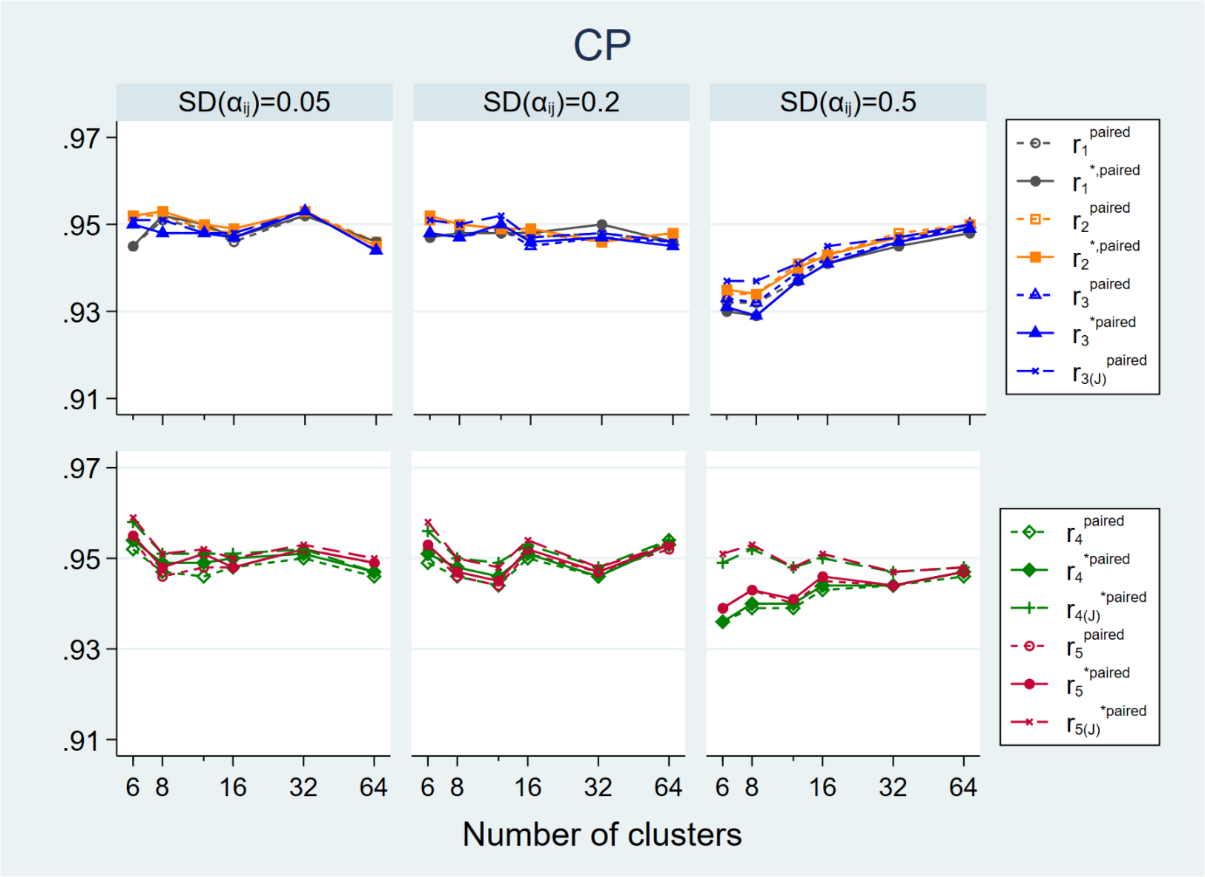

Figure S40. Coverage probability (CP) of 95% confidence interval (calculated on log-scale) of intervention effect estimators in relation to the number of clusters per trial arm for matched-pair CRTs, by three levels of $SD(\alpha_{ij})$; population size per cluster follows a skewed distribution with mean = 100 and $CV = 0.2$; intervention has a direct effect only $(\exp(\beta_D) = 0.5;\ \exp(\beta_I) = 1)$.

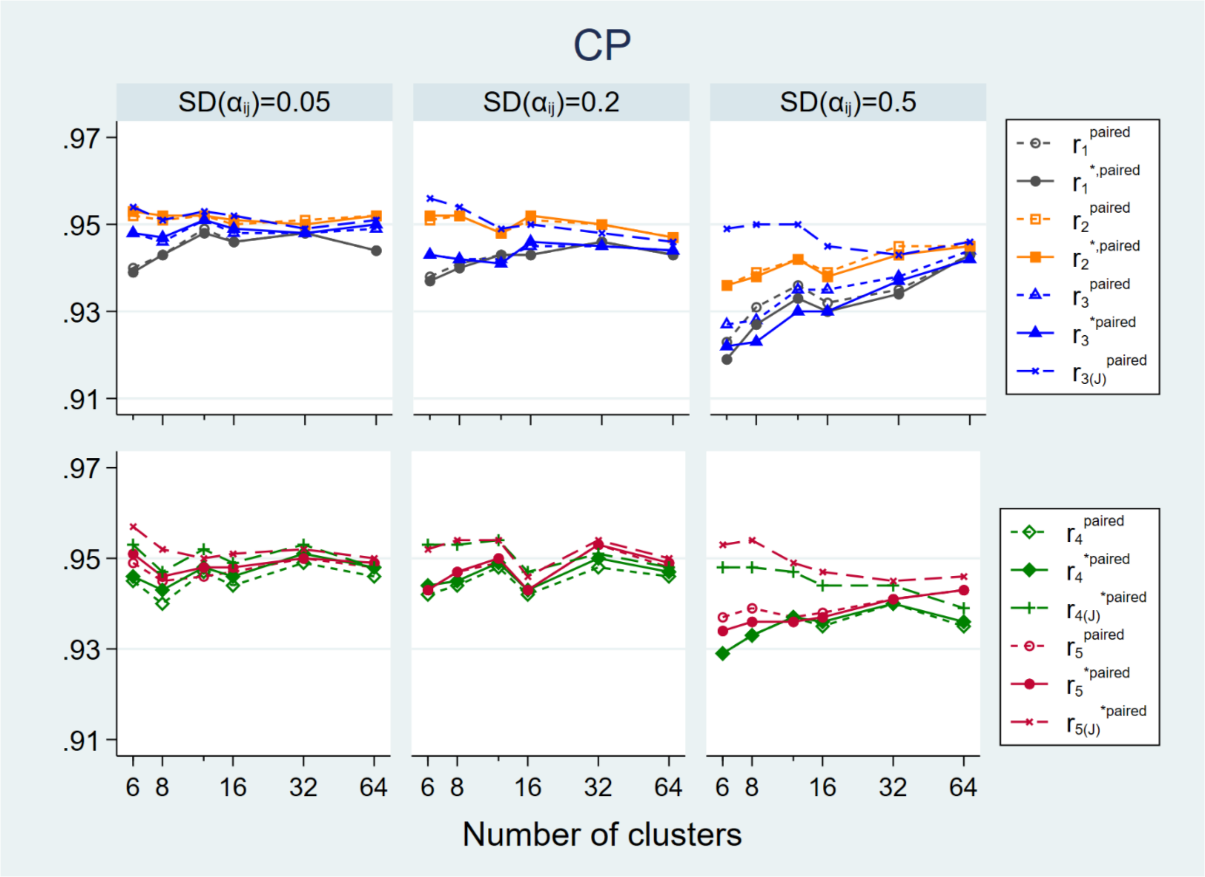

Figure S41. Coverage probability (CP) of 95% confidence interval (calculated on log-scale) of intervention effect estimators in relation to the number of clusters per trial arm for matched-pair CRTs, by three levels of $SD(\alpha_{ij})$; population size per cluster follows a skewed distribution with mean = 100 and $CV = 0.4$; intervention has a direct effect only $(\exp(\beta_D) = 0.5;\ \exp(\beta_I) = 1)$.

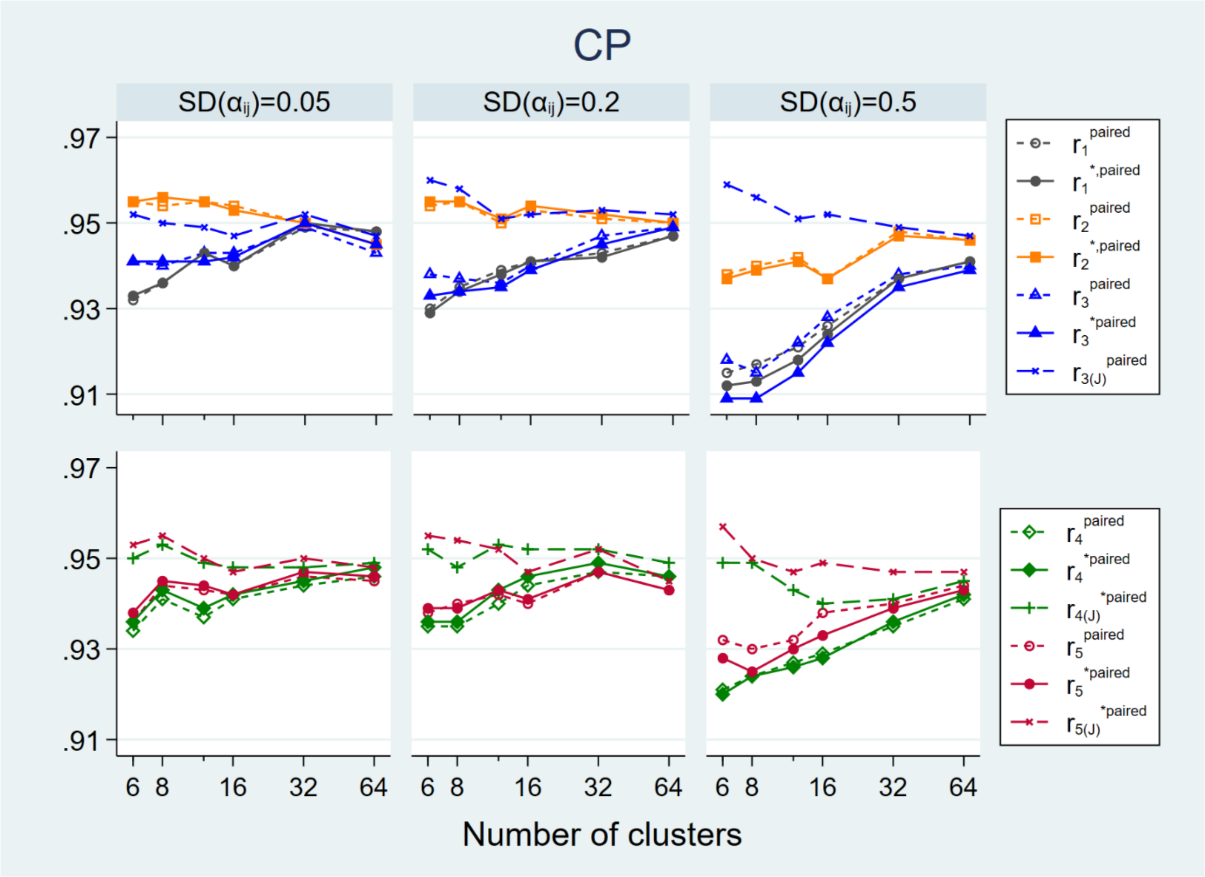

Figure S42. Coverage probability (CP) of 95% confidence interval (calculated on log-scale) of intervention effect estimators in relation to the number of clusters per trial arm for matched-pair CRTs, by three levels of $SD(\alpha_{ij})$; population size per cluster follows a skewed distribution with mean = 100 and $CV = 0.6$; intervention has a direct effect only $(\exp(\beta_D) = 0.5;\ \exp(\beta_I) = 1)$.

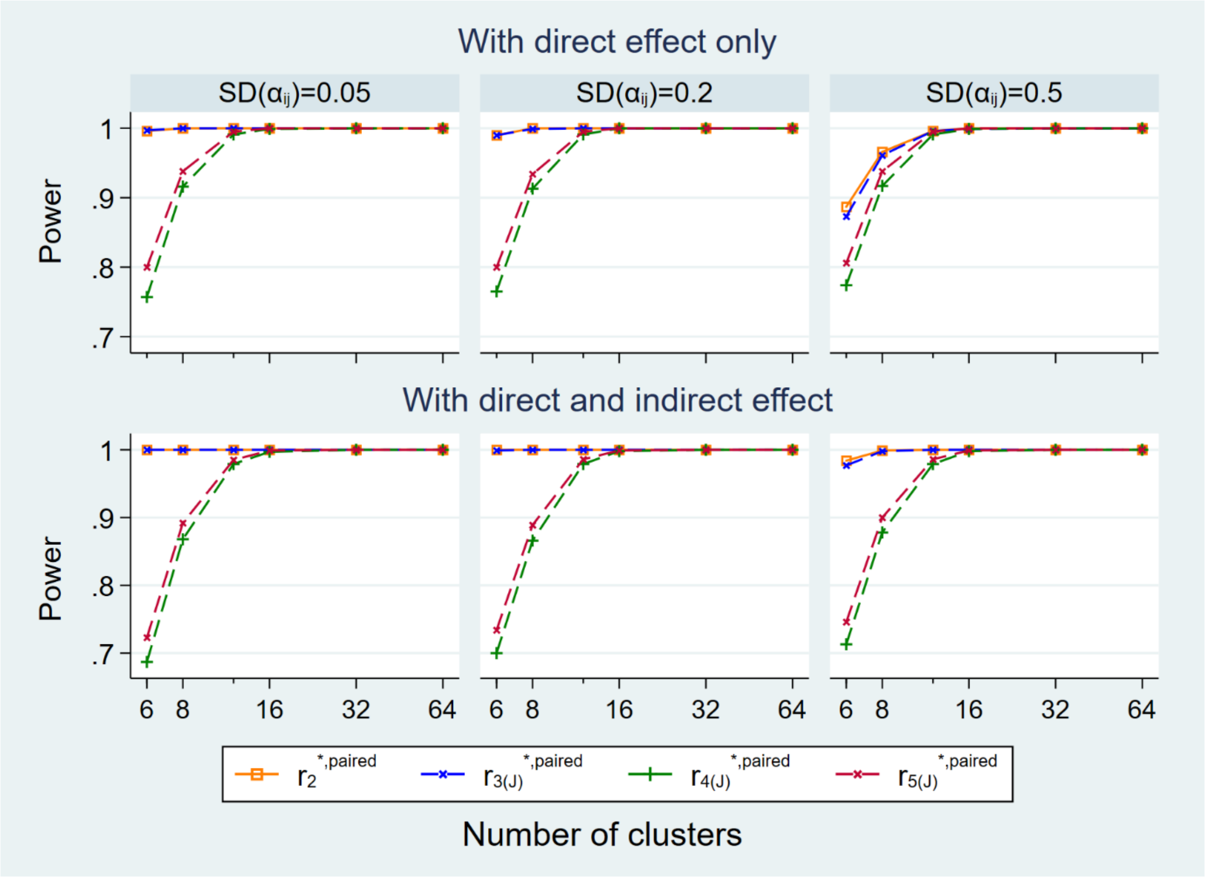

Figure S43. Power of $r_2^*, r_{3(J)}^*, r_{4(J)}^*$, and $r_{5(J)}^*$ in relation to the number of clusters per trial arm for matched-pair CRTs, by three levels of $SD(\alpha_{ij})$; population size per cluster follows a skewed distribution with mean = 100 and $CV = 0.2$. Upper panel: with direct effect only ($\exp(\beta_D) = 0.5;\ \exp(\beta_I) = 1$); lower panel: with direct and indirect effects ($\exp(\beta_D) = 0.5;\ \exp(\beta_I) = 0.75$).

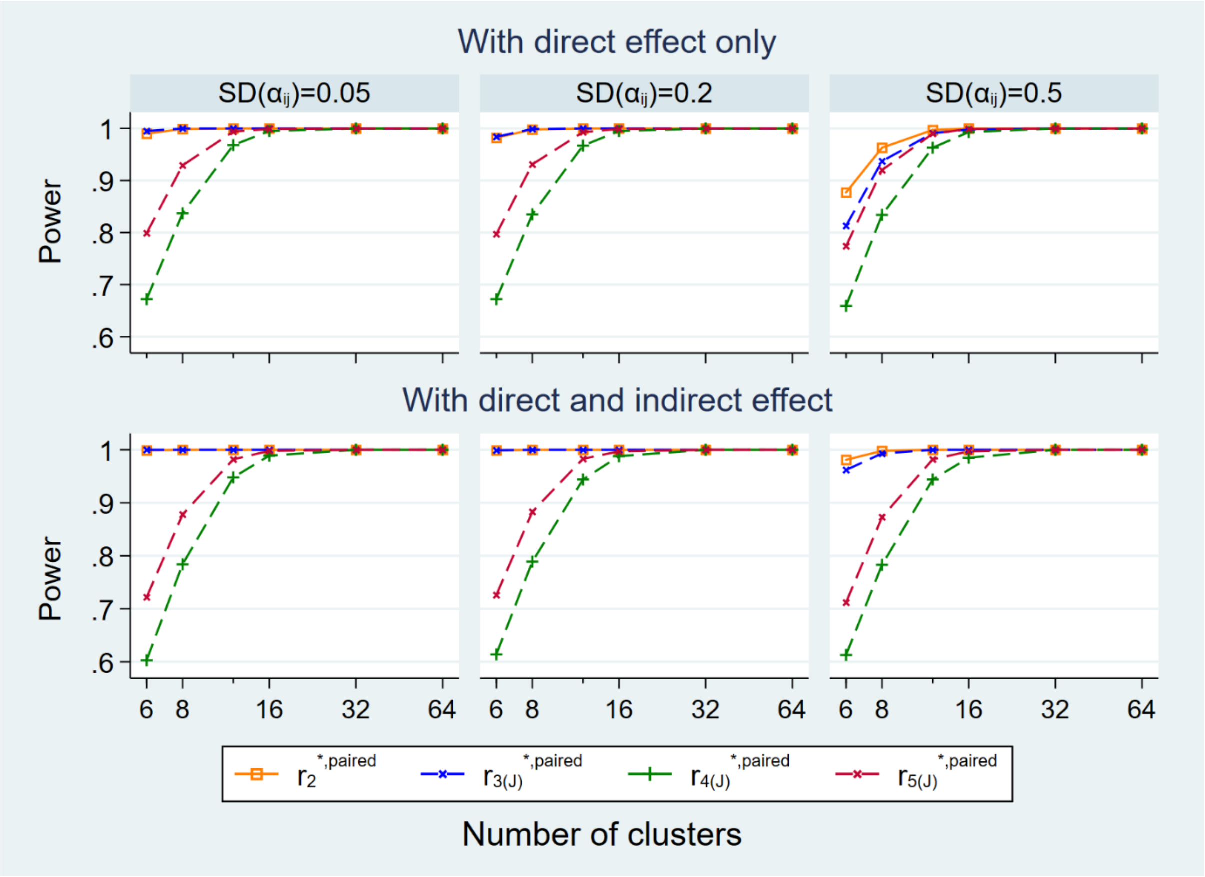

Figure S44. Power of $r_2^*, r_{3(J)}^*, r_{4(J)}^*$, and $r_{5(J)}^*$ in relation to the number of clusters per trial arm for matched-pair CRTs, by three levels of $SD(\alpha_{ij})$; population size per cluster follows a skewed distribution with mean = 100 and $CV = 0.4$. Upper panel: with direct effect only $(\exp(\beta_D) = 0.5;\ \exp(\beta_I) = 1)$; lower panel: with direct and indirect effects $(\exp(\beta_D) = 0.5;\ \exp(\beta_I) = 0.75)$.

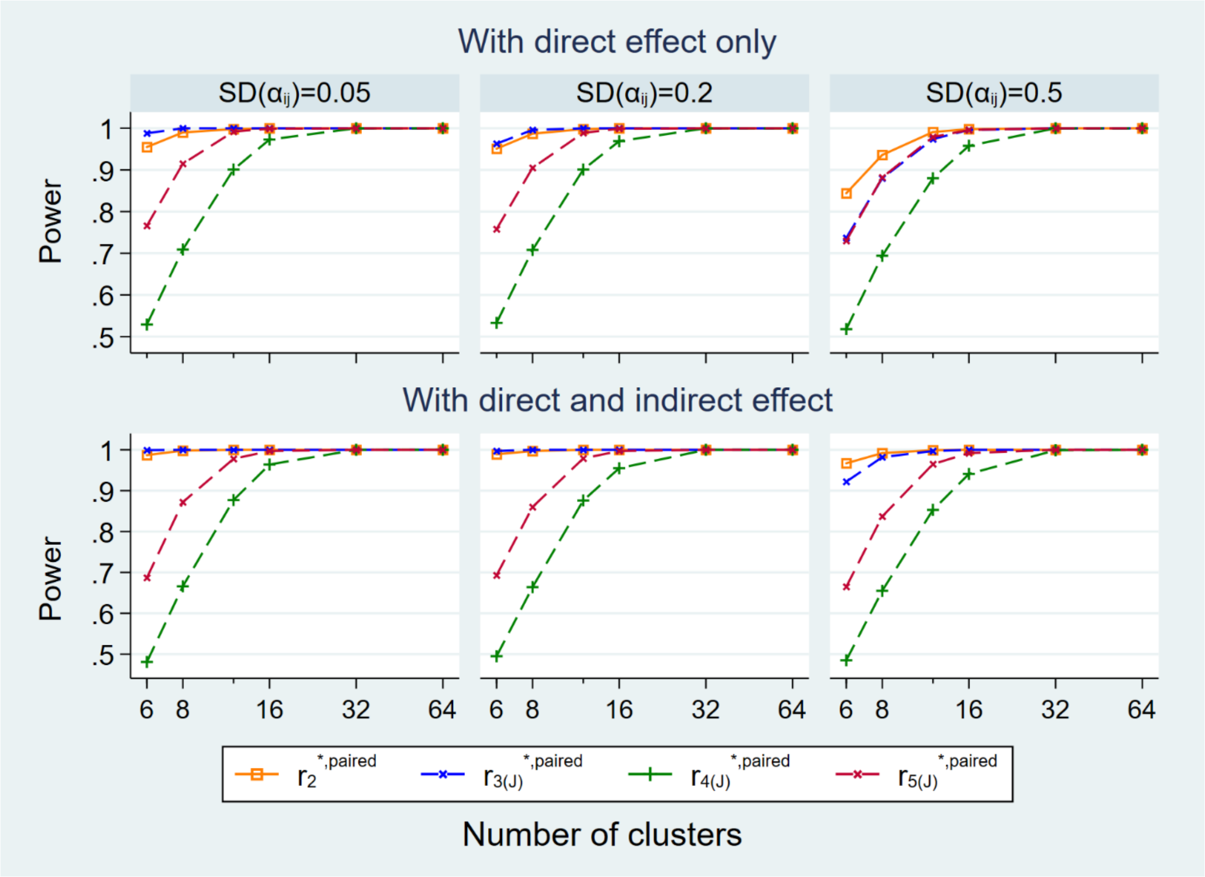

Figure S45. Power of $r_2^*, r_{3(J)}^*, r_{4(J)}^*$, and $r_{5(J)}^*$ in relation to the number of clusters per trial arm for matched-pair CRTs, by three levels of $SD(\alpha_{ij})$; population size per cluster follows a skewed distribution with mean = 100 and $CV = 0.6$. Upper panel: with direct effect only ($\exp(\beta_D) = 0.5;\ \exp(\beta_I) = 1$); lower panel: with direct and indirect effects ($\exp(\beta_D) = 0.5;\ \exp(\beta_I) = 0.75$).

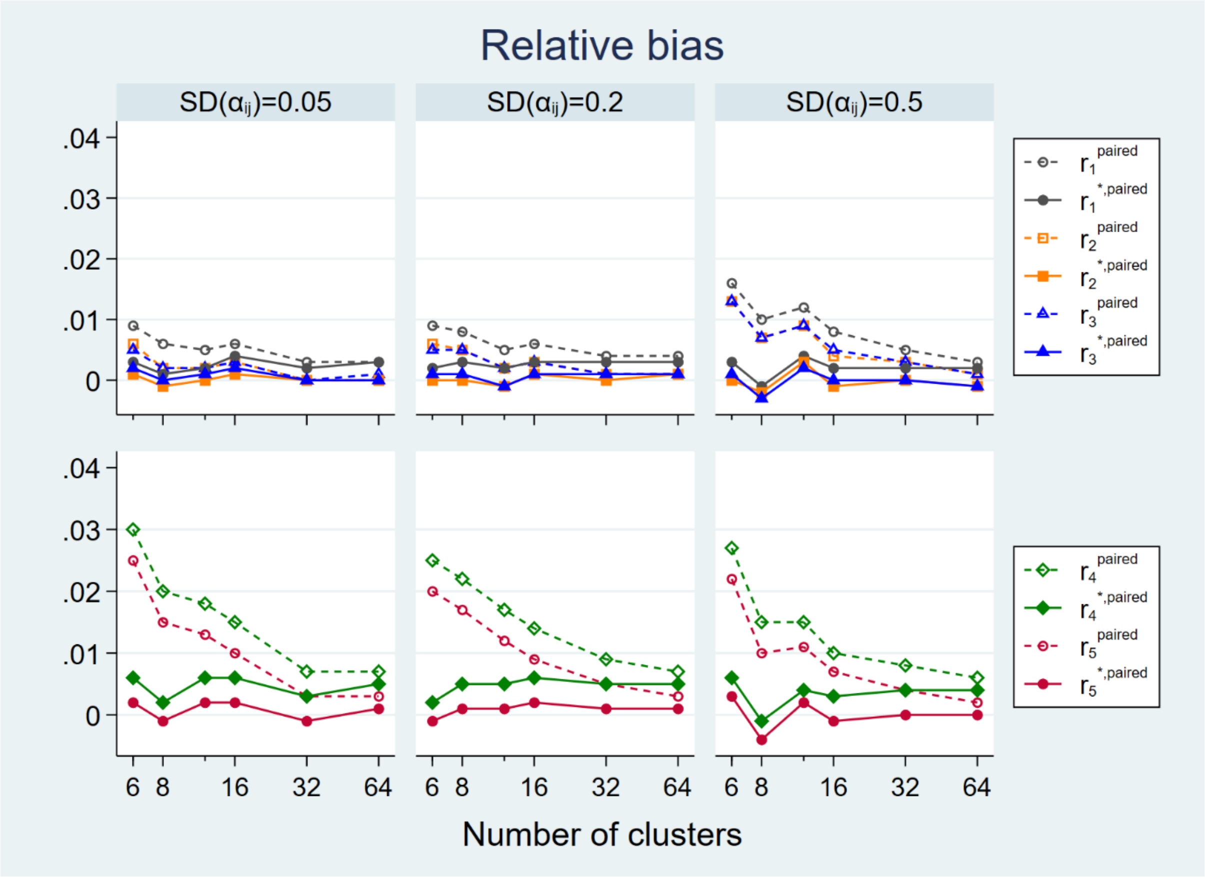

Figure S46. Relative bias of intervention effect estimators in relation to the number of clusters per trial arm for matched-pair CRTs, by three levels of $SD(\alpha_{ij})$; population size per cluster follows a skewed distribution with mean = 100 and $CV = 0.2$; intervention has direct and indirect effects ($\exp(\beta_D) = 0.5;\ \exp(\beta_I) = 0.75$).

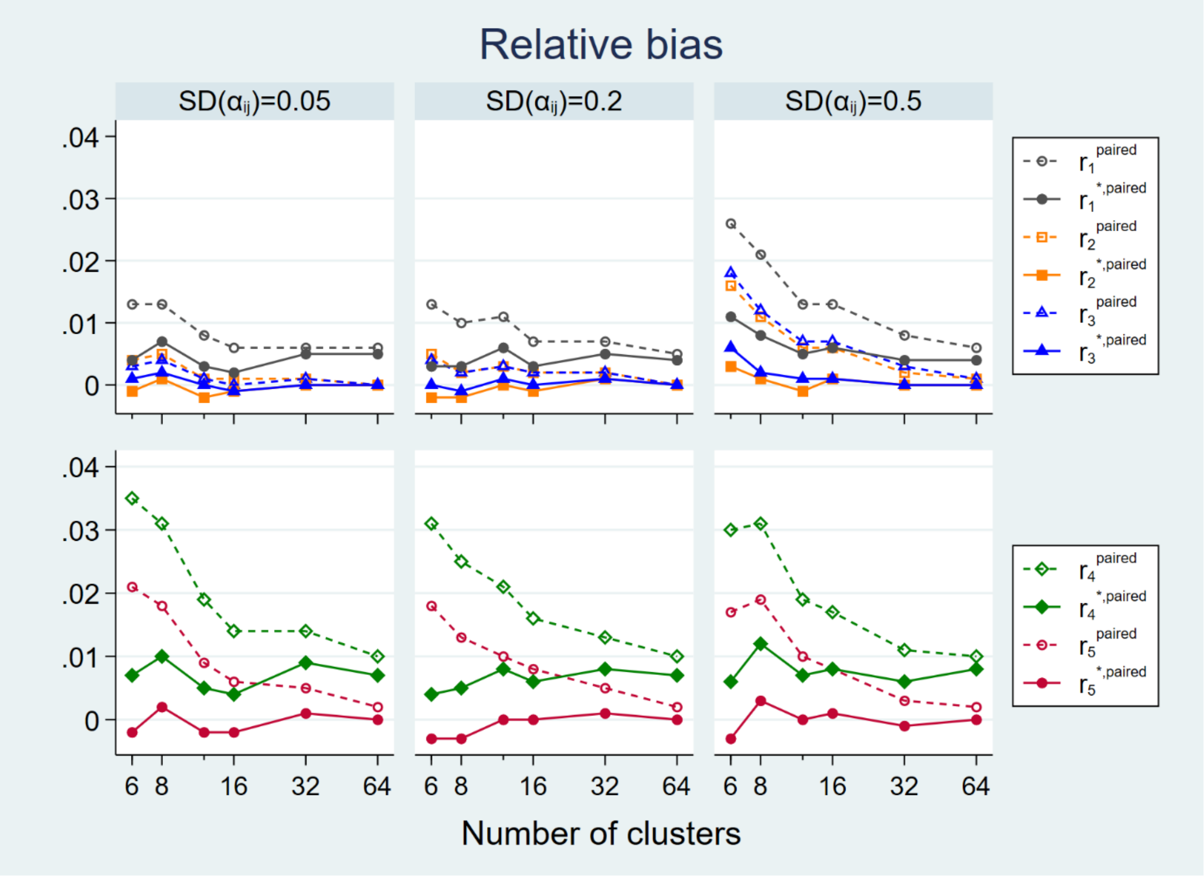

Figure S47. Relative bias of intervention effect estimators in relation to the number of clusters per trial arm for matched-pair CRTs, by three levels of $SD(\alpha_{ij})$; population size per cluster follows a skewed distribution with mean = 100 and $CV = 0.4$; intervention has direct and indirect effects ($\exp(\beta_D) = 0.5;\ \exp(\beta_I) = 0.75$).

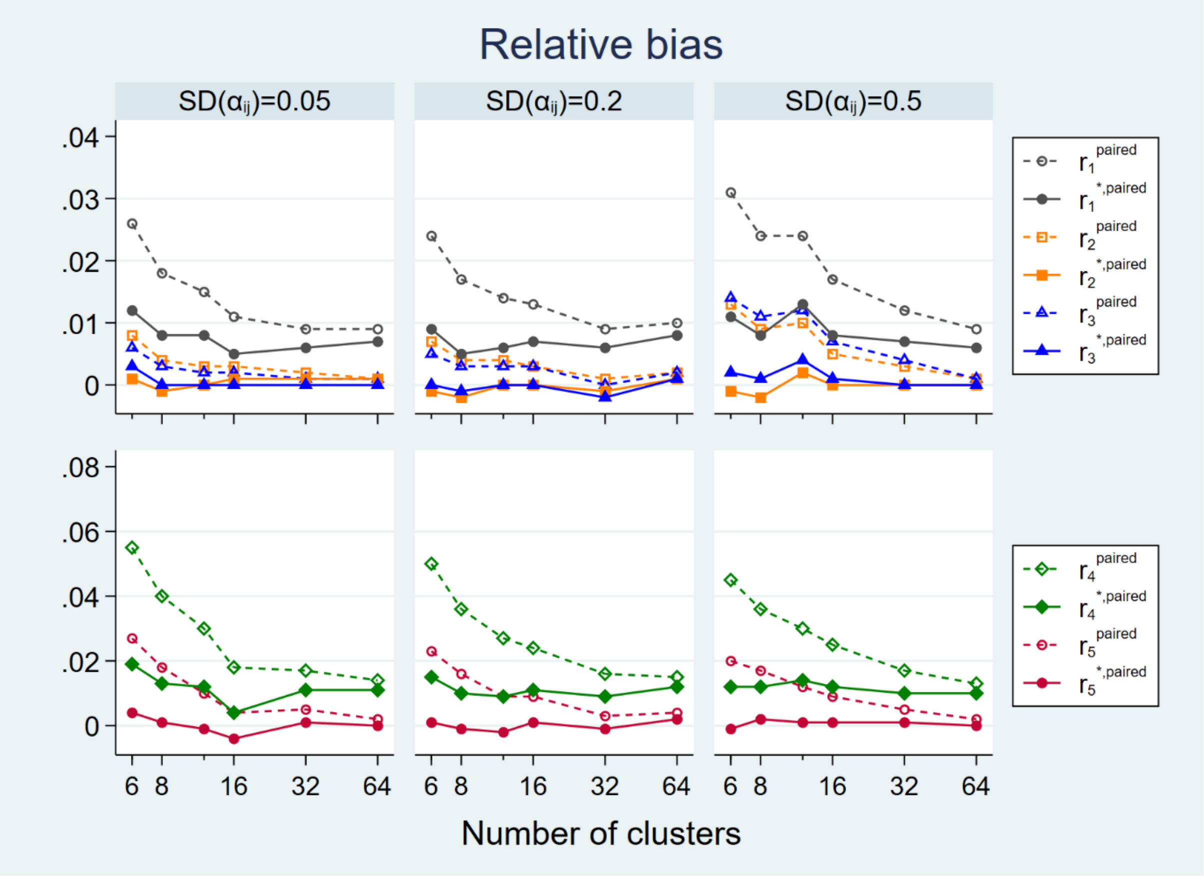

Figure S48. Relative bias of intervention effect estimators in relation to the number of clusters per trial arm for matched-pair CRTs, by three levels of $SD(\alpha_{ij})$; population size per cluster follows a skewed distribution with mean = 100 and $CV = 0.6$; intervention has direct and indirect effects $(\exp(\beta_D) = 0.5;\ \exp(\beta_I) = 0.75)$.

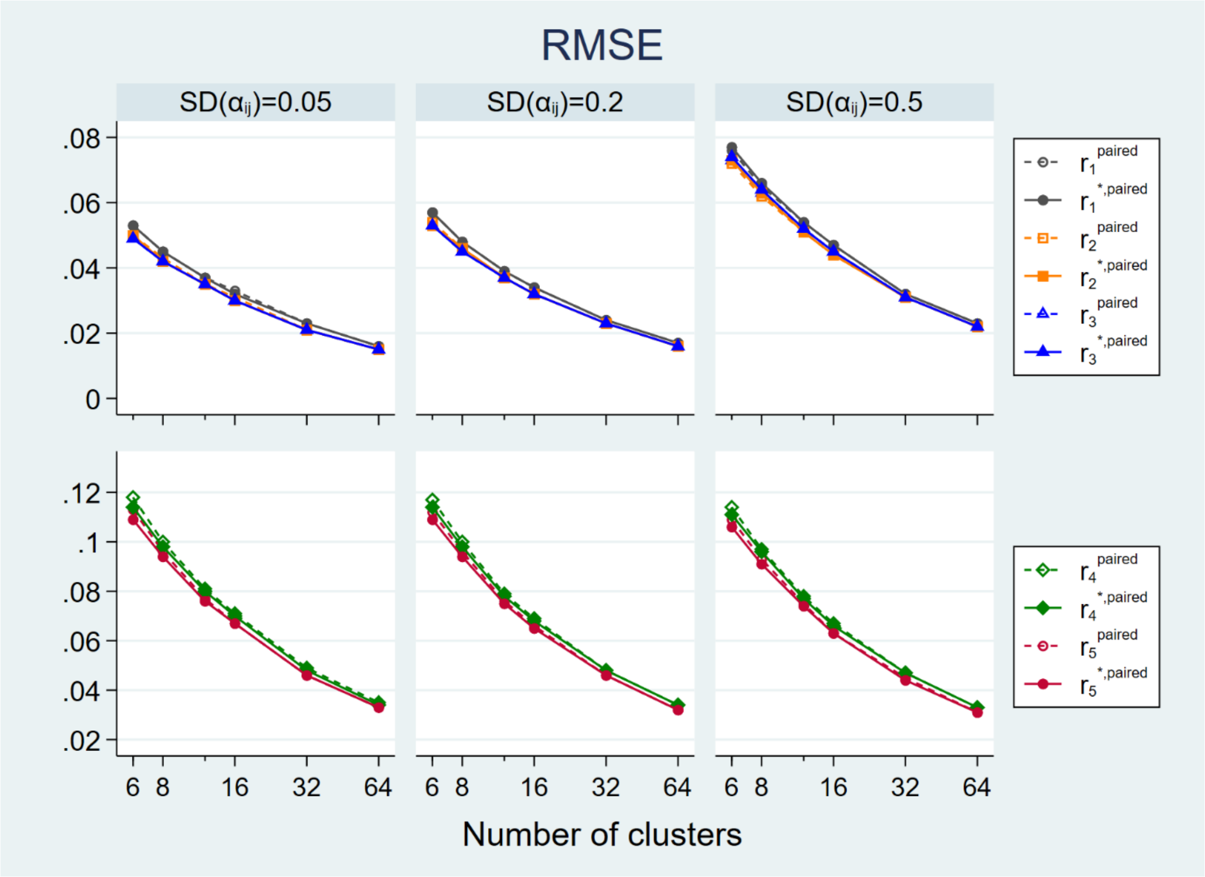

Figure S49. Root mean squared error (RMSE) of intervention effect estimators in relation to the number of clusters per trial arm for matched-pair CRTs, by three levels of $SD(\alpha_{ij})$; population size per cluster follows a skewed distribution with mean = 100 and $CV = 0.2$; intervention has direct and indirect effects $(\exp(\beta_D) = 0.5;\ \exp(\beta_I) = 0.75)$.

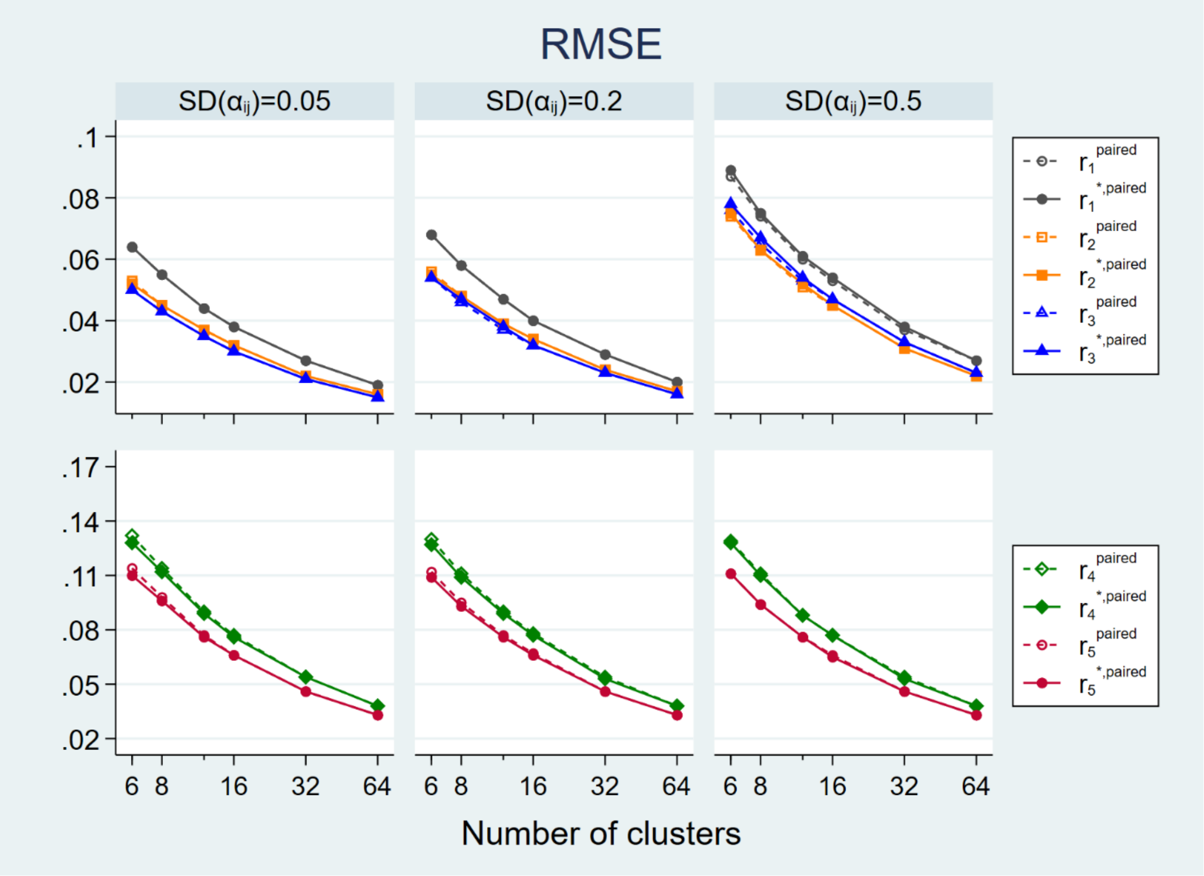

Figure S50. Root mean squared error (RMSE) of intervention effect estimators in relation to the number of clusters per trial arm for matched-pair CRTs, by three levels of $SD(\alpha_{ij})$; population size per cluster follows a skewed distribution with mean = 100 and $CV = 0.4$; intervention has direct and indirect effects $(\exp(\beta_D) = 0.5;\ \exp(\beta_I) = 0.75)$.

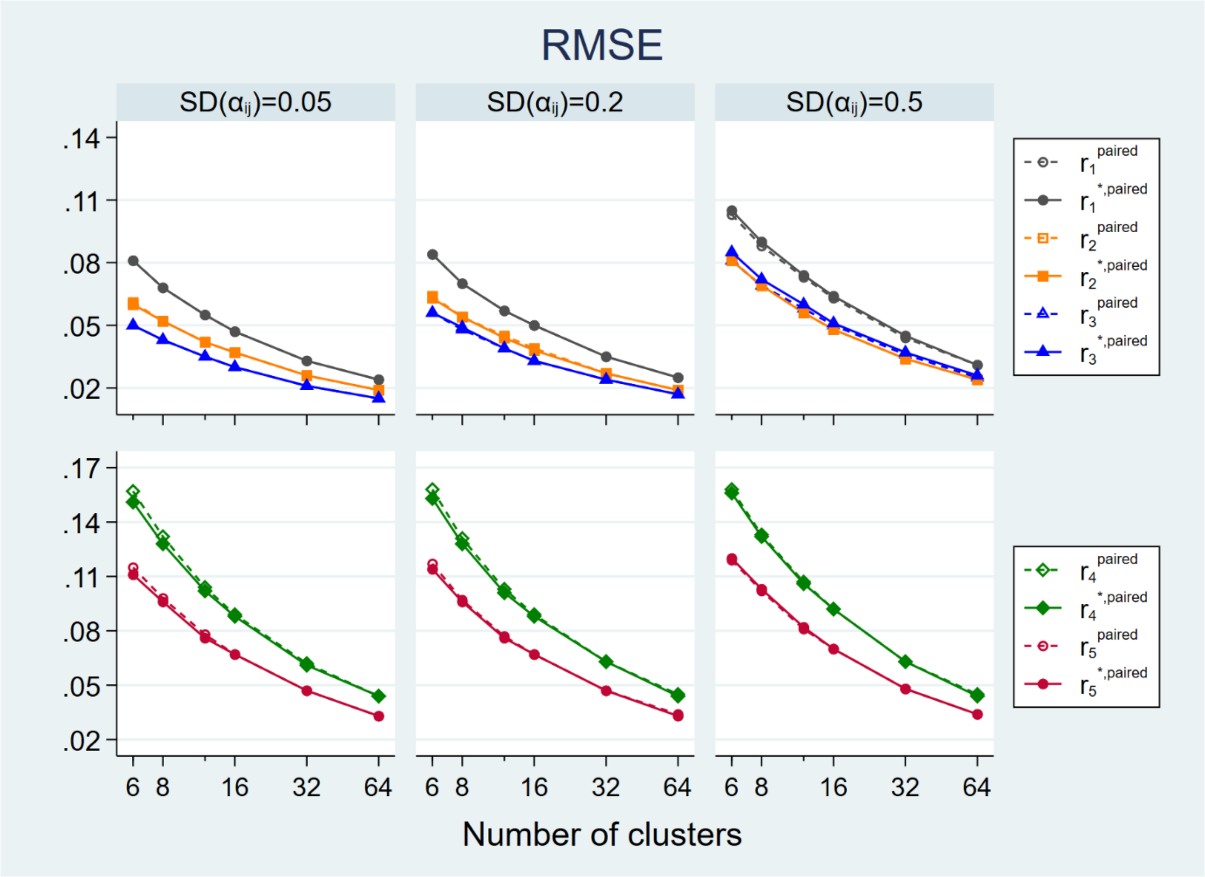

Figure S51. Root mean squared error (RMSE) of intervention effect estimators in relation to the number of clusters per trial arm for matched-pair CRTs, by three levels of $SD(\alpha_{ij})$; population size per cluster follows a skewed distribution with mean = 100 and $CV = 0.6$; intervention has direct and indirect effects $(\exp(\beta_D) = 0.5;\ \exp(\beta_I) = 0.75)$.

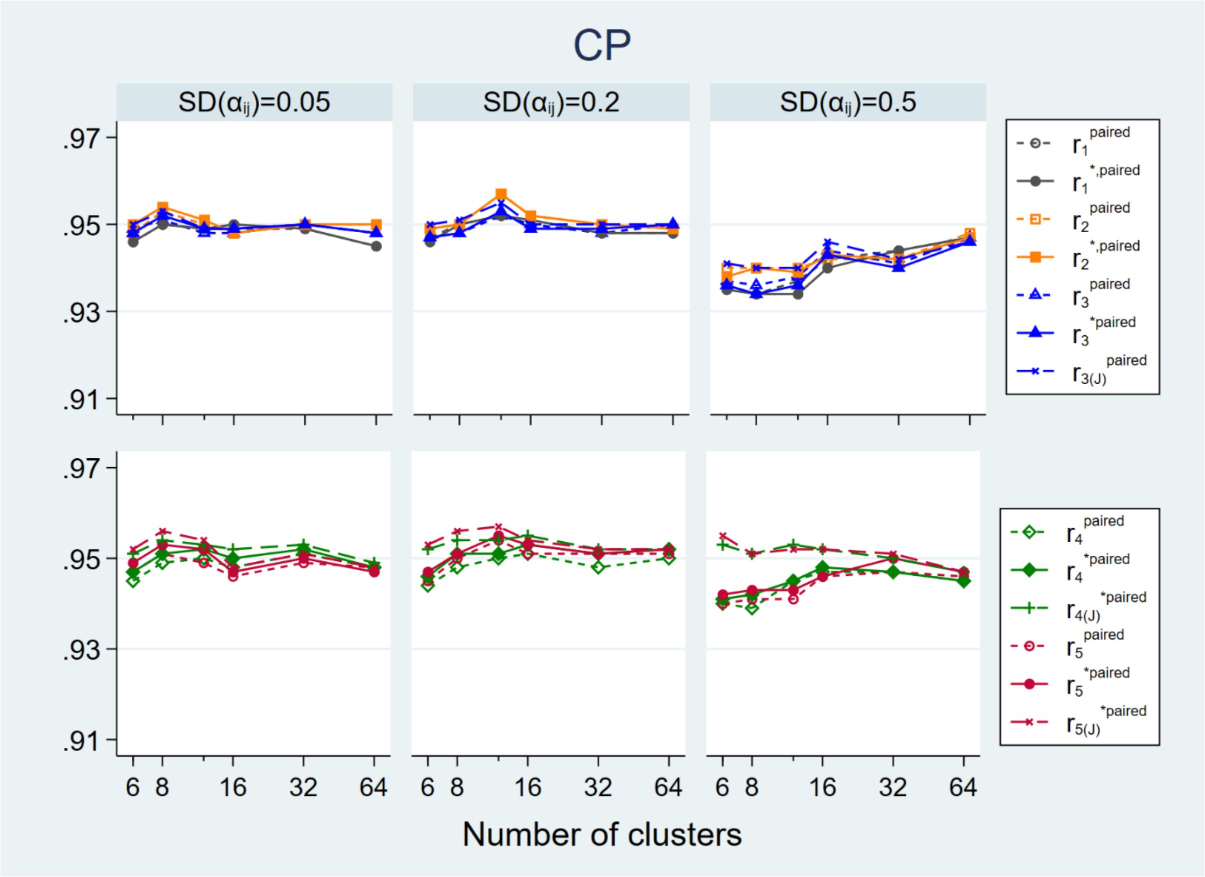

Figure S52. Coverage probability (CP) of 95% confidence interval (calculated on log-scale) of intervention effect estimators in relation to the number of clusters per trial arm for matched-pair CRTs, by three levels of $SD(\alpha_{ij})$; population size per cluster follows a skewed distribution with mean = 100 and $CV(p_{ij}) = 0.2$; intervention has direct and indirect effects ($\exp(\beta_D) = 0.5;\ \exp(\beta_I) = 0.75$).

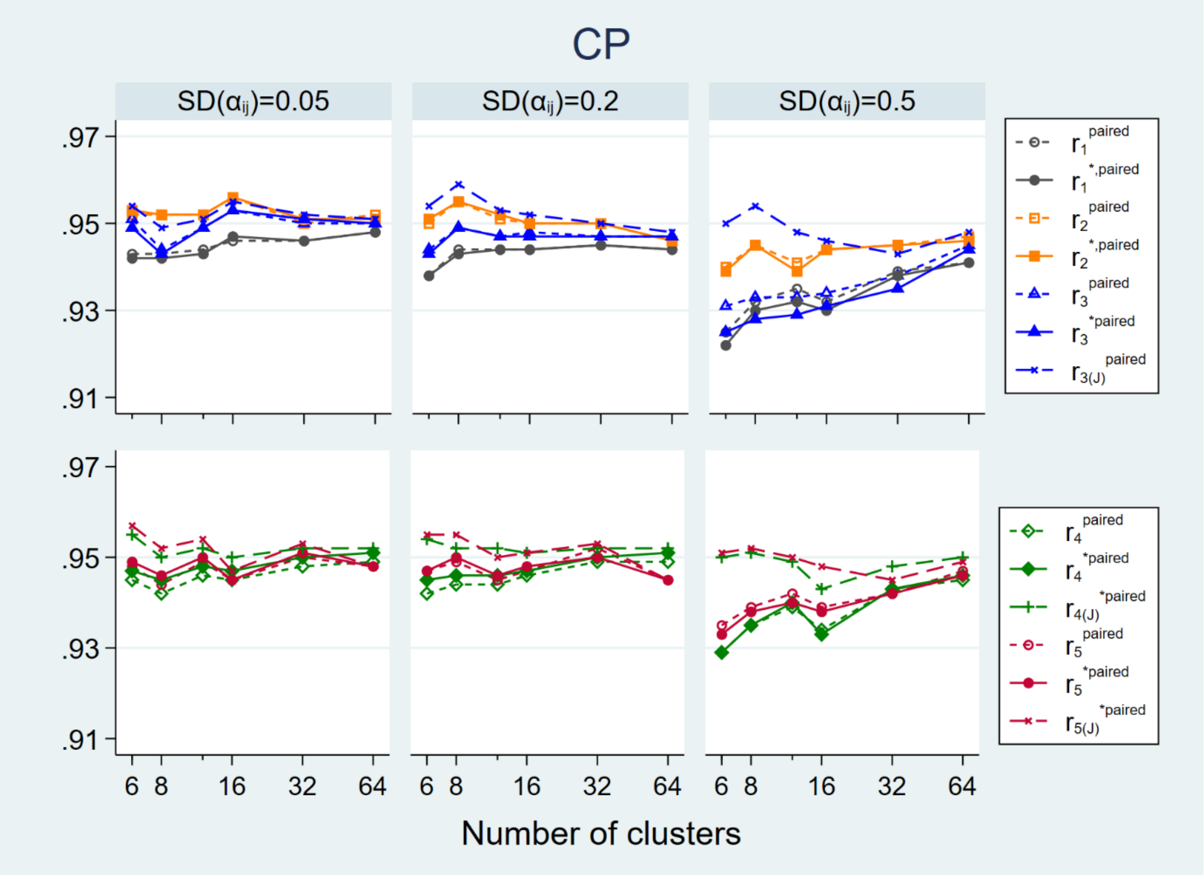

Figure S53. Coverage probability (CP) of 95% confidence interval (calculated on log-scale) of intervention effect estimators in relation to the number of clusters per trial arm for matched-pair CRTs, by three levels of $SD(\alpha_{ij})$; population size per cluster follows a skewed distribution with mean = 100 and d $CV = 0.4$; intervention has direct and indirect effects $(\exp(\beta_D) = 0.5;\ \exp(\beta_I) = 0.75)$.

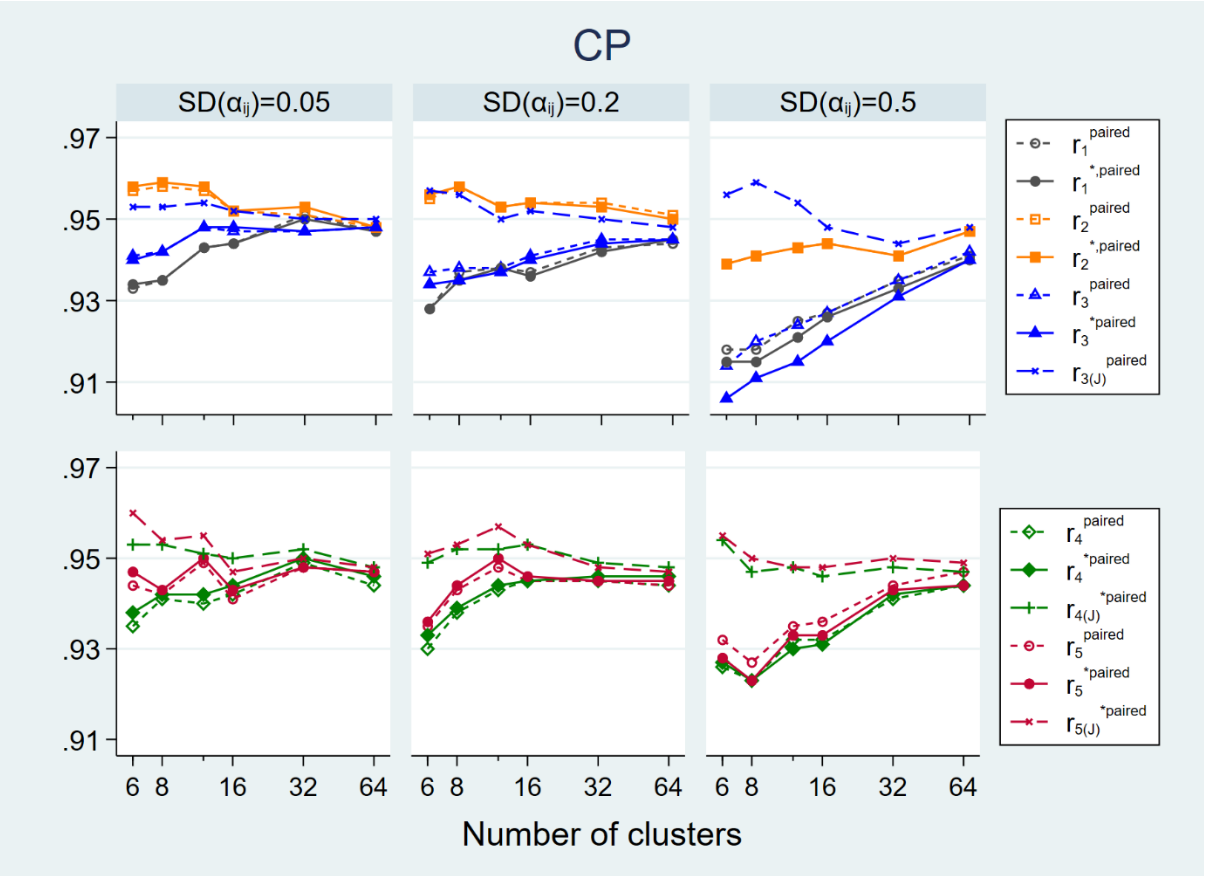

Figure S54. Coverage probability (CP) of 95% confidence interval (calculated on log-scale) of intervention effect estimators in relation to the number of clusters per trial arm for matched-pair CRTs, by three levels of $SD(\alpha_{ij})$; population size per cluster follows a skewed distribution with mean = 100 and $CV = 0.6$; intervention has direct and indirect effects $(\exp(\beta_D) = 0.5;\ \exp(\beta_I) = 0.75)$.

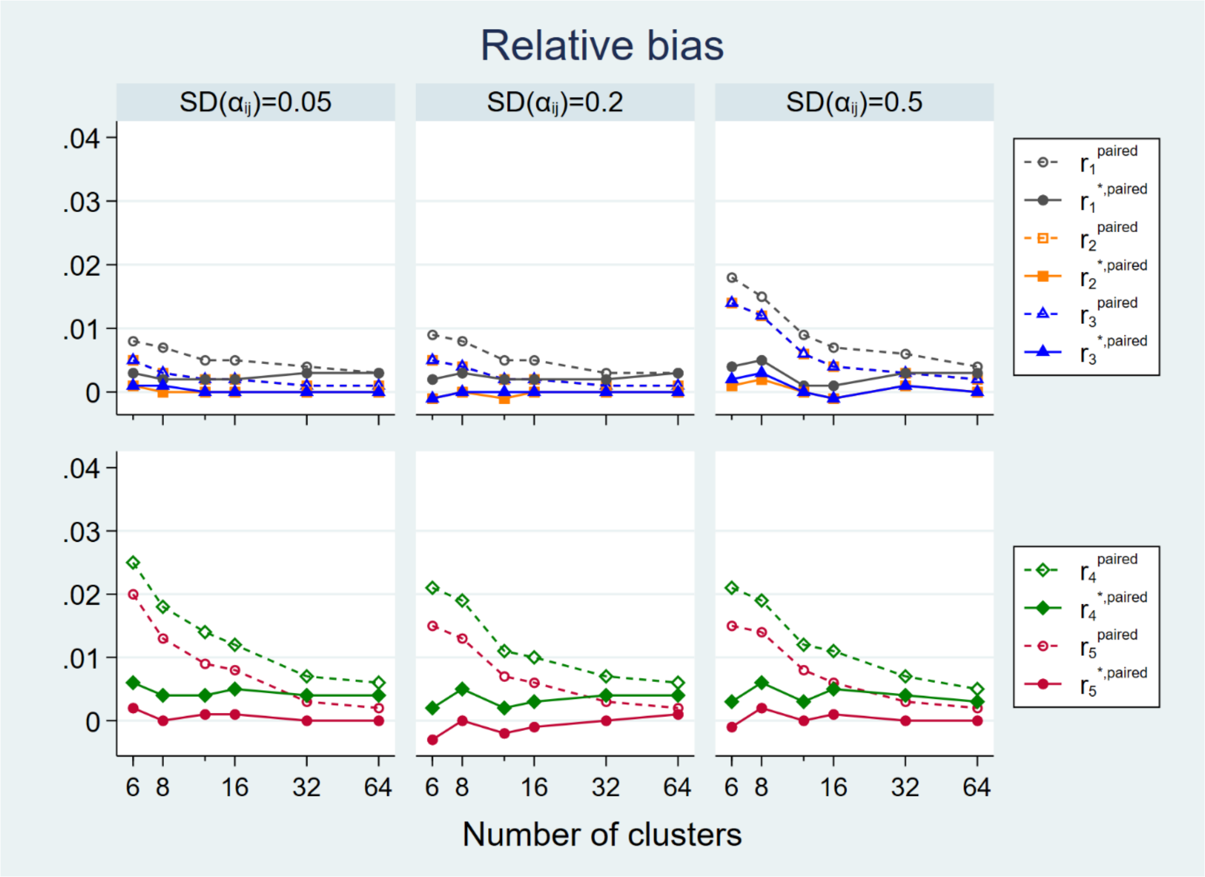

Figure S55. Relative bias of intervention effect estimators in relation to the number of clusters per trial arm for matched-pair CRTs, by three levels of $SD(\alpha_{ij})$; population size per cluster follows a skewed distribution with mean = 100 and $CV = 0.2$; intervention has no effect $(\exp(\beta_D) = 1;\ \exp(\beta_I) = 1)$.

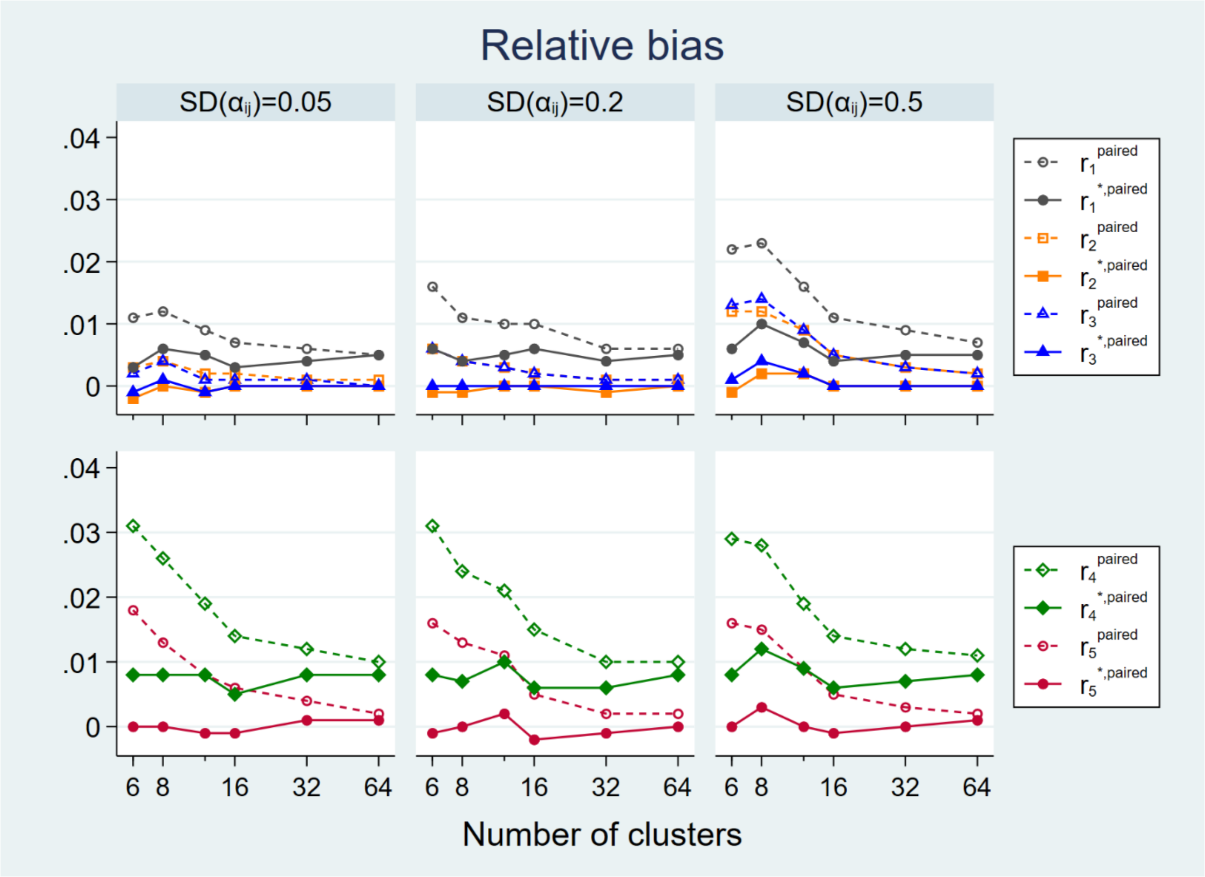

Figure S56. Relative bias of intervention effect estimators in relation to the number of clusters per trial arm for matched-pair CRTs, by three levels of $SD(\alpha_{ij})$; population size per cluster follows a skewed distribution with mean = 100 and $CV = 0.4$; intervention has no effect $(\exp(\beta_D) = 1;\ \exp(\beta_I) = 1)$.

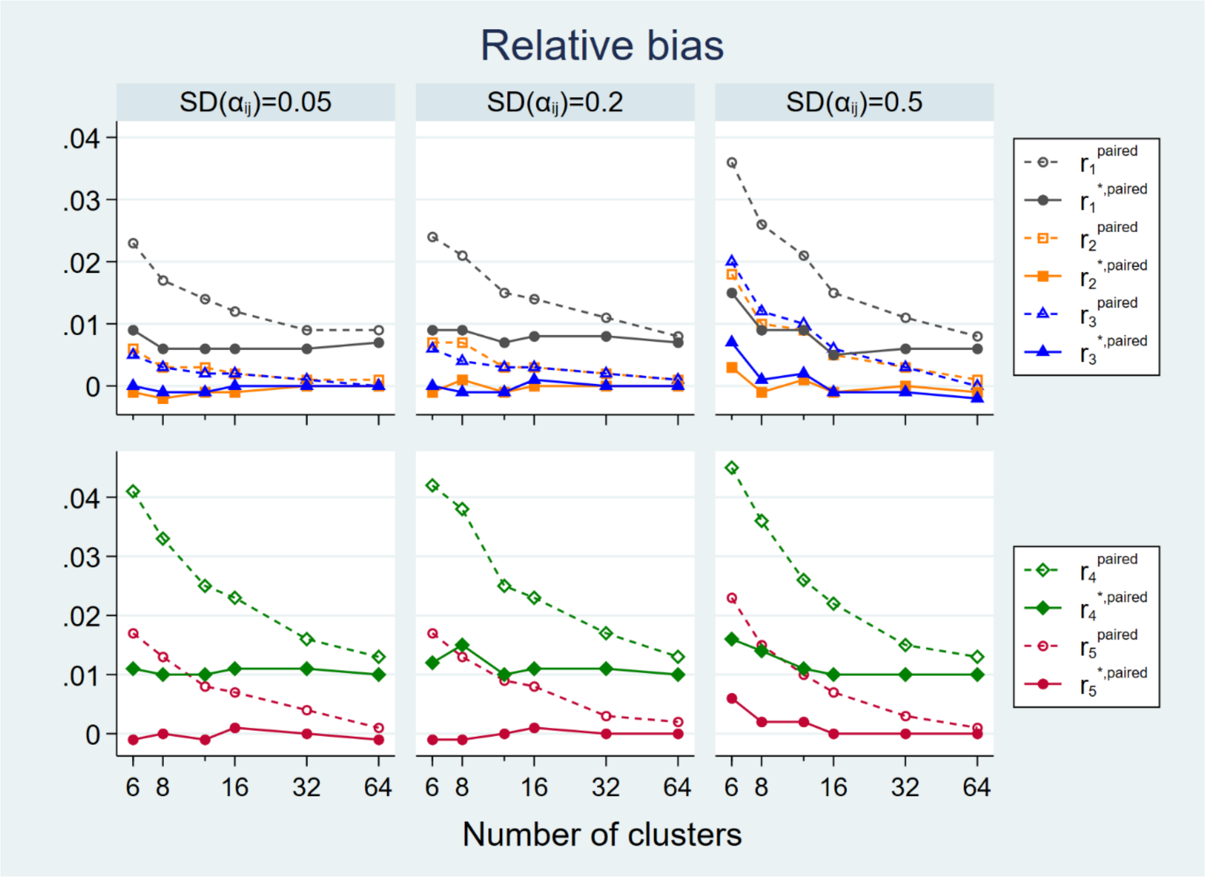

Figure S57. Relative bias of intervention effect estimators in relation to the number of clusters per trial arm for matched-pair CRTs, by three levels of $SD(\alpha_{ij})$; population size per cluster follows a skewed distribution with mean = 100 and $CV = 0.6$; intervention has no effect $(\exp(\beta_D) = 1;\ \exp(\beta_I) = 1)$.

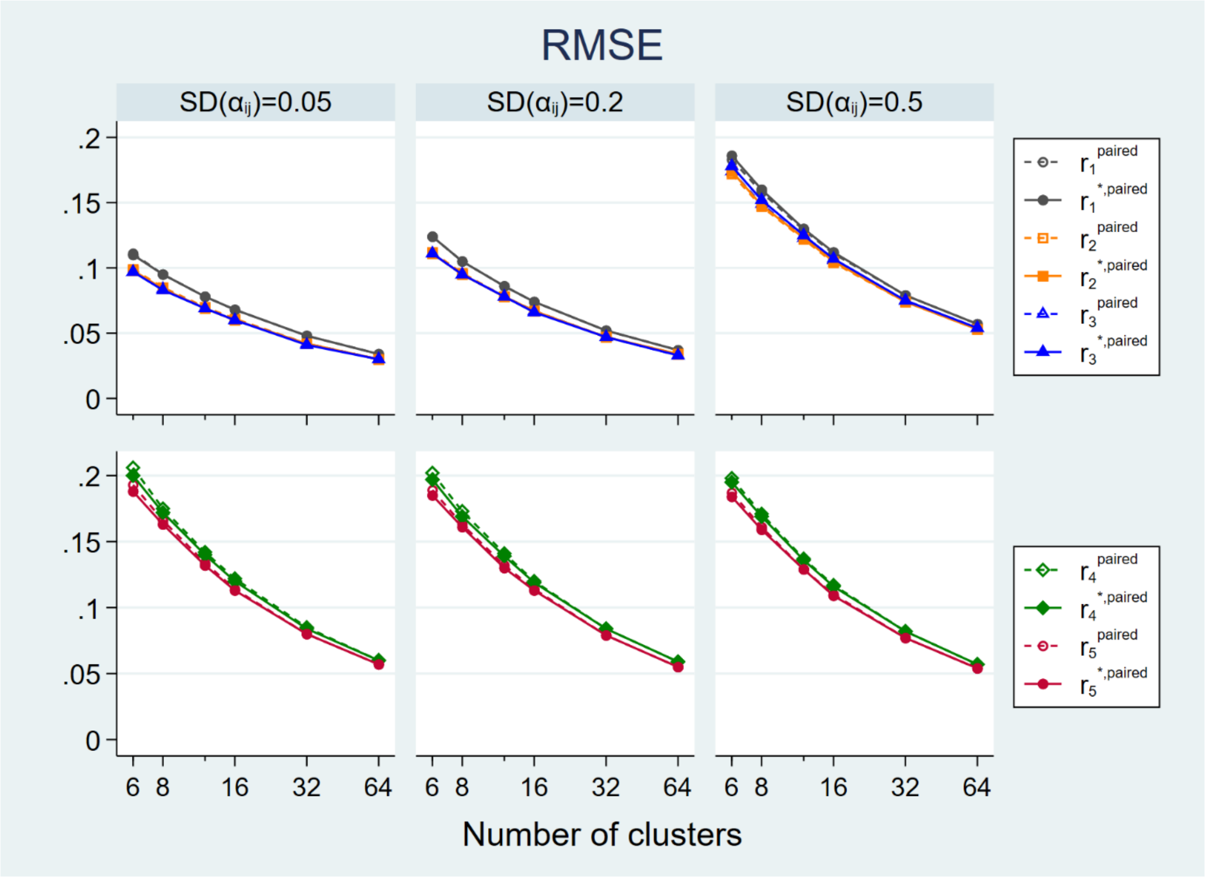

Figure S58. Root mean squared error (RMSE) of intervention effect estimators in relation to the number of clusters per trial arm for matched-pair CRTs, by three levels of $SD(\alpha_{ij})$; population size per cluster follows a skewed distribution with mean = 100 and $CV = 0.2$; intervention has no effect ($\exp(\beta_D) = 1;\ \exp(\beta_I) = 1$).

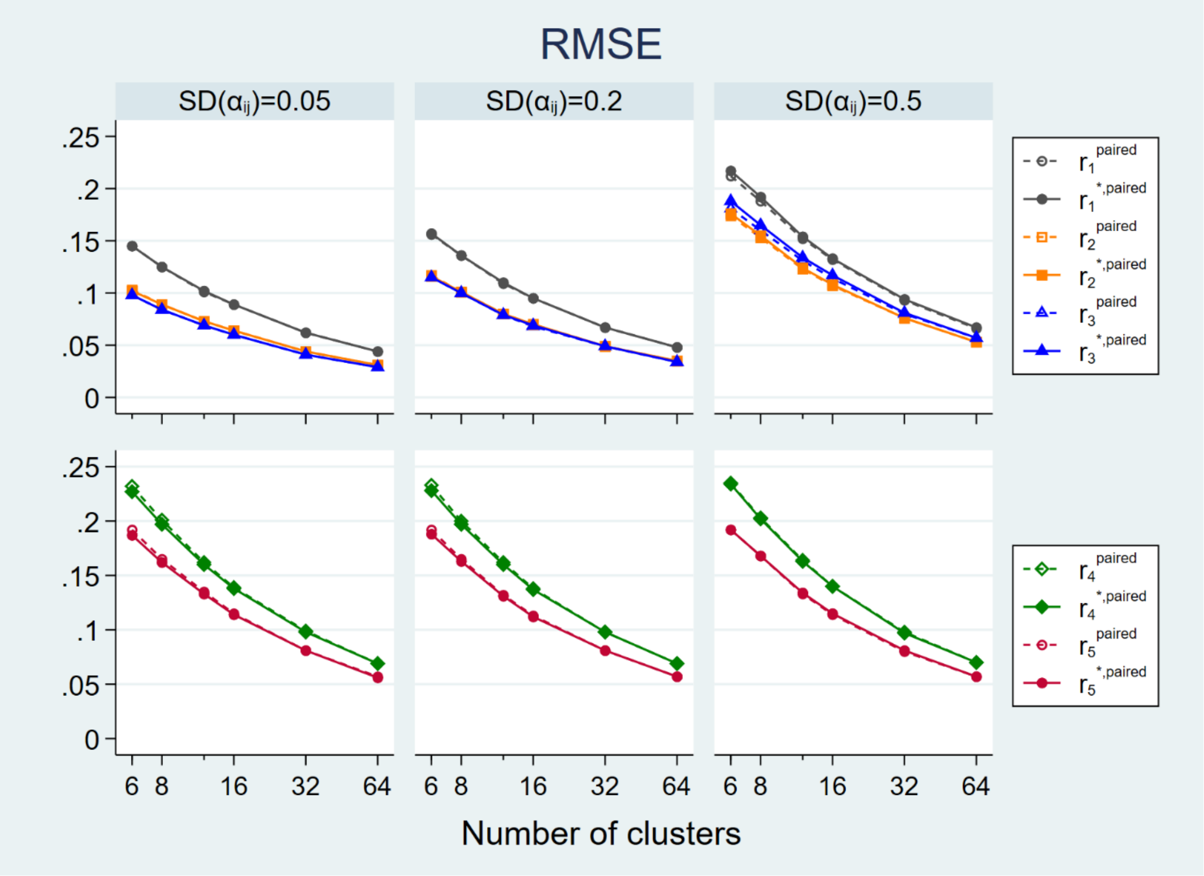

Figure S59. Root mean squared error (RMSE) of intervention effect estimators in relation to the number of clusters per trial arm for matched-pair CRTs, by three levels of $SD(\alpha_{ij})$; population size per cluster follows a skewed distribution with mean = 100 and $CV = 0.4$; intervention has no effect $(\exp(\beta_D) = 1;\ \exp(\beta_I) = 1)$.

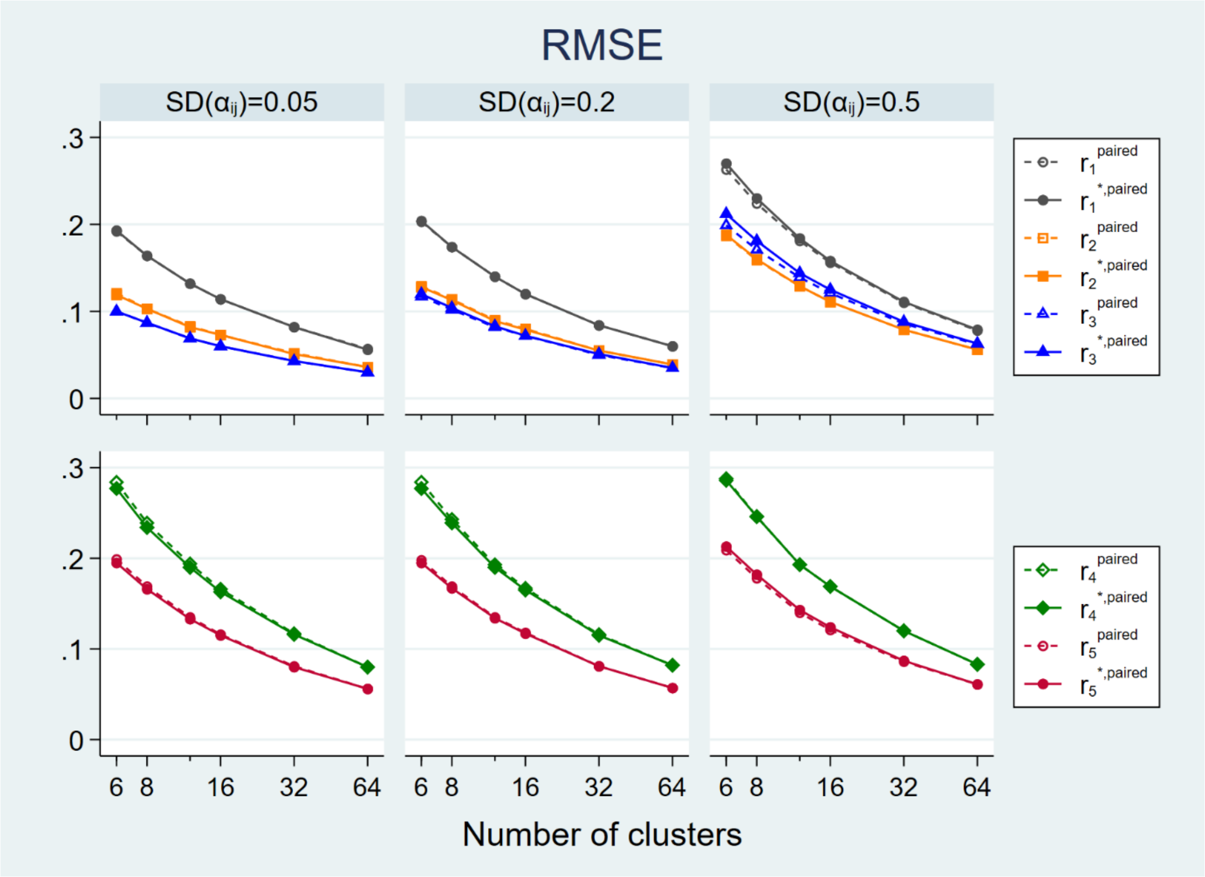

Figure S60. Root mean squared error (RMSE) of intervention effect estimators in relation to the number of clusters per trial arm for matched-pair CRTs, by three levels of $SD(\alpha_{ij})$; population size per cluster follows a skewed distribution with mean = 100 and $CV = 0.6$; intervention has no effect $(\exp(\beta_D) = 1;\ \exp(\beta_I) = 1)$.

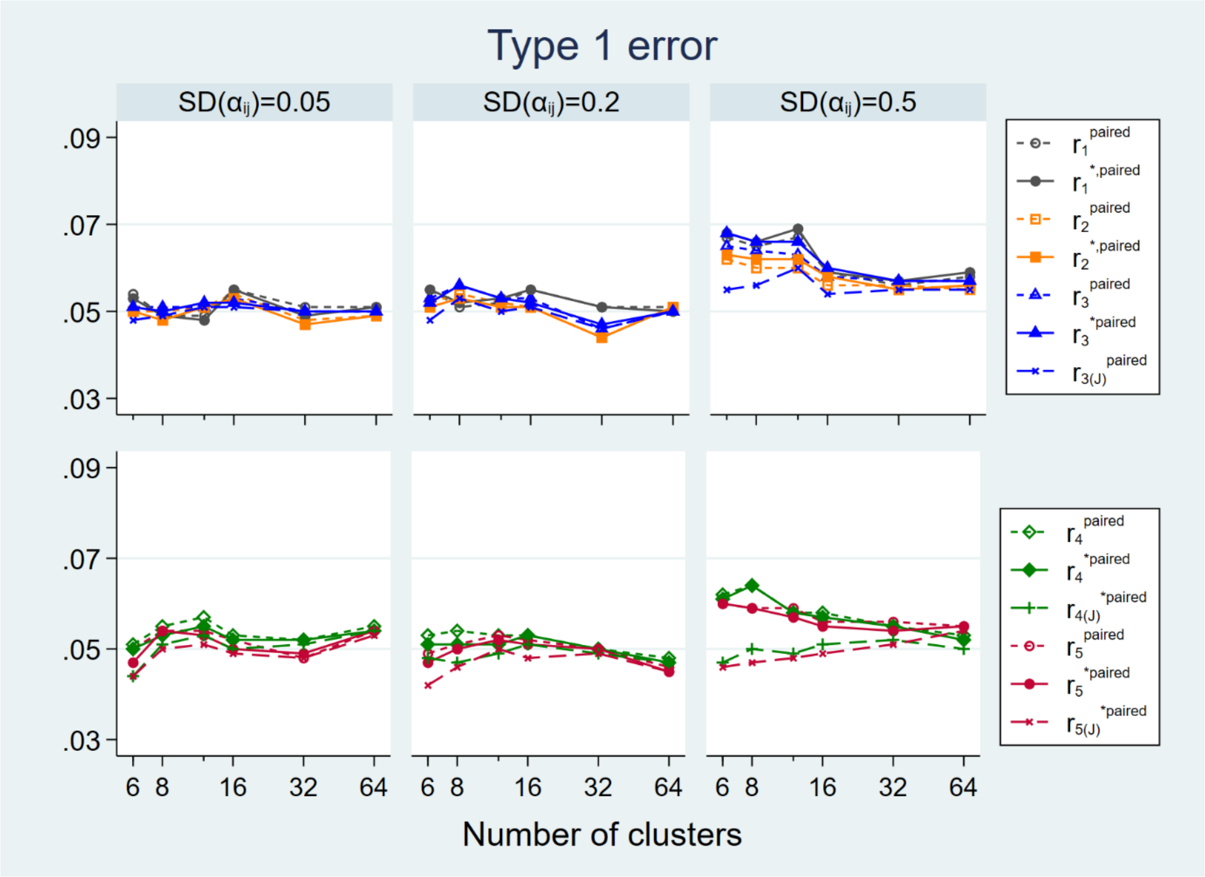

Figure S61. Type 1 error rate in relation to the number of clusters per trial arm for matched-pair CRTs, by three levels of $SD(\alpha_{ij})$; population size per cluster follows a skewed distribution with mean = 100 and $CV = 0.2$; intervention has no effect $(\exp(\beta_D) = 1;\ \exp(\beta_I) = 1)$.

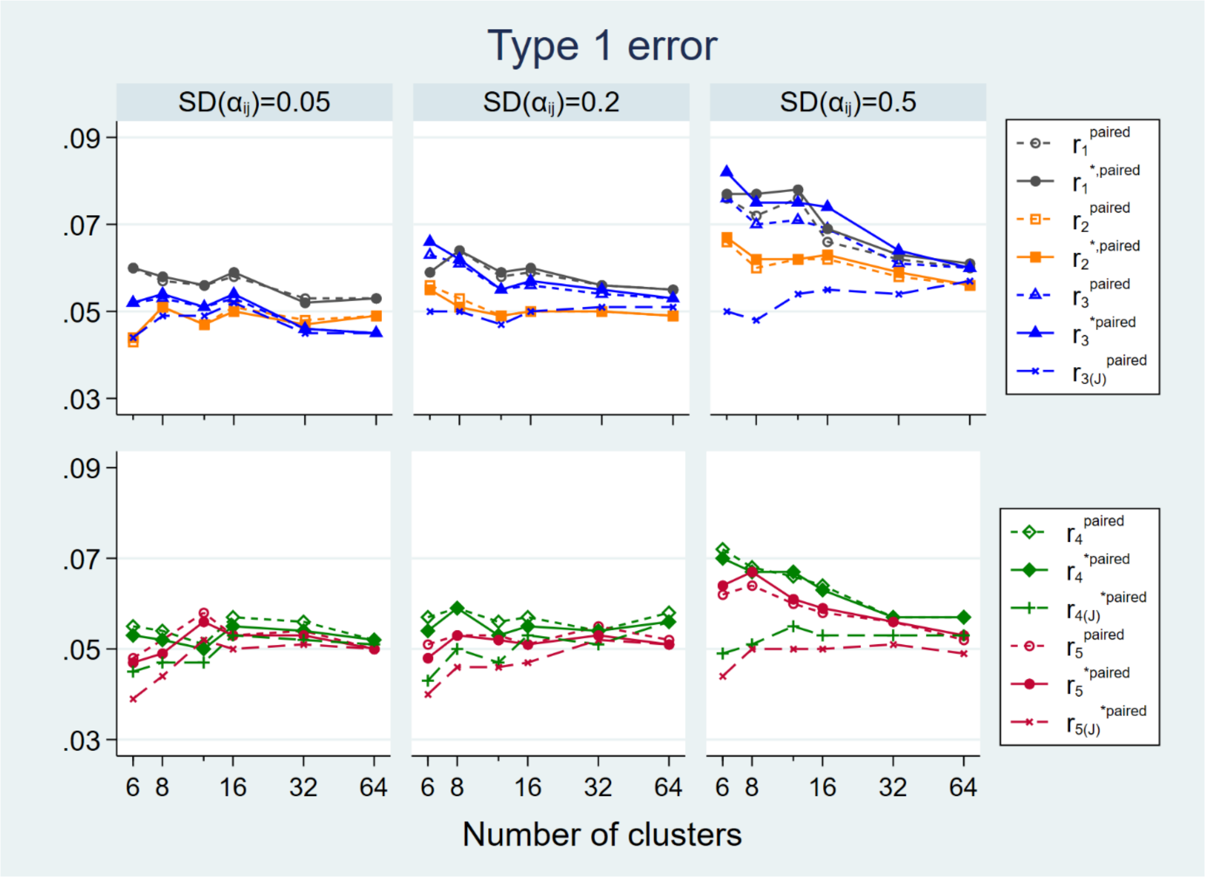

Figure S62. Type 1 error rate in relation to the number of clusters per trial arm for matched-pair CRTs, by three levels of $SD(\alpha_{ij})$; population size per cluster follows a skewed distribution with mean = 100 and $CV = 0.4$; intervention has no effect ($\exp(\beta_D) = 1;\ \exp(\beta_I) = 1$).

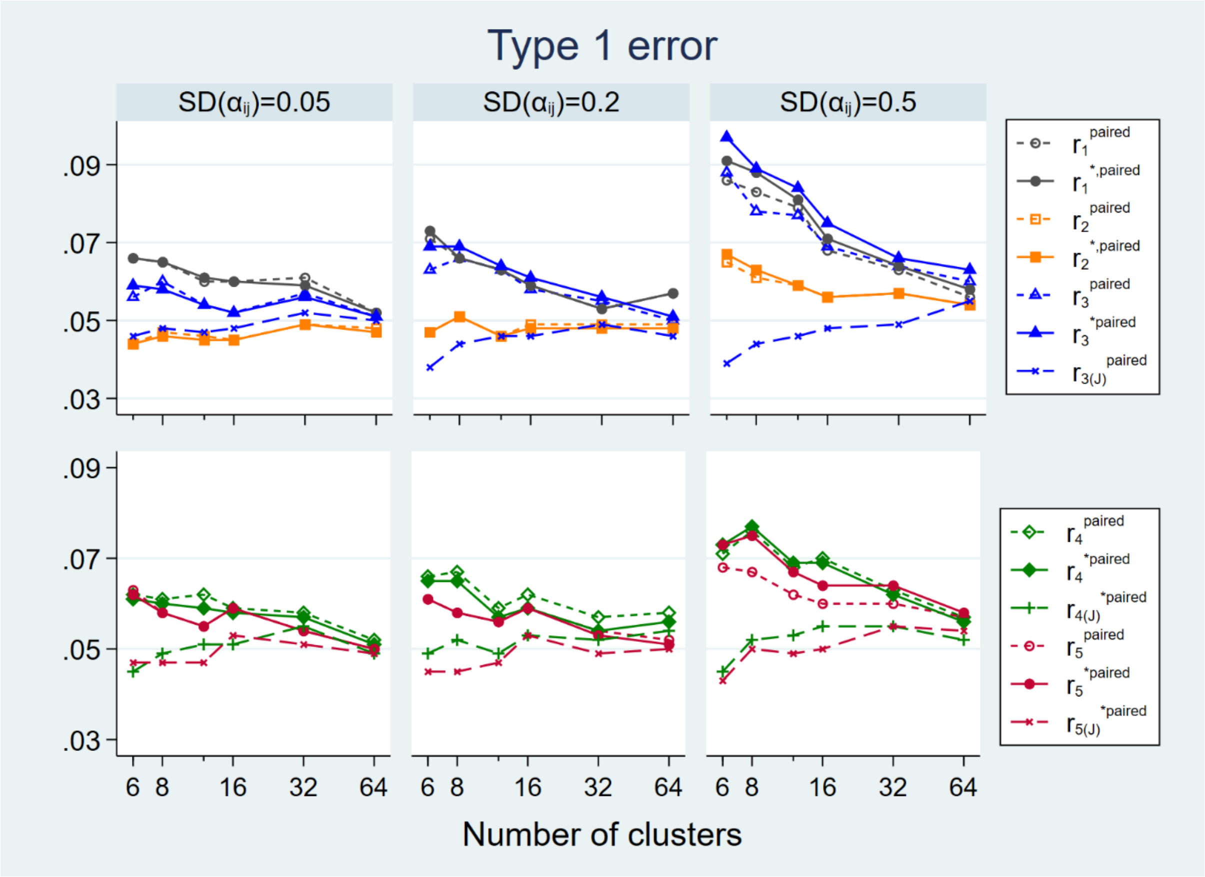

Figure S63. Type 1 error rate in relation to the number of clusters per trial arm for matched-pair CRTs, by three levels of $SD(\alpha_{ij})$; population size per cluster follows a skewed distribution with mean = 100 and $CV = 0.6$; intervention has no effect ($\exp(\beta_D) = 1;\ \exp(\beta_I) = 1$).

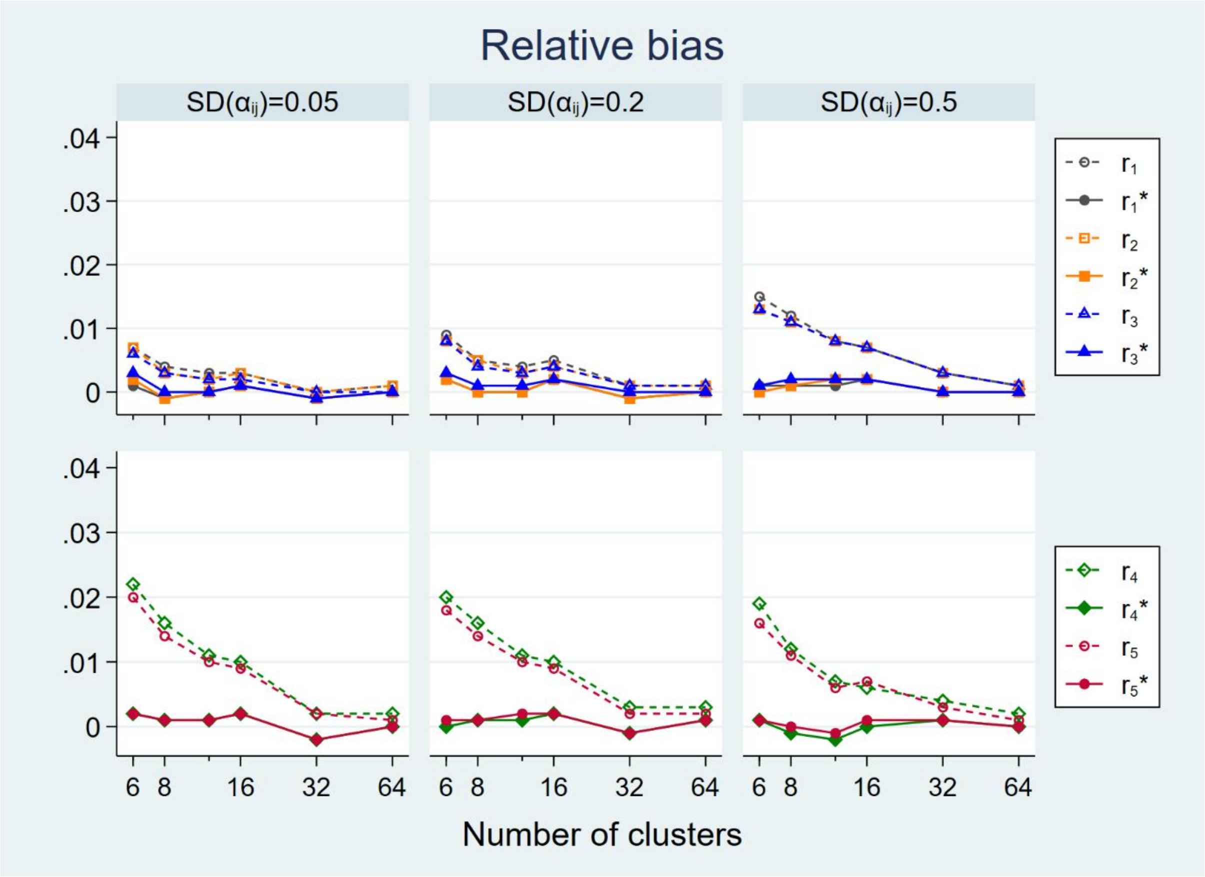

Figure S64. Relative bias of intervention effect estimators in relation to the number of clusters per trial arm for matched-pair CRTs, by three levels of $SD(\alpha_{ij})$; population size per cluster follows a normal distribution with mean = 100 and $CV = 0.2$; intervention has a direct effect only $(\exp(\beta_D) = 0.5;\ \exp(\beta_I) = 1)$.

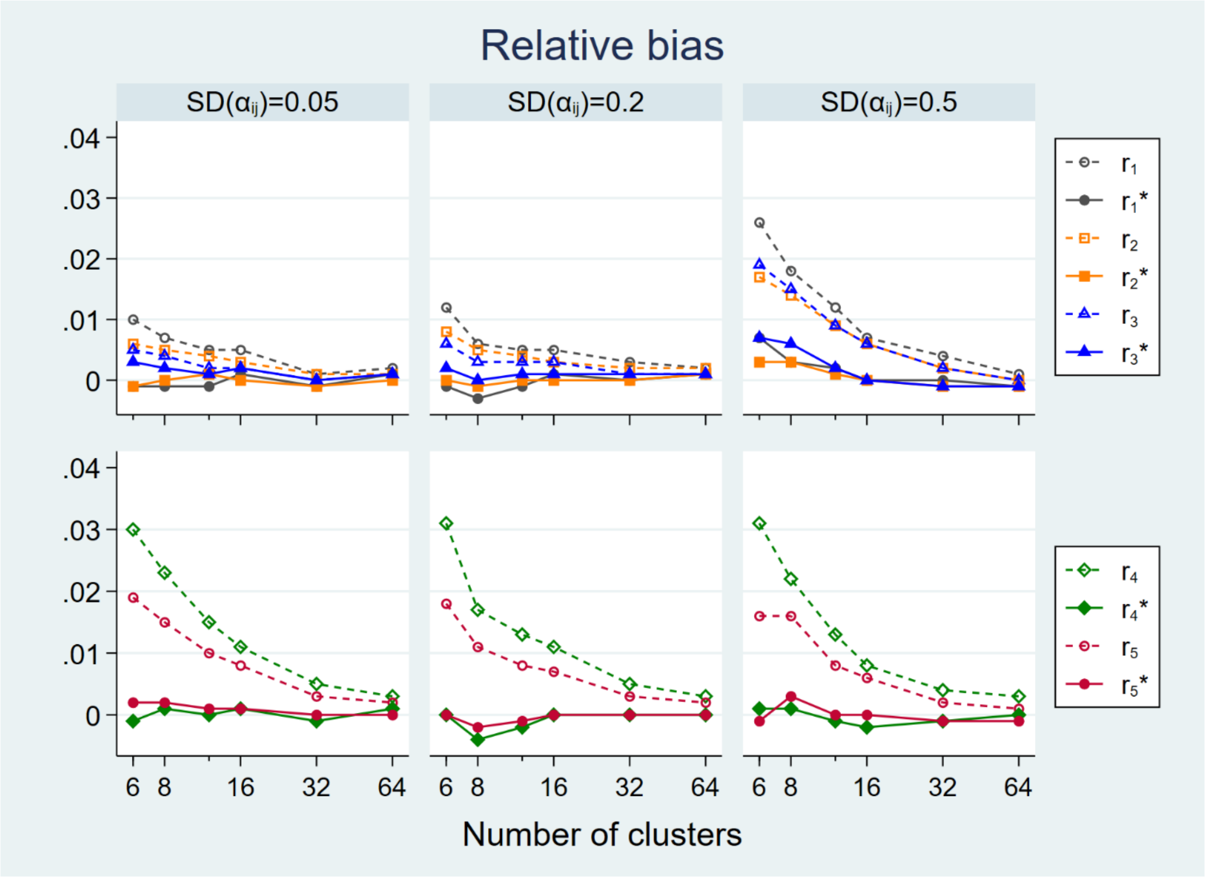

Figure S65. Relative bias of intervention effect estimators in relation to the number of clusters per trial arm for matched-pair CRTs, by three levels of $SD(\alpha_{ij})$; population size per cluster follows a normal distribution with mean = 100 and $CV = 0.4$; intervention has a direct effect only $(\exp(\beta_D) = 0.5;\ \exp(\beta_I) = 1)$.

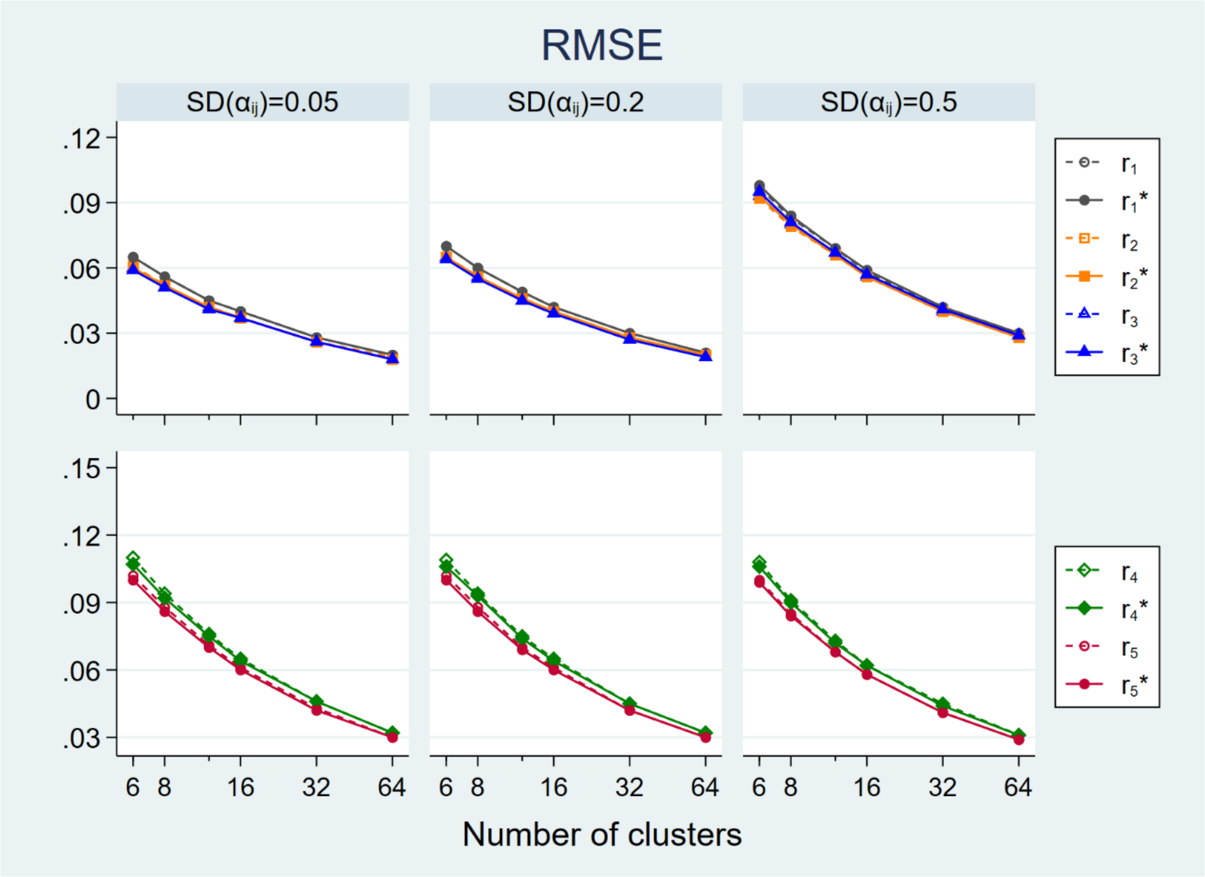

Figure S66. Root mean squared error (RMSE) of intervention effect estimators in relation to the number of clusters per trial arm for matched-pair CRTs, by three levels of $SD(\alpha_{ij})$; population size per cluster follows a normal distribution with mean = 100 and $CV = 0.2$; intervention has a direct effect only $(\exp(\beta_D) = 0.5;\ \exp(\beta_I) = 1)$.

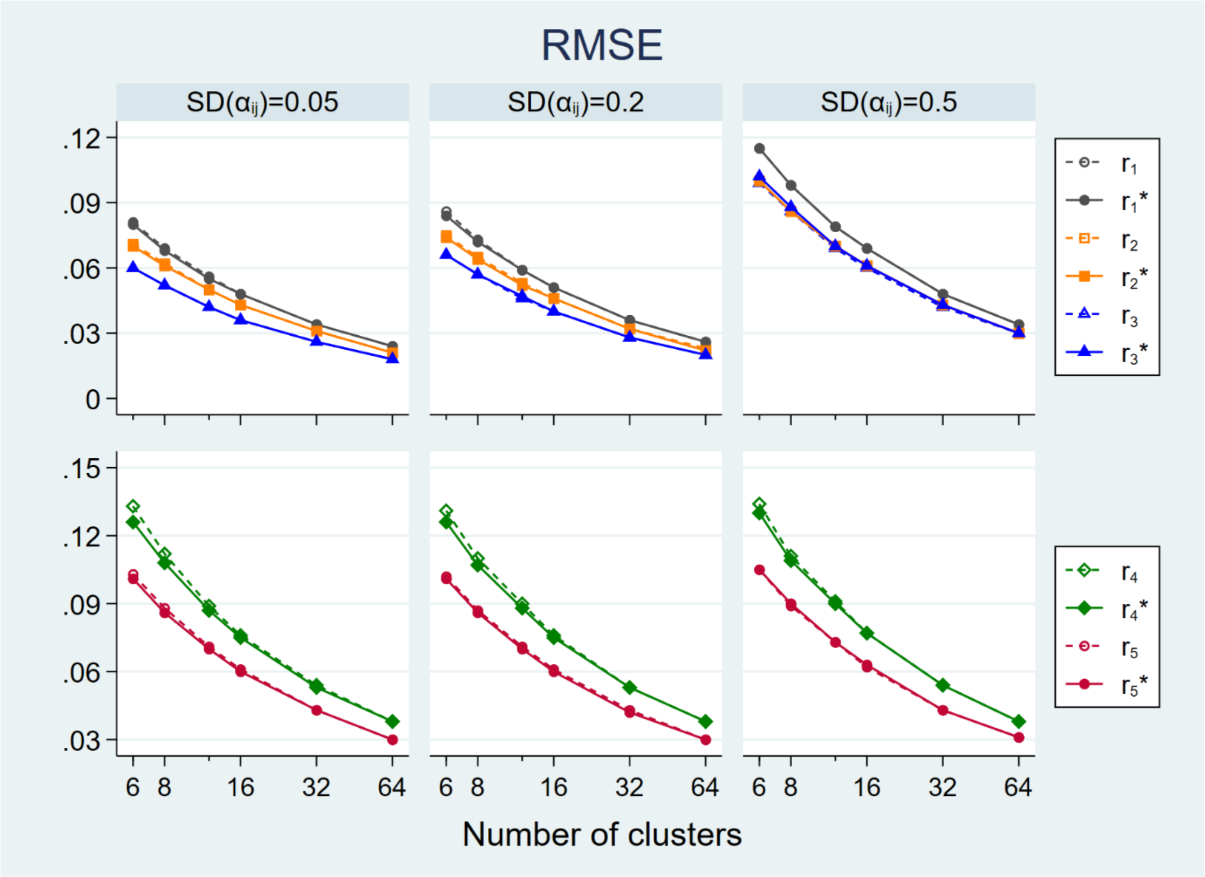

Figure S67. Root mean squared error (RMSE) of intervention effect estimators in relation to the number of clusters per trial arm for matched-pair CRTs, by three levels of $SD(\alpha_j)$; population size per cluster follows a normal distribution with mean = 100 and $CV = 0.4$; intervention has a direct effect only $(\exp(\beta_D) = 0.5;\ \exp(\beta_I) = 1)$.

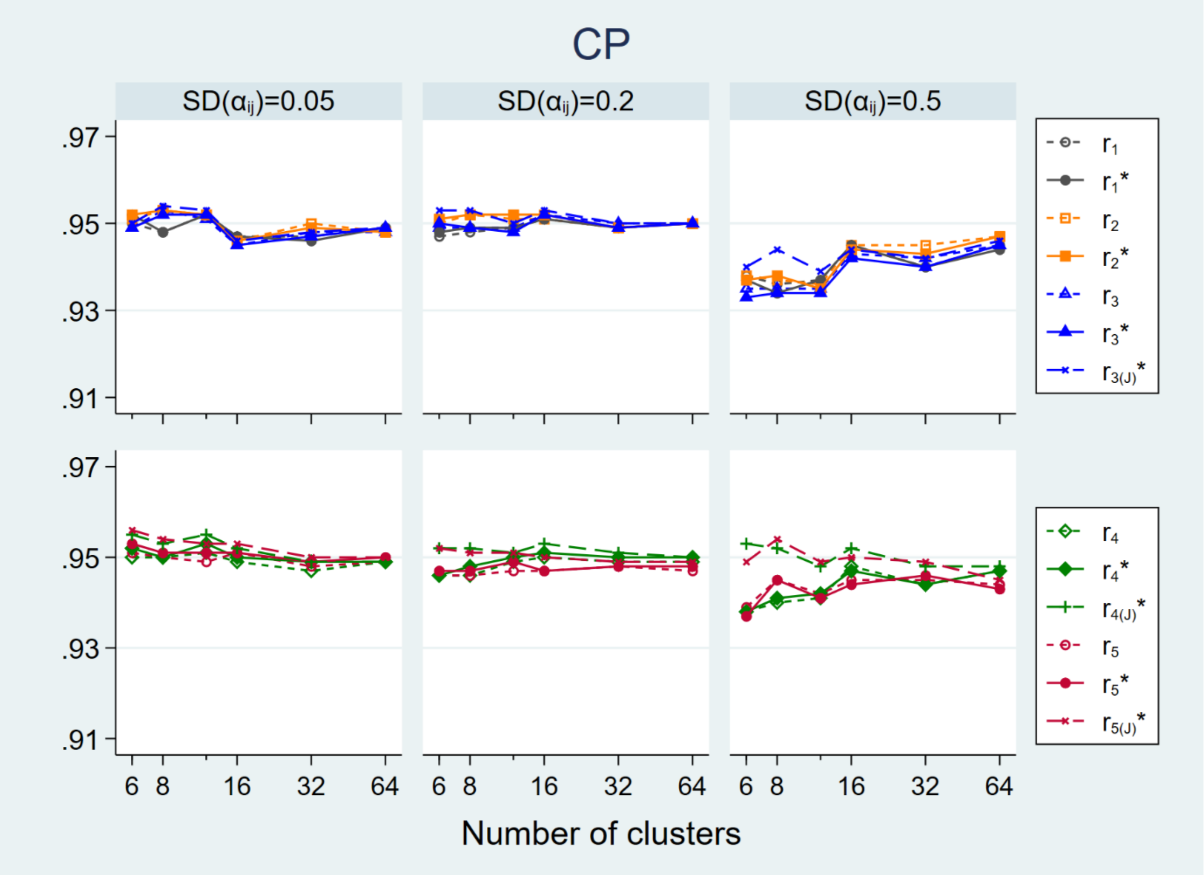

Figure S68. Coverage probability (CP) of 95% confidence interval (calculated on log-scale) of intervention effect estimators in relation to the number of clusters per trial arm for matched-pair CRTs, by three levels of $SD(\alpha_{ij})$; population size per cluster follows a normal distribution with mean = 100 and $CV = 0.2$; intervention has a direct effect only ($\exp(\beta_D) = 0.5;\ \exp(\beta_I) = 1$).

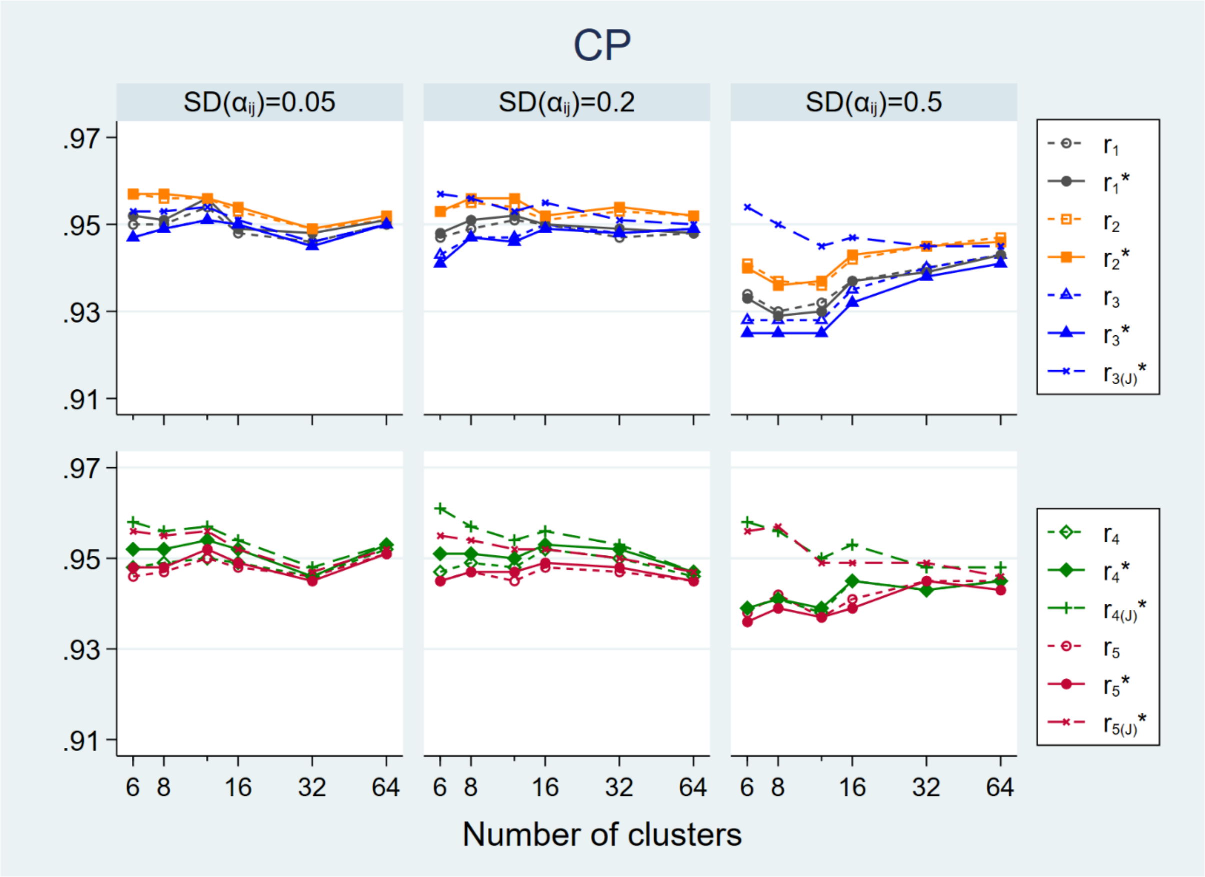

Figure S69. Coverage probability (CP) of 95% confidence interval (calculated on log-scale) of intervention effect estimators in relation to the number of clusters per trial arm for matched-pair CRTs, by three levels of $SD(\alpha_{ij})$; population size per cluster follows a normal distribution with mean = 100 and $CV = 0.4$; intervention has a direct effect only $(\exp(\beta_D) = 0.5;\ \exp(\beta_I) = 1)$.

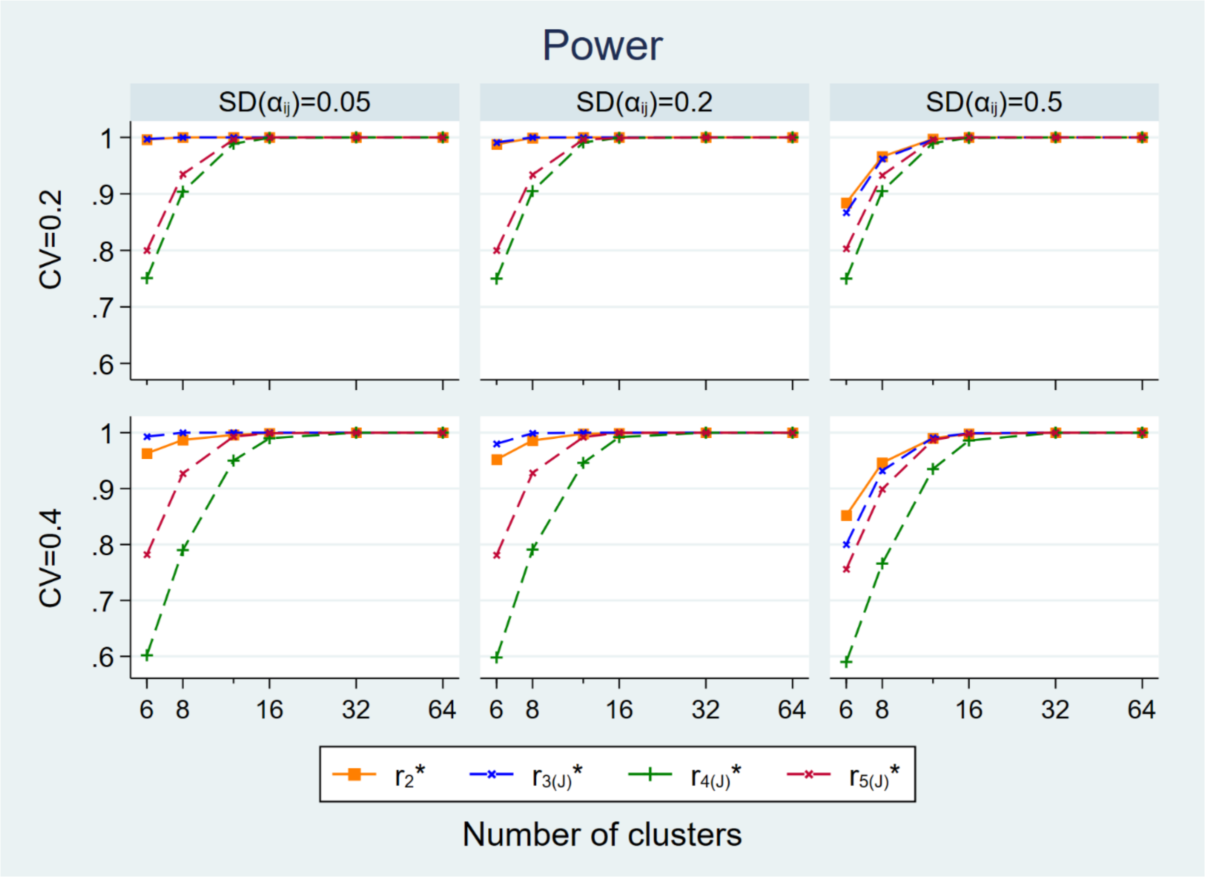

Figure S70. Power of $r_2^*, r_{3(J)}^*, r_{4(J)}^*$, and $r_{5(J)}^*$ in relation to the number of clusters per trial arm for matched-pair CRTs, by three levels of $SD(\alpha_{ij})$; population size per cluster follows a normal distribution with mean = 100 and $CV = 0.2, 0.4$, respectively; intervention has a direct effect only $(\exp(\beta_D) = 0.5;\ \exp(\beta_I) = 1)$. Upper panel: $CV = 0.2$; lower panel: $CV = 0.4$.
